# The Burden of Breast Cancer and its Attributable Risk Factors in 204 Countries and Territories, 1990–2021: Results From the Global Burden of Disease Study 2021

**DOI:** 10.1002/cai2.70055

**Published:** 2026-04-10

**Authors:** Jiahui Huang, Jiajin Li, Shengbin Pei, Cheng Zeng, Jiangdong Jin, Xun Hu, Jin Shi, Wei Dong, Ruimeng Wang, Yiqin Xia, Jiani Wang

**Affiliations:** ^1^ Department of Medical Oncology, National Cancer Center/National Clinical Research Center for Cancer/Cancer Hospital Chinese Academy of Medical Sciences and Peking Union Medical College Beijing China; ^2^ The Second Clinical School of Medicine Anhui Medical University Hefei China; ^3^ Department of Breast Surgery The First Affiliated Hospital of Nanjing Medical University Nanjing China; ^4^ Department of Diagnostic Imaging, National Cancer Center/Cancer Hospital Chinese Academy of Medical Sciences and Peking Union Medical College Beijing China

**Keywords:** breast cancer, disability‐adjusted life years, Global Burden of Disease, health inequality, risk factors, sociodemographic index

## Abstract

**Background:**

To report the global, regional, and national burden of breast cancer (BC) and its attributable risk factors between 1990 and 2021, by age, sex, and sociodemographic index.

**Methods:**

Using data from the Global Burden of Disease Study 2021, we analyzed BC prevalence, deaths, disability‐adjusted life years (DALYs), and attributable risk factors across 204 countries and territories from 1990 to 2021. Age‐standardized prevalence, deaths, and DALYs rates were estimated, and temporal trends were assessed using the estimated annual percentage change. Geographical and sociodemographic inequalities were further evaluated using decomposition analysis, concentration curves, and sociodemographic index (SDI)‐based modeling. Attributable risk factors for deaths and DALYs were quantified using the comparative risk assessment framework.

**Results:**

Globally, BC presents a starkly diverging landscape. While the age‐standardized prevalence has climbed to 239 per 100,000 (a 9.3% increase since 1990), the death and DALYs rates have actually declined by 13.7% and 9.8%, respectively. This global trend, however, masks a critical geographical shift. High‐income regions maintain the highest prevalence, yet the most rapid increases in burden are now concentrated in resource‐limited areas such as North Africa and the Middle East. The “triple threat” in low‐SDI regions defines this transition. Decomposition analysis revealed that while population growth and aging lead to absolute mortality everywhere, high‐SDI regions successfully offset this pressure through favorable epidemiological changes and advancements in screening and treatment. In contrast, low‐SDI regions face a deteriorating epidemiological profile that actively contributes to rising deaths. Our inequality analysis further underscores this systemic shift; concentration curves confirm that BC mortality is becoming disproportionately concentrated in lower‐SDI countries over time. Additionally, the disease follows a nonlinear, inverted U‐shaped relationship with socioeconomic development, peaking at an SDI of 0.75. Finally, we identified a distinct sex‐based etiological divide: while female risk patterns are multifaceted, male BC is almost singularly driven by metabolic dysregulation, with high body mass index emerging as the leading global driver of the disease.

**Conclusions:**

Despite progress in reducing the BC burden, it remains a global public health challenge. The prevalence is high in developed countries, while the burden is rapidly increasing in low‐ and middle‐income countries.

## Graphical abstract

This study integrates breast cancer data from the Global Burden of Disease across 204 countries and territories from 1990 to 2021, utilizing Bayesian hierarchical models to correct biases and quantify spatiotemporal trends. It analyzes geographic and population‐level disparities in risk factors, constructs a precision prevention framework based on rigorously quality‐controlled data, and proposes interventions for countries with varying income levels.

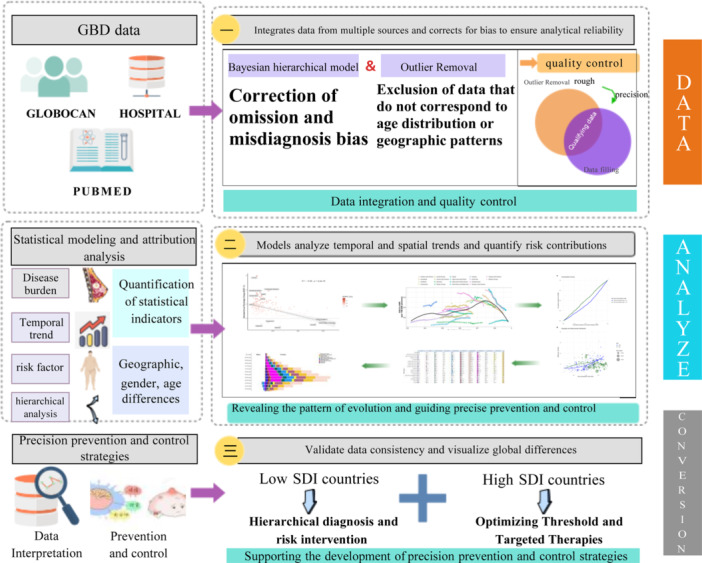

**Abbreviations**
BC: breast cancer
BMI: body mass index
CI: confidence interval
DALYs: disability‐adjusted life years
EAPC: estimated annual percentage change
GBD: Global Burden of Disease
SDI: sociodemographic index
UIs: uncertainty intervals

## 1 Introduction

Breast cancer (BC) is a common malignancy in women worldwide, characterized by abnormal cell proliferation and the risk of metastasis as it progresses [1]. Although certain risk factors are preventable through lifestyle modifications, once advanced, the disease is generally incurable; however, early screening and comprehensive treatment regimens can effectively control progression, reduce recurrence rates, and improve patient quality of life [2]. In 2022, female BC was the second most commonly diagnosed malignancy worldwide, the fourth leading cause of cancer‐related deaths globally, and the predominant cause of cancer deaths in 112 countries [3]. While the disease predominantly affects women, it also impacts the male population, constituting a small but distinct clinical burden. From 2005 to 2015, a 33% increase in the prevalence of BC was reported [4]. Despite extensive research outlining its epidemiological profile, significant geographical disparities persist worldwide, with high‐income countries reporting higher early detection rates due to the implementation of population‐wide mammography screening programs. In contrast, constrained healthcare infrastructure in African and Southeast Asian nations is associated with a predominance of advanced‐stage diagnoses, directly driving marked divergence in survival outcomes [5]. BC is pathologically defined by the malignant transformation of mammary epithelial cells and dysregulated proliferation, exhibiting marked molecular heterogeneity [6, 7]. Its pathogenesis involves an interconnected multifactorial risk network, including genetic predisposition, endocrine aberrations, metabolic dysregulation, lifestyle/dietary modifiers, iatrogenic exposures, progression of benign breast diseases, and socioeconomic determinants [8, 9, 10].

Multinational collaborative research consortia have driven the development of BC management systems. The International BC Study Group, established in 1978 through global multicenter clinical trials, has pioneered advances in optimizing adjuvant therapeutic strategies for early‐stage BC [11]. The European Society for Medical Oncology and the National Comprehensive Cancer Network are dedicated to advancing the diagnosis, treatment, and long‐term surveillance of early BC. Within this framework, the European Society for Medical Oncology has established tripartite diagnostic criteria integrating imaging, pathology, and molecular biology: Following mammography combined with ultrasonography suggestive of malignancy, comprehensive histopathological evaluation—supplemented by immunohistochemical and molecular profiling—is mandated, including assessment of estrogen receptor, progesterone receptor, human epidermal growth factor receptor 2 biomarkers, and proliferative markers. The standardized therapeutic framework encompasses locoregional interventions (surgery and radiotherapy) and systemic therapies, with treatment strategies formulated through the multidisciplinary integration of molecular subtyping, Tumor Node Metastasis staging, and individual patient status [12, 13, 14, 15].

Previous epidemiological studies on BC have documented a marked upward trend in global incidence rates. Multiregional population‐based studies spanning from 1990 to 2017 revealed a 123% increase in incident cases globally. Evidence suggests higher incidence rates in high‐income countries than in low‐income regions, potentially linked to the broader implementation of mammography screening programs [16]. Moreover, BC imposes a substantial economic burden, with direct medical costs per patient over their lifetime in the United States ranging from $20,000 to $100,000 [17]. Additionally, these costs are positively correlated with disease stage, increased recurrence frequency, and the use of novel therapeutic agents.

Moreover, existing literature primarily focuses on the female population. Men account for a smaller proportion of cases and thus represent a potentially overlooked area of tumors. Due to the lack of routine screening and low public awareness, male patients often present with advanced disease and have poorer survival rates. Therefore, analyzing both genders is not merely a statistical comparison but a clinical necessity for identifying differences in disease burden and access to healthcare.

Despite the wealth of existing literature on BC epidemiology, most previous studies rely on older datasets and focus primarily on incidence trends. A critical knowledge gap remains in understanding the structural drivers behind the shifting mortality burden, particularly in resource‐limited settings. This study addresses this gap by utilizing the Global Burden of Disease (GBD) 2021 data. Beyond updates, we employ sophisticated decomposition analysis and concentration curves to assess how population aging and epidemiological shifts contribute to the equalization of BC burden, with mortality pressure systematically migrating from high‐sociodemographic index (SDI) to lower‐SDI regions. By integrating Waterfall decomposition, concentration curves, and non‐linear SDI modeling, we aim to disentangle the drivers of the global survival gap and provide a precision‐public‐health framework for BC control in an era of shifting demographics and metabolic risks.

## 2 Methods

### 2.1 Data Source and Acquisition

The GBD 2021 provided publicly available data to analyze the prevalence, deaths, and disability‐adjusted life years (DALYs) of BC among individuals aged 20 years and older from 1990 to 2021 across 204 countries and territories. Data were extracted in April 2025 from the GBD Collaborative Network's public database (https://ghdx.healthdata.org/gbd-results-tool). The extraction query parameters included: Cause ID: BC (C50); Location: Global, 204 countries and territories; Year: 1990–2021 (annual); Age: 5‐year groups (20–24 to 95+); Sex: Both sexes; Measure: Prevalence, Deaths, DALYs.

### 2.2 Case Definition and Data Sources

Cases were defined as histopathologically confirmed invasive breast carcinomas (ICD‐10: C50) or imaging‐supported malignancies meeting American Joint Committee on Cancer clinical staging criteria. The staging definitions used in this study adhered to the American Joint Committee on Cancer classification: T (primary tumor): size, multifocality, skin satellite nodules (e.g., T1 ≤ 2 cm, T2 = 2–5 cm, T3 > 5 cm, T4 invasion of skin/chest wall); N (regional lymph nodes): number and size of metastatic lymph nodes (e.g., N0 no metastasis, N1 = 1–3 nodes, N2 = 4–9 nodes, N3 ≥ 10 nodes); M (distant metastasis): M0 no metastasis, M1 present metastasis [18]. Data integration includes the following sources:

1. *Cancer registries*: Age‐ and sex‐stratified prevalence and death data were obtained from the Global Cancer Observatory and country‐specific cancer surveillance networks.
2. *Vital registration and verbal autopsy*: Data on cause of death were integrated from vital registration systems worldwide.
3. *Systematic literature reviews*: The GBD team conducted systematic reviews of epidemiological studies to supplement data in regions with sparse registry coverage.
4. *Clinical data*: Hospital records were utilized by GBD to inform disability weights and stage distribution.

To address data scarcity and underreporting biases, particularly in low‐ and middle‐income countries, the GBD study employed the Bayesian meta‐regression tool, DisMod‐MR 2.1. By integrating predictive covariates (e.g., SDI) and “borrowing strength” from spatially and temporally adjacent regions, this tool generated robust estimates for locations with sparse data while enforcing epidemiological consistency among incidence, prevalence, and mortality measures. Furthermore, misclassification biases were mitigated by using standardized algorithms to redistribute “garbage codes” (ill‐defined causes of death), ensuring cross‐national comparability of the final dataset.

### 2.3 Statistical Models and Analytical Methods

#### 2.3.1 Disease Burden Analysis

The estimation of BC prevalence, mortality, and DALYs was performed by the GBD 2021 collaborative network using the standardized Bayesian meta‐regression tool, DisMod‐MR 2.1. This tool ensured consistency between incidence, prevalence, remission, and mortality measures by integrating predictive covariates (e.g., SDI, healthcare access). Years Lived with Disability were calculated by multiplying the prevalence of each sequela by its disability weight. Years of Life Lost were estimated by multiplying the number of deaths by the remaining life expectancy at the age of death from the GBD standard life table. The sum of years of Life Lost and years Lived with Disability constituted the DALYs.

#### 2.3.2 Age‐Standardized Rates

The age‐standardized rates, including the age‐standardized prevalence rate, mortality rate, and DALY rate, were calculated using the GBD global standard population structure to eliminate distortions from population aging in trend analysis.

#### 2.3.3 Spatiotemporal Trend Analysis

To quantify the temporal trends in ASRs from 1990 to 2021, we calculated the estimated annual percentage change (EAPC). A regression line was fitted to the natural logarithm of the ASRs:

(1) ln(ASR)=α+βx+ϵ,

where ln(ASR) is the natural logarithm of the age‐standardized rate, *x* represents the calendar year, and *ε* denotes the error term. The EAPC was calculated as follows:

(2) EAPC=100×(exp(β)−1).

The 95% confidence interval (CI) of the EAPC was obtained from the linear regression model. A trend was considered significantly increasing if the EAPC estimate and its 95% CI lower bound were > 0, and decreasing if the EAPC estimate and its 95% CI upper bound were < 0. Otherwise, the trend was deemed stable.

#### 2.3.4 Decomposition Analysis

To evaluate the drivers of the change in the number of BC deaths from 1990 to 2021, we performed a decomposition analysis using the Das Gupta method. This method decomposes the total change into three contributing factors: population growth, population aging, and changes in age‐specific death rates. This allows us to quantify the relative contribution of demographic versus epidemiological shifts.

#### 2.3.5 Concentration Curves

To assess the shift in global BC burden equity, we utilized concentration curves. The countries were ranked by their SDI on the *x*‐axis, and the cumulative proportion of the BC death burden was plotted on the *y*‐axis. A curve shifting further away from the diagonal line of equality indicates an increasing concentration of mortality burden in specific SDI tiers.

#### 2.3.6 Risk Factor Attribution

We quantified the burden of BC attributable to modifiable risk factors using the comparative risk assessment framework developed by the GBD study. This analysis estimates the burden attributable to specific risks by comparing current exposure levels to a theoretical minimum risk exposure level—the counterfactual level of exposure that minimizes risk at the population level [16]. The population attributable fraction for each risk factor (e.g., alcohol use, smoking, high fasting plasma glucose) was calculated using the following equation:

(3) $$\mathrm{PAF}=\frac{\sum \left(P_{i}\times {\mathrm{RR}}_{i}\right)-1}{\sum \left(P_{i}\times {\mathrm{RR}}_{i}\right)},$$

where $P_{i}$ represents the prevalence of the exposure level i, and ${\mathrm{RR}}_{i}$ is the relative risk derived from meta‐analyses. Combined “behavioral risk” metrics were used to avoid double‐counting, accounting for the mediation of risks such as physical inactivity on body mass index (BMI). Specifically, behavioral risks in this study include high BMI, alcohol use, and tobacco smoking, which are grouped according to the GBD risk hierarchy to assess their collective impact while accounting for overlapping mediation effects.

### 2.4 Uncertainty Analysis

Uncertainty intervals (UIs) for all estimates were generated through 1000 Monte Carlo simulations. The 95% UI boundaries were defined by truncating ordered bootstrap resamples and selecting the 2.5th percentile and 97.5th percentile values. Nonlinear associations between BC‐related DALYs and the SDI were examined using smoothing spline regression models. Results were visualized with R software (version 4.4.2).

### 2.5 Research Ethics

The study utilized de‐identified public population health statistics. No patient representatives were involved in the design of the core study components.

## 3 Results

### 3.1 Global Level

In 2021, 20.6 million prevalent cases of BC were reported globally (Table 1), with an age‐standardized point prevalence of 238.9 per 100,000, representing a 9.3% increase since 1990. BC accounted for 670,000 deaths in 2021, with an age‐standardized rate of 7.9, a decrease of 13.7% since 1990. In 2021, the number of DALYs for BC globally was 20.59 million, with an age‐standardized rate of 239 DALYs per 100,000, a 9.8% decrease since 1990 (Table 1).

**Table 1:** Prevalence cases, deaths, and disability‐adjusted life years (DALYs) for breast cancer in 2021, and percentage change in age‐standardized rates (ASRs) per 100,000, by Global Burden of Disease region, from 1990 to 2021 (generated from data available at External, opens in a new tab. https://ghdx.healthdata.org/gbd-results-tool).

	Prevalence (95% UI)			Deaths (95% UI)			DALYs (95% UI)		
	No. in millions (95% UI)	ASRs per 100,000 (95% UI)	Percentage change in ASRs from 1990 to 2021	No. in thousands (95% UI)	ASRs per 100,000 (95% UI)	Percentage change in ASRs from 1990 to 2021	No. in thousands (95% UI)	ASRs per 100,000 (95% UI)	Percentage change in ASRs from 1990 to 2021
Global	20.6 (19.5, 21.8)	238.9 (226.2, 252.2)	9.3 (0.1, 18.2)	673.6 (622.7, 720.2)	7.9 (7.3, 8.4)	−13.7 (−18.1, −8.9)	20587.8 (19311.7, 21942.3)	239.0 (224.2, 254.9)	−9.8 (−15.5, −3.5)
High‐income Asia Pacific	1.2 (1.1, 1.2)	319.4 (296.4, 339.6)	58.3 (42.5, 73.2)	20.2 (16.8, 22.2)	4.9 (4.3, 5.2)	27.3 (16.6, 34.4)	542.0 (483.1, 588.0)	163.1 (149.6, 175.5)	18.6 (10.5, 25.1)
High‐income North America	3.3 (3.1, 3.5)	543.6 (514.6, 570.3)	−20.1 (−24.3, −15.9)	59.6 (53.3, 63.2)	9.3 (8.5, 9.8)	−41.2 (−43.8, −39.0)	1573.2 (1465.4, 1678.0)	273.5 (256.2, 290.0)	−43.0 (−45.2, −40.9)
Western Europe	3.8 (3.6,4)	476.5 (453.3, 496.5)	−1.2 (−8.9, 6.3)	93.9 (80.7, 101.2)	9.9 (8.8, 10.6)	−40.4 (−44.0, −37.6)	2147.4 (1938.2, 2312.0)	274.3 (252.5, 293.5)	−41.9 (−44.5, −39.4)
Australasia	0.2 (0.2, 0.2)	465.3 (427.9, 503.6)	−1.3 (−11.6, 9.7)	4.4 (3.7, 4.9)	8.3 (7.3, 9.3)	−42.5 (−48.2, −36.0)	113.4 (101.2, 126.3)	244.6 (220.6, 271.2)	−42.1 (−47.2, −36.5)
Andean Latin America	0.1 (0.1, 0.1)	143.2 (115.3, 176.4)	83.8 (48.3, 126.9)	4.1 (3.3, 5.2)	6.8 (5.4, 8.6)	7.8 (−13.7, 33.4)	129.3 (102.4, 163.0)	206.6 (163.9, 259.5)	5.8 (−15.1, 31.7)
Tropical Latin America	0.5 (0.5, 0.5)	197.1 (186.1, 207.5)	59 (49.9, 68.6)	24.4 (22.6, 25.9)	9.4 (8.7, 10.0)	4.9 (−0.5, 10.5)	758.4 (712.3, 797.2)	287.8 (269.9, 302.6)	7.2 (1.6, 13.2)
Central Latin America	0.7 (0.6, 0.7)	248.9 (219.7, 278.9)	96.9 (73.6, 122.4)	19.5 (17.0, 21.9)	7.6 (6.7, 8.6)	20.2 (5.6, 35.5)	635.3 (550.2, 716.8)	241.7 (209.7, 272.7)	23.5 (7.5, 39.7)
Southern Latin America	0.2 (0.2, 0.2)	264.3 (248.1, 280.7)	9.8 (−1.7, 21.1)	10.1 (9.1, 10.9)	11.6 (10.5, 12.6)	−26.4 (−32.4, −19.6)	263.4 (242.5, 283.1)	317.7 (292.7, 341.5)	−28.0 (−33.7, −22.0)
Caribbean	0.1 (0.1, 0.1)	239.1 (208.8, 270.2)	28.4 (13.1, 45.2)	5.7 (4.8, 6.7)	10.6 (8.9, 12.4)	3.8 (−10.1, 18.4)	166.5 (139.0, 197.4)	314.1 (261.8, 372.9)	3.0 (−11.5, 18.9)
Central Europe	0.6 (0.6, 0.7)	311.9 (291.5, 333.1)	30.8 (18.2, 44.7)	25.2 (23.0, 27.3)	11.6 (10.6, 12.6)	−5.2 (−12.7, 3.0)	609.7 (562.4, 659.6)	308.6 (284.0, 335.3)	−13.2 (−20.4, −5.3)
Eastern Europe	0.9 (0.9, 1.0)	283.8 (258.0, 310.1)	20.4 (8.2, 34.2)	36.1 (32.3, 40.8)	10.5 (9.4, 11.9)	−4.2 (−14.3, 7.6)	990.2 (884.1, 1123.0)	304.4 (270.9, 346.4)	−12.4 (−22.4, −1.2)
Central Asia	0.1 (0.1, 0.2)	156.2 (143.3, 169.9)	−10.4 (−20.2, −0.1)	6.6 (5.9, 7.4)	7.8 (7.0, 8.6)	−27.6 (−35.5, −19.1)	219.0 (194.7, 246.2)	236.1 (210.6, 264.0)	−30.7 (−38.9, −21.9)
North Africa and Middle East	1.2 (1.1, 1.3)	221.4 (201.5, 242.4)	143.7 (111.5, 178.7)	30.1 (26.5, 34.2)	6.1 (5.4, 6.9)	62.8 (43.3, 85.7)	1081.5 (942.2, 1242.4)	194.9 (170.8, 223.5)	57.0 (36.1, 81.5)
South Asia	1.6 (1.5, 1.9)	99.1 (88.4, 111.4)	96.9 (71.3, 125.7)	107.8 (94.1, 123.1)	6.9 (6.1, 7.9)	50.5 (28.1, 76.4)	3757 (3282.3, 4303.6)	223.5 (195.2, 255.9)	46.6 (25.4, 72.4)
Southeast Asia	1.2 (1.0, 1.4)	162.3 (141.4, 188.6)	80 (56, 106.5)	66.2 (55.0, 80.7)	9.6 (8.0, 11.5)	32.7 (13.5, 56.5)	2251.6 (1862.2, 2760.0)	302.6 (250.7, 369.4)	29.3 (9.2, 53.6)
East Asia	4.0 (3.3, 4.9)	186.5 (153.1, 226.5)	107.5 (61.9, 170.5)	96.4 (76.5, 119.0)	4.5 (3.6, 5.5)	−5 (−28.9, 28.8)	3185.6 (2514.9, 3998.8)	148.7 (117.2, 186.7)	−2.3 (−28.3, 34.4)
Oceania	0 (0, 0)	127.9 (109.7, 152.2)	9.8 (−7.9, 32.9)	1.0 (0.8, 1.2)	10.9 (9.1, 13.2)	12.6 (−9.1, 42.5)	36.0 (28.9, 45.9)	352.8 (289.4, 436.8)	13.8 (−12.1, 48.4)
Western Sub‐Saharan Africa	0.3 (0.2, 0.4)	128.5 (102.9, 161.1)	76.3 (42.0, 125.0)	26.2 (20.5, 33.4)	12.4 (10.1, 15.5)	46.9 (15.4, 89.6)	889.2 (667.3, 1156.6)	356.6 (276.5, 455.5)	47.7 (13.4, 97.1)
Eastern Sub‐Saharan Africa	0.3 (0.2, 0.3)	116.3 (101.5, 132.6)	54.6 (26.1, 92.1)	21.2 (18.2, 24.9)	11.9 (10.3, 13.8)	29.9 (5.6, 62.9)	742.6 (622.6, 889.2)	335.9 (286.4, 392.2)	25.2 (−2.8, 63.0)
Central Sub‐Saharan Africa	0.1 (0.1, 0.1)	105.2 (83.2, 131.8)	53.5 (16.5, 98.3)	6.4 (4.7, 8.4)	10.6 (8.1, 13.8)	36.8 (−0.1, 85.3)	225.2 (167.0, 297.3)	309.5 (231.8, 406.9)	31.3 (−6.2, 82.7)
Southern Sub‐Saharan Africa	0.1 (0.1, 0.1)	177.5 (162.9, 193.2)	58.8 (38.3, 85.6)	8.5 (7.7, 9.4)	14.9 (13.6, 16.3)	50 (26.2, 85.9)	271.3 (242.4, 302.8)	412.6 (370.9, 457.5)	44.0 (20.3, 75.9)

### 3.2 Regional Level

In 2021, high‐income North America (543.6), Western Europe (476.5), and Australasia (465.3) had the highest age‐standardized point prevalences for BC (per 100,000 population), whereas South Asia (99.1), Central Sub‐Saharan Africa (105.2), and Eastern Sub‐Saharan Africa (116.3) had the lowest (Table 1). Southern Sub‐Saharan Africa (14.9), Western Sub‐Saharan Africa (12.4), and Eastern Sub‐Saharan Africa (11.9) had the highest age‐standardized death rates from BC in 2021, with the lowest rates in East Asia (4.5), high‐income Asia Pacific (4.9), and North Africa and the Middle East (6.1) (Table 1). In 2021, Southern Sub‐Saharan Africa (412.6), Western Sub‐Saharan Africa (356.6), and Oceania (352.8) had the highest age‐standardized DALY rates (per 100,000 population). In contrast, East Asia (148.7), high‐income Asia Pacific (163.1), and North Africa and the Middle East (194.9) had the lowest (Table 1). Supporting Information S1: Figures S1–S3 show the age‐standardized point prevalence, death, and DALY rates of BC, respectively, by sex in 2021 for all regions in the GBD study. From 1990 to 2021, the largest increases in the age‐standardized point prevalence of BC were observed in North Africa and Middle East (143.7%), East Asia (107.5%), Central Latin America (96.9%), and South Asia (96.9%), while the greatest decreases occurred in high‐income North America (−20.1%) and Central Asia (−10.4%) (Table 1). In the same period, nearly half of all regions exhibited declines in age‐standardized mortality rates for BC, with the largest reductions in Australasia (−42.5%), high‐income North America (−41.2%), and Western Europe (−40.4%) (Table 1). In contrast, North Africa and the Middle East (62.8%), South Asia (50.5%), and Southern Sub‐Saharan Africa (50.0%) showed the greatest increases (Table 1). From 1990 to 2021, the age‐standardized DALY rates for BC increased most substantially in North Africa and Middle East (57.0%), Western Sub‐Saharan Africa (47.7%), and South Asia (46.6%), while the largest decreases were seen in high‐income North America (−43.0%), Australasia (−42.1%), and Western Europe (−41.9%) (Table 1). Supporting Information S1: Figures S4–S6 illustrate the percentage changes, from 1990 to 2021, in age‐standardized point prevalence, death, and DALY rates for BC, stratified by sex.

The number of prevalent cases of BC increased from 8.76 million in 1990 to 20.62 million in 2021. Western Europe, high‐income North America, and East Asia had the highest number of prevalent cases in 1990, and these same regions had the largest numbers in 2021 (Supporting Information S1: Table S2). The number of deaths caused by BC increased from 355,132 in 1990 to 673,560 in 2021, with South Asia, East Asia, and Western Europe having the highest numbers of deaths in 2021 (Supporting Information S1: Table S3). The number of DALYs due to BC increased from 11.16 million in 1990 to 20.59 million in 2021, with South Asia, East Asia, and Southeast Asia having the highest numbers of DALYs in 2021 (Supporting Information S1: Table S4).

### 3.3 National Level

In 2021, the national age‐standardized point prevalence of BC ranged from 55.3 to 886.3 cases per 100,000. Monaco (886.3), France (582.4), and the United States (556.0) had the highest age‐standardized point prevalences of BC, whereas Mongolia (55.3), Chad (57.2), and Gambia (57.7) had the lowest estimates (Supporting Information S1: Table S2). The national age‐standardized death rate of BC in 2021 varied from 2.2 to 21.9 deaths per 100,000. The highest rates were observed in Palau (21.9), Fiji (21.1), and Nauru (21.0), while the lowest rates were found in Oman (2.2), Mongolia (3.3), and Bangladesh (3.7) (Supporting Information S1: Table S3). In 2021, the national age‐standardized DALY rate of BC ranged from 61.6 to 640.8 per 100,000. The highest rates were recorded in Nauru (640.8), the Bahamas (626.9), and American Samoa (609.9), whereas the lowest rates were reported in Oman (61.6), Mongolia (101.9), and the Maldives (108.5) (Supporting Information S1: Table S4).

**Figure 1:**
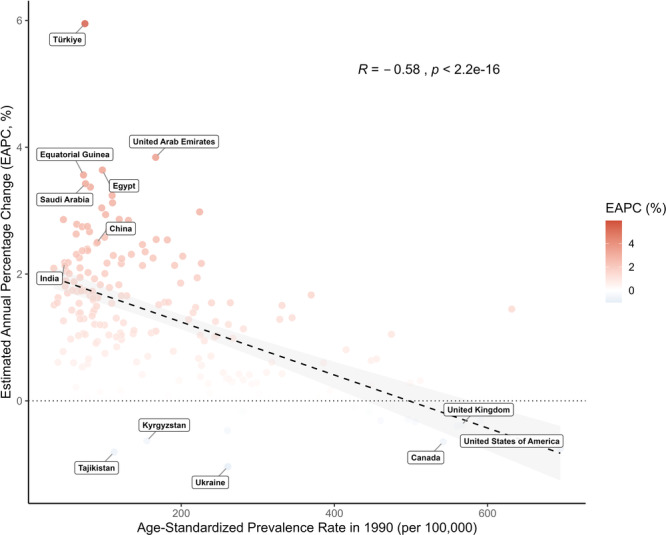
Correlation between age‐standardized prevalence rate in 1990 and the estimated annual percentage change from 1990 to 2021 (generated from data available at https://ghdx.healthdata.org/gbd-results-tool).

To analyze the global shift in disease dynamics, we correlated the age‐standardized prevalence rate from the 1990 baseline with the EAPC through 2021. A significant negative correlation was observed (*R* = −0.58, *p* < 2.2e−16) (Figure 1). Specifically, nations that started with the lowest prevalence in 1990 recorded the most aggressive annual growth, with EAPCs exceeding 4%. In contrast, countries with the highest initial burden exhibited the lowest or even negative EAPC values. Over the same period, Türkiye (130.1%), Egypt (101.1%), and Lesotho (97.7%) showed the greatest increases in age‐standardized death rate, whereas the largest decreases were observed in Denmark (−51.5%), Luxembourg (−49.8%), and Ireland (−49.4%) (Supporting Information S1: Table S3). Between 1990 and 2021, Türkiye (122.6%), Zimbabwe (105.0%), and Lesotho (98.5%) experienced the most pronounced rises in age‐standardized DALY rates for BC. By comparison, the steepest reductions during the study period were documented in Denmark (−57.3%), Luxembourg (−52.5%), and the United Kingdom (−50.4%) (Supporting Information S1: Table S4).

### 3.4 Age and Sex Pattern

**Figure 2:**
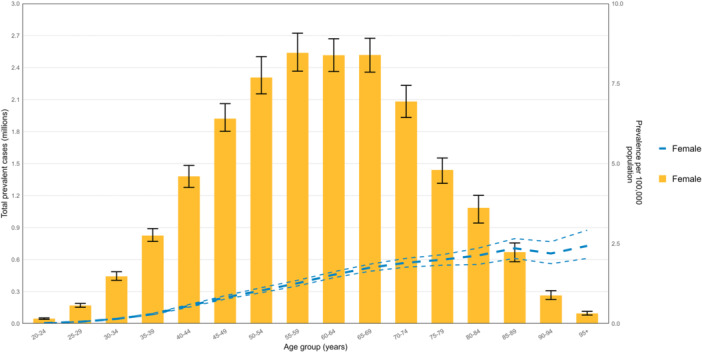
Global female prevalence cases and prevalence of breast cancer per 100,000 population in 2021, by age. Lines indicate prevalent cases with 95% uncertainty intervals for females (generated from data available at https://ghdx.healthdata.org/gbd-results-tool).

**Figure 3:**
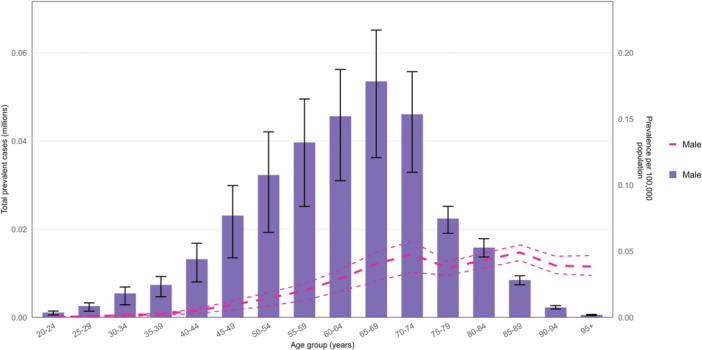
Global male prevalence cases and prevalence of breast cancer per 100,000 population in 2021, by age. Lines indicate prevalent cases with 95% uncertainty intervals for males (generated from data available at https://ghdx.healthdata.org/gbd-results-tool).

In 2021, the global female prevalence of BC began to increase in the 25–29 age group, peaked in the 85–89 age group, and subsequently exhibited fluctuations, exhibiting a bimodal pattern. Similarly, the number of prevalent cases was highest in the 55–59 age group but showed minimal variation thereafter, reflecting a triple‐peak pattern (Figure 2). In terms of sex disparities, male BC prevalence remained markedly lower than female rates across all ages and mostly concentrated in the 65–69 age group (Figure 3). Over the same period, the BC death rate reached its highest level in the oldest age group (≥ 95 years), with female death rates far exceeding male rates in all age groups. The number of deaths among women peaked in the 55–59 age group and then gradually declined with advancing age, whereas among men, deaths were comparatively higher in the 65–69 age group (Supporting Information S1: Figure S7). For women, the DALY rate rose rapidly before the 50–54 age group, peaked in the 55–59 age group, and then declined steadily until the 75–79 age group, after which it increased again. Notably, the DALY rate reached its highest level in women aged ≥ 95 years (Supporting Information S1: Figure S8).

### 3.5 Association With the SDI

**Figure 4:**
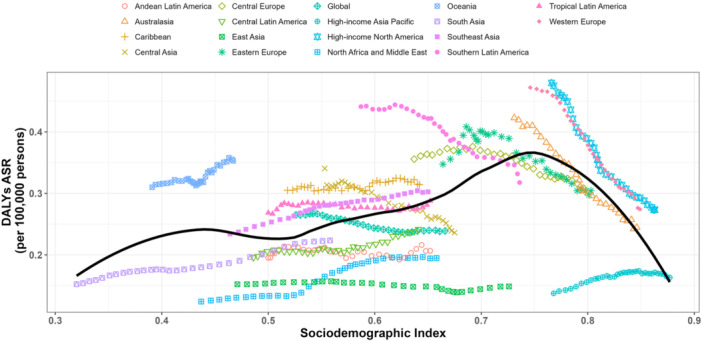
Age‐standardized disability‐adjusted life year (DALY) rates for breast cancer in 21 global burden‐of‐disease regions by SDI, 1990–2021. Thirty‐two points are plotted for each region, showing the age‐standardized DALY rate observed in that region from 1990 to 2021. Expected values for SDI and disease rates based on all locations are shown as solid lines. Regions above the solid line indicate a higher burden than expected (e.g., Oceania), while regions below the solid line indicate a lower burden than expected (e.g., South Asia) (based on https://ghdx.healthdata.org/gbd-results-tool).

**Figure 5:**
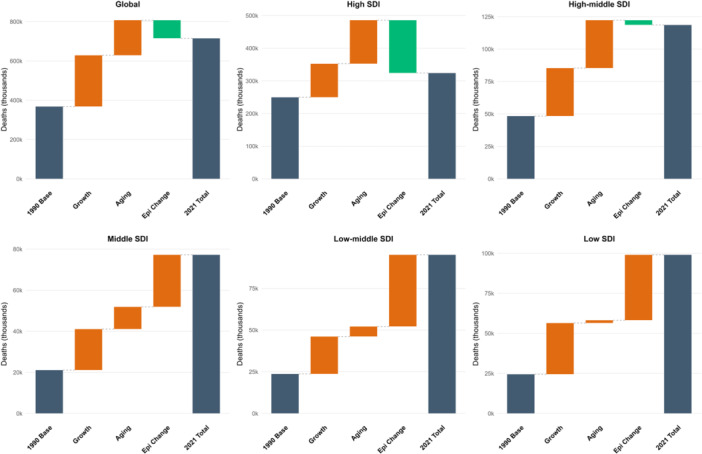
Attribution of changes in absolute breast cancer deaths to demographic and epidemiological drivers (1990–2021) (based on https://ghdx.healthdata.org/gbd-results-tool).

At the regional level, we observed an inverted U‐shaped association between the SDI and the age‐standardized DALY rate of BC from 1990 to 2021 (Figure 4). The age‐standardized DALY rate exhibited a progressive increase with rising SDI values, peaking at approximately 0.75 before entering a sustained decline. Between 1990 and 2021, Oceania, the Caribbean, high‐income North America, and Western Europe displayed DALY rates exceeding levels predicted by their SDI. In contrast, South Asia, Central Latin America, North Africa and the Middle East, Andean Latin America, and East Asia demonstrated lower‐than‐expected disease burdens during this period (Figure 4). At the country level, in 2021, the burden of BC exhibited an initial increase with advancing socioeconomic development, reaching a peak at an SDI of approximately 0.7, followed by a sustained decline. Countries and territories such as Nauru, the Kingdom of Tonga, Fiji, the Bahamas, and American Samoa showed substantially higher than expected burdens, whereas Oman, the Maldives, Mongolia, Algeria, and China demonstrated markedly lower than expected burdens relative to their SDI values (Supporting Information S1: Figure S9). To decipher the structural drivers of mortality across different development stages, we decomposed the change in absolute deaths into population growth, aging, and epidemiological shifts (Figure 5). Globally, the sheer expansion and aging of the population acted as the primary engines of rising mortality. The critical divide, however, lies in the role of epidemiological change (Epi Change). In high‐SDI regions, robust Epi Change acted as a primary negative driver, significantly mitigating the burden imposed by an aging population. Conversely, in low‐ and low‐middle‐SDI regions, the Epi Change actually acted as the primary engine of rising mortality (Figure 5).

### 3.6 Global Health Inequity

**Figure 6:**
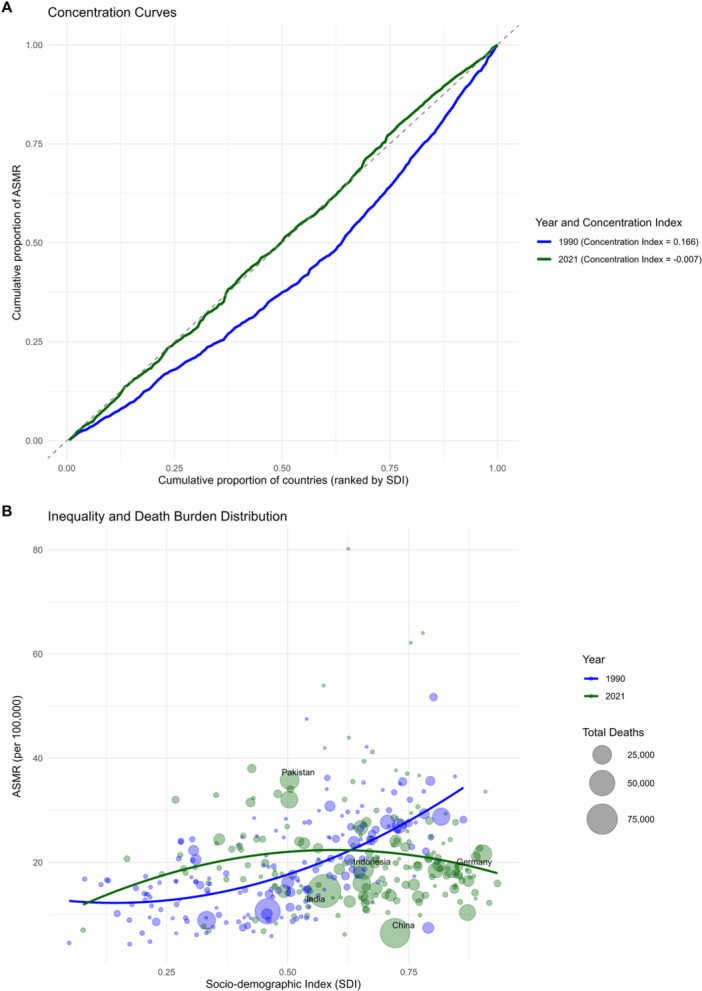
Temporal shifts in global breast cancer mortality inequity from 1990 to 2021. (A) Concentration curves: The *x*‐axis represents the cumulative proportion of countries ranked by SDI, and the *y*‐axis represents the cumulative proportion of age‐standardized mortality rates (ASMR). (B) Inequality and Death Burden Distribution: each bubble represents a country, with the size corresponding to the total number of deaths.

Inequality analysis, as well as concentration curves, further confirmed a systemic shift in the global burden, with mortality becoming more equitably distributed across the socioeconomic spectrum over the three‐decade period. In 1990, the burden was disproportionately concentrated in higher‐SDI regions (Concentration Index = 0.166). In contrast, by 2021, the curve had approximated the line of equality (Concentration Index = −0.007), indicating that the inequality had largely dissipated (Figure 6A). We then compared changes across countries by mapping the age‐standardized mortality rate of each nation against its SDI. We identified a fundamental realignment of the disease epicenter over the past three decades. In 1990, the global landscape was characterized by a positive correlation between development and mortality, where the burden was disproportionately concentrated in high‐SDI nations. However, by 2021, this relationship had inverted into a non‐linear trajectory, with mortality rates now peaking in middle‐SDI regions before entering a sustained decline at the higher end of the spectrum (Figure 6B).

### 3.7 Risk Factors

**Figure 7:**
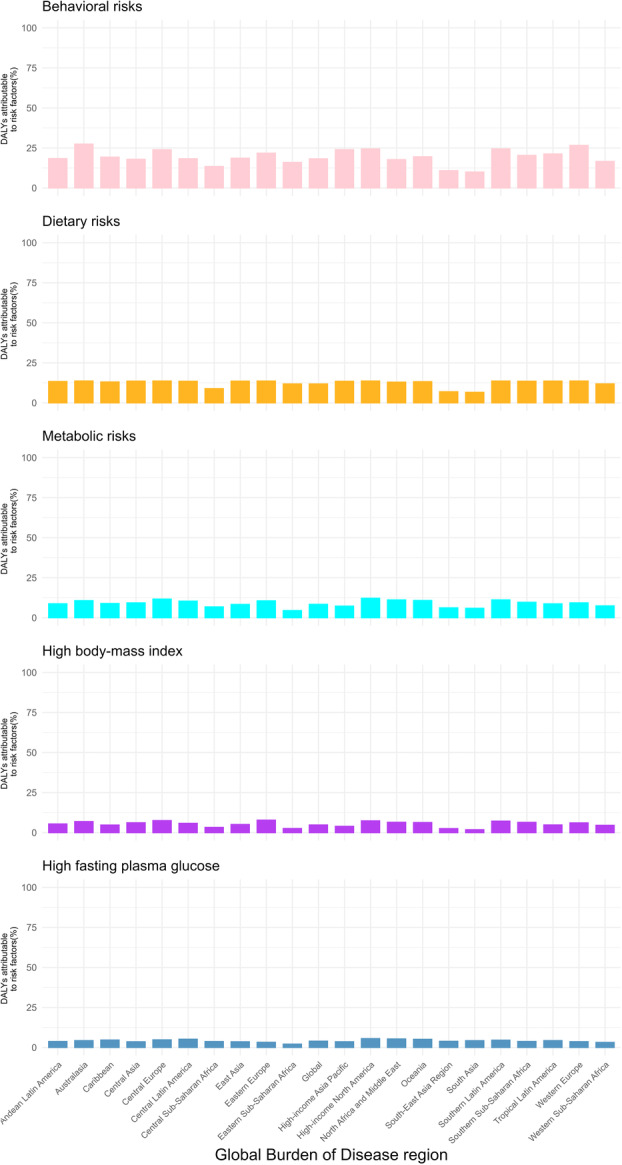
Percentage of disability‐adjusted life years (DALYs) due to breast cancer disease per risk factor in 21 Global Burden of Disease Regions in 2021 (generated from https://ghdx.healthdata.org/gbd-results-tool data).

The proportion of DALYs attributable to BC linked to individual risk factors varies across the GBD regions. Globally, behavioral risks, dietary factors, metabolic risks, high BMI, and elevated fasting plasma glucose collectively accounted for the highest contributions to BC‐related DALYs (Figure 7).

The number of DALYs due to BC that were attributable to individual risk factors also varied across female age groups. Among females, the number of DALYs attributable to the majority of risk factors increased with age until the 55–59 age group, after which a decline was observed. Furthermore, these risk factors were associated with significantly higher attributable DALYs in females compared to males, showing a pronounced gender disparity (Supporting Information S1: Figure S10). Notably, among males, the number of DALYs attributable to BMI increased with age through the 45–49 age group (Supporting Information S1: Figure S12). The number of DALYs due to BC that were attributable to individual risk factors also varied by sex. Among females, DALYs attributable to BMI peak in the 50–59 age group. Additionally, excessive red meat consumption was associated with elevated DALYs in the 45–54 age group (Supporting Information S1: Figure S12). Risk factors, including passive smoking and alcohol use, showed progressive increases in BC‐related DALYs with advancing age, reaching peak levels in the 50–64 age group (Supporting Information S1: Figure S12). Notably, active smoking and secondhand smoke exposure contributed minimally to female BC DALYs, showing no significant age‐specific trends (Supporting Information S1: Figure S12). Among males, BC‐related DALYs were substantially lower compared to females but demonstrated distinct risk patterns, with metabolic risk factors and high BMI acting as primary drivers, both of which decreased with age (Supporting Information S1: Figure S12).

## 4 Discussion

### 4.1 Principal Findings

In this study, based on data from the GBD 2021 study, we present updated estimates of the prevalence, deaths, and DALY counts of BC from 1990 to 2021, along with age‐standardized rates across 204 countries and territories. Globally, BC accounted for 20.6 million prevalent cases, 670,000 deaths, and 20.59 million DALYs in 2021. While the age‐standardized point prevalence rate of BC has increased over the past three decades, the age‐standardized death and DALY rates have declined during the same period. This divergence likely reflects the synergistic effects of population aging, prolonged survival due to therapeutic advances, and secular trends in risk factor exposure. Our decomposition analysis identifies that in resource‐limited settings, the “triple threat“ of aging, population growth, and deteriorating epidemiological control is outpacing current interventions. Furthermore, the distinct metabolic etiology we identified in male BC underscores the need for gender‐specific prevention frameworks (Supporting Information S1: Figures S11 and S12).

### 4.2 Comparison With Similar Studies

A 2019 study reported a 123% increase in the global incidence of BC between 1990 and 2017, alongside declines in deaths and DALY rates. In 2017, the age‐standardized DALY rate for BC was 261.3 per 100,000 population globally [16]. The variance between these earlier figures and our current estimates is best understood as a result of the comprehensive methodological overhaul in the GBD 2021 framework, rather than a conflict in the data itself. Specifically, in this study, we incorporated data from 204 rather than 195 countries. Concurrently, the GBD 2021 framework re‐estimated the entire historical time series by utilizing updated systematic reviews to recalibrate risk factor algorithms. These updates inevitably shift the calculation baselines to improve precision, meaning that absolute values cannot be directly compared across different GBD versions. Importantly, however, the directional trends remain consistent. For instance, A recent investigation examining the impact of tobacco use on the BC burden revealed a significant rise in deaths and DALY counts, despite the lack of reported prevalence estimates. Notably, the study documented a declining percentage trend over the past three decades, consistent with the findings of the present analysis [19].

Epidemiological data indicate that the prevalence of female BC was 1.15% in Italy [20], 0.63% in Australia, and 0.75% in the United States [21]. Crucially, this substantial discrepancy is driven by the comprehensive global coverage of our study. While previous studies largely focused on high‐income nations with established screening programs and older population structures, our analysis incorporates data from populous low‐ and middle‐income regions (e.g., South Asia and Africa). In these regions, age‐standardized prevalence is remarkably lower due to younger demographics and underdiagnosis. Notably, when we stratified our data by region, the prevalence in high‐SDI regions (e.g., high‐income North America: 543.6 per 100,000, or 0.54%) aligned much more closely with the previously reported national estimates. This validates that the observed global difference reflects genuine geographic heterogeneity in disease burden [22].

The downward trajectory of global age‐standardized death and DALY rates suggests a broad success in translating medical innovation into clinical practice, yet these averages mask a profound structural shift in the global geography of the disease. Traditionally, BC has been characterized as a “disease of affluence,” with incidence and mortality disproportionately concentrated in high‐SDI nations due to westernized lifestyles and older population structures [23, 24]. Our current findings using the GBD 2021 framework document a near‐complete “equalization” of global mortality (Concentration Index = −0.007) (Figure 6A). This redistribution of the global burden is further substantiated by the significant negative correlation between the baseline age‐standardized prevalence rate in 1990 and the EAPC from 1990 to 2021 (*R* = −0.58, *p* < 2.2e−16) (Figure 1). This “catch‐up” effect reveals that nations starting with the lowest prevalence in 1990 have experienced the most aggressive increases over the past three decades. For instance, countries such as Türkiye and Equatorial Guinea have seen EAPCs exceeding 4%, whereas historically high‐burden nations like the United States and the United Kingdom are now positioned at the opposite end of the spectrum with stable or even declining EAPCs. Although the prevalence trends show divergence, the mortality rates tend to converge. Our decomposition analysis may explain the mechanism behind this shift (Figure 5). In high‐SDI regions, although population growth and aging put upward pressure on death counts, these are successfully offset by favorable epidemiological changes, reflecting the impact of robust screening and advanced therapeutic access. Conversely, in low‐ and middle‐SDI regions, the epidemiological change itself acts as a primary engine of rising mortality. In these settings, a “triple threat” of rapid population growth, accelerating aging, and deteriorating epidemiological control, often characterized by delayed diagnosis and restricted treatment pathways, collectively drives the sharp increase in absolute mortality. This “equalization” of mortality in 2021, while appearing balanced on a graph, actually signals a deepening crisis of inequity. As mortality rates in countries like Pakistan now exceed those in nations like Germany, the resources to manage the disease remain concentrated in the West. This mirrors World Health Organization reports indicating that pathology service coverage is nearly 95% in high‐income countries but less than 30% in low‐income nations [25]. While high‐SDI countries have successfully utilized medical innovation to lower death rates despite high prevalence, lower‐SDI regions face a “prevalence‐mortality gap” where they bear a disproportionate share of deaths relative to their known cases.

The highest age‐standardized point prevalence in 2021 was observed in Monaco, with 886.3 cases per 100,000 population. A cited study used epidemiological data to assess the burden of BC attributable to modifiable risk factors across European regions. The authors identified alcohol consumption as the primary preventable risk factor for the burden of BC in Europe, accounting for over 30% of cases in Western Europe [26]. During the same period, Palau exhibited the highest age‐standardized death rate, with 21.9 deaths per 100,000 population. The elevated BC deaths in Palau may be attributed to declining screening coverage, late‐stage diagnoses, inadequate localized treatment capacity, restricted referral pathways, and the absence of cancer survivorship support programs, all of which collectively contribute to poor patient prognosis. These death patterns were associated with systemic deficiencies in implementing comprehensive cancer control policies and an underdeveloped healthcare infrastructure [27].

We identified poor concordance between BC prevalence and death rates ranked by region. For example, although high‐income North America exhibited the highest prevalence (543.6 per 100,000), it ranked tenth in death rates. This discrepancy is likely attributable to improved screening coverage and advanced medical technology. Conversely, Southern Sub‐Saharan Africa demonstrated the highest death rate (14.9 per 100,000) despite ranking thirteenth in prevalence. This pattern might be explained by deficient screening systems, inadequate treatment resources, and lower life expectancy, reducing prevalent case accumulation [28], collectively contributing to reduced survival outcomes. These findings were particularly evident in low‐income countries, where socioeconomic resource constraints, limited educational attainment, and poor healthcare accessibility constitute critical determinants of significantly reduced survival rates among BC patients [29]. The World Health Organization reports substantial disparities in global cancer pathology service coverage: less than 30% in low‐income countries, 55% in lower‐middle‐income countries, and 95% in high‐income nations [30]. Furthermore, healthcare systems in these countries prioritize acute infectious disease management over chronic conditions, necessitating governmental reinforcement of basic diagnostic resource allocation in under‐resourced primary healthcare facilities.

The burden of BC peaks in postmenopausal women [31]. Physiologically, age‐related estrogen fluctuations, diminished breast tissue repair capacity, and cumulative genetic mutations may collectively contribute to elevated tumor aggressiveness and mortality risk [32, 33, 34]. Moreover, BC is frequently associated with metabolic syndrome, cardiovascular comorbidities, and reduced bone density—age‐related physiological declines that synergistically exacerbate treatment complexity and mortality rates in middle‐aged and elderly patients. This study found that women aged 50+ years accounted for the highest proportion of BC‐related deaths, aligning with global cancer registry trends [35]. Notably, lifestyle factors prevalent in this age group—including rising obesity rates and reduced physical activity—demonstrate dose–response relationships with poor BC prognosis [36]. This age‐specific risk pattern suggests heightened biological susceptibility to endocrine‐disrupting agents and metabolic dysregulation in older female populations.

In contrast to this female‐predominant landscape, the gender disparity in BC extends beyond simple incidence statistics. While the lower disease burden in males is fundamentally anchored in differential hormonal milieus and anatomical distinctiveness, the distinct epidemiological patterns we observed point to deeper divergences. Clinically, the age‐specific prevalence trends offer a compelling narrative: the distinct peak observed in females (55–59 years) largely captures the interception of asymptomatic cases via mammography. The absence of such screening for men results in a markedly different, linear accumulation of cases driven almost exclusively by symptomatic presentation.

Beneath these clinical patterns lies a distinct etiological profile. Unlike the reproductive‐driven risk matrix in females, our analysis suggests that male BC is predominantly fueled by metabolic dysregulation, particularly elevated BMI (Supporting Information S1: Figure S12). This aligns with mechanistic evidence that obesity‐induced aromatization of androgens to estrogens in adipose tissue serves as a critical carcinogenic driver in the male breast, highlighting a need for prevention strategies that prioritize metabolic health in this population.

Despite its rarity, male BC remains a clinically neglected entity fraught with specific challenges. Evidence indicates that male carriers of pathogenic BRCA1/2 mutations exhibit lifetime BC risks of 2%–6% and 7%–13%, respectively [37]. In particular, male patients predominantly present with advanced‐stage disease and demonstrate poorer prognoses compared to their female counterparts [38]. Notably, a SEER database‐based cohort analysis revealed that despite significantly higher ER positivity rates in males (92.4% vs. 77.5% in females), no significant difference in 5‐year survival rates was observed. Furthermore, age‐period‐cohort modeling identified significantly steeper annual decline rates in female mortality compared with those in males, suggesting systemic delays in implementing BC therapeutic interventions for male populations [39].

This study identified behavioral risks (alcohol use, physical inactivity), metabolic risks (high body mass index, elevated fasting glucose), and dietary risks (red meat consumption) as major contributors to the BC burden (Figure 7). Since these individually attributable risk factors are largely preventable, as noted by Colditz and Bohlke, it is estimated that over half of BC cases could be avoided through healthy lifestyle choices and interventions [40]. Addressing this public health challenge, therefore, requires both personal commitment and coordinated global action. Several international initiatives have been implemented to mitigate these risks, most notably the WHO's 2017 Global BC Initiative framework, which emphasizes public health education to enhance awareness, breastfeeding promotion, dietary optimization, and alcohol moderation, as well as the development of early detection systems and healthcare system optimization [41]. Since the late 20th century, institutionalized national screening programs have been established in high‐income countries, including the United States, the United Kingdom, Australia, and Canada, though implementation remains limited to pilot projects in countries such as Mexico, Vietnam, and South Africa [42, 43].

The relationship between BC‐related DALYs and the SDI demonstrated nonlinear patterns at the national level. Our analysis revealed a positive correlation between the BC burden and national development levels, peaking at upper‐middle SDI scores before declining in high‐SDI countries. WHO reports indicate that while high‐income countries exhibit higher BC incidence rates, approximately 70% of BC deaths occur in low‐ and middle‐income countries. Furthermore, findings from the GBD 2021 study show an increased BC burden among women of reproductive age across all SDI tiers, except in high‐SDI regions, from 1990 to 2021 [44]. Projections estimate that low‐ and lower‐middle‐income SDI countries will bear the greatest BC burden over the next decade [45]. Notably, environmental risk exposures demonstrate differential associations with BC burden by development status. Alcohol consumption emerges as the predominant risk factor for BC death in most countries [16], while secondhand smoke exposure constitutes a significant risk factor in low‐ and middle‐SDI countries [46]. Smoking prevalence shows declining trends in both high‐ and middle‐SDI countries, with more substantial reductions observed in high‐SDI nations [47]. Concurrently, exposure to ambient particulate matter is increasing in lower‐SDI countries while decreasing in higher‐SDI counterparts [48]. The SDI serves as a comprehensive proxy for socioeconomic disadvantage. Low‐SDI countries typically face systemic healthcare disparities, including limited availability and affordability of essential BC services such as early screening, diagnostic confirmation, and evidence‐based treatments [48]. These structural deficiencies result in delayed clinical presentations, advanced‐stage diagnoses, and consequently elevated disease burden with heightened premature mortality risks among BC patients [49].

Ultimately, strategic focus should prioritize establishing equitable healthcare resource allocation mechanisms in regions with the heaviest BC burden by intensifying screening networks and improving treatment accessibility to reverse survival disparities. These efforts should concurrently address systemic underinvestment in prevention and control measures relative to other major chronic diseases in low‐ and middle‐income countries, where BC persists as the leading threat to women's health [50]. Moreover, substantial heterogeneity in BC clinical presentations across national populations necessitates the accelerated development of population‐specific disease surveillance systems in high‐incidence regions [51]. In resource‐constrained settings where universal imaging technology access cannot be guaranteed, implementing standardized triage pathways through the integration of clinical sign recognition with referral stratification is essential to enable early intervention breakthroughs.

### 4.3 Strengths and Limitations of This Study

Building on prior GBD 2019 analyses, this study offers an update by capitalizing on the GBD 2021 dataset, which uniquely captures the epidemiological shifts during the COVID‐19 pandemic. Beyond merely extending the time frame, we provided a more nuanced spatiotemporal assessment; specifically, by correlating baseline burdens with EAPC, we identified a distinct “catch‐up” trend in LMICs that simple prevalence rates might overlook. Our approach also deepens the understanding of risk attribution through the causality risk assessment framework, shifting the focus from incidence to DALYs to better inform prevention strategies for modifiable risks. Together with our granular age‐stratified analysis addressing the rising concern of early‐onset BC, these improvements offer a more dynamic and actionable evidence base for global health policy. However, several limitations should be acknowledged. First, the scarcity of high‐quality epidemiological data constrained the accuracy of BC burden estimation. Second, while established risk factors were incorporated, the absence of genetic predisposition data (e.g., BRCA mutations) and emerging environmental endocrine disruptor exposure metrics limited the comprehensiveness of the attributable risk analysis. Third, the integration of clinical records may introduce bias, as data validity critically depends on rigorous cleaning processes and stage‐specific correction protocols. Underdiagnosis of BC often stems from patients neglecting breast masses or delaying care‐seeking due to cultural taboos, compounded by limitations in imaging equipment and inadequate lesion recognition skills among primary care physicians, which collectively exacerbate screening gaps [52]. Fourth, in countries lacking robust cancer registries, BC mortality estimates rely on community surveys or modeled projections. Since previous studies failed to stratify BC deaths by pathological subtypes or molecular classifications, critical information remains excluded from mortality statistics, potentially leading to underestimation [52]. Regarding the potential direction of bias resulting from these limitations, the gaps in data quality and registration systems—particularly in low‐SDI regions—suggest that our reported DALY rates and case numbers likely underestimate the true global burden due to under‐diagnosis. Similarly, since our analysis is restricted to currently quantifiable risk factors, the total attributable burden presented here should be viewed as a conservative estimate. On the other hand, regarding temporal trends, the rapid rise in incidence observed in high‐SDI regions might be partially inflated by detection bias, where improved screening programs identify cases that would have previously gone unrecorded, rather than reflecting a purely biological increase in disease frequency. This underscores the imperative to establish molecularly characterized standardized surveillance systems and strengthen regional tumor registry networks, which would significantly enhance mortality data resolution and inform precision therapeutic resource allocation. Furthermore, it is critical to acknowledge that the GBD estimates are subject to the underlying assumptions and prior distributions of the modeling framework (e.g., DisMod‐MR 2.1). In geographical regions with sparse or low‐quality primary data, the model relies heavily on covariate‐driven estimations and spatio‐temporal smoothing. Consequently, these areas exhibit higher uncertainty. Readers are strongly cautioned against over‐interpreting point estimates in such contexts; instead, the provided 95% UIs should be meticulously considered as they more accurately reflect the range of potential values and the reliability of the estimates. Additionally, this study is based on the GBD 2021 dataset, which was the most recent publicly available version at the time of our analysis. Although the GBD 2023 update has recently been initiated, its full dataset is currently restricted to senior collaborative members and is not yet available for independent research. We acknowledge this as a limitation and suggest that future studies should utilize the 2023 data once fully released to further track the evolving global burden of BC.

## 5 Conclusions and Policy Implications

BC constitutes a major global public health challenge, characterized by escalating healthcare expenditures and growing socioeconomic burdens. While age‐standardized death rates have decreased in certain high‐income regions through enhanced screening implementation, global incidence counts continue to demonstrate a sustained upward trend. Demographic aging and lifestyle transitions are projected to further amplify this disease burden, particularly in resource‐limited settings. Systematic analysis of BC epidemiological patterns and risk factor attribution provides critical evidence for accurately forecasting disease trajectories, optimizing multilevel screening frameworks, and guiding precision‐driven therapeutic resource allocation. This evidence base should inform policymakers in addressing anticipated surges in prevention demand and mitigating persistent health inequities.

## Author Contributions

**Jiahui Huang:** conceptualization (equal), writing – original draft (equal), writing – review and editing (equal), software (equal), data curation (equal), formal analysis (equal). **Jiajin Li:** writing – review and editing (equal), writing – original draft (equal), software (equal), formal analysis (equal), data curation (equal). **Shengbin Pei:** writing – original draft (equal), writing – review and editing (equal), software (equal), formal analysis (equal), data curation (equal). **Cheng Zeng:** software (equal), formal analysis (equal), data curation (equal), writing – original draft (equal). **Jiangdong Jin:** software (supporting), formal analysis (supporting), data curation (supporting), writing – original draft (supporting). **Xun Hu:** software (supporting), data curation (supporting). **Jin Shi:** software (supporting), data curation (supporting), formal analysis (supporting), writing – original draft (supporting). **Wei Dong:** software (supporting), formal analysis (supporting), data curation (supporting), writing – original draft (supporting). **Ruimeng Wang:** writing – original draft (supporting), software (supporting), formal analysis (supporting), data curation (supporting). **Yiqin Xia:** data curation (equal), funding acquisition (equal), formal analysis (equal), validation (equal), visualization (equal), conceptualization (equal), investigation (equal). **Jiani Wang:** conceptualization (equal), investigation (equal), funding acquisition (equal), visualization (equal), validation (equal), software (equal), data curation (equal).

## Ethics Statement

The authors have nothing to report.

## Consent

The authors have nothing to report.

## Conflicts of Interest

The authors declare no conflicts of interest.

## Supporting information


**Figure S1:** Age−standardized point prevalence of breast cancer by sex for all regions in the Global Burden of Disease Study, 2021. **Figure S2:** Age−standardized point mortality rates for breast cancer by sex for all regions in the Global Burden of Disease Study, 2021. **Figure S3:** Age−standardized point DALY rates for breast cancer by sex for all regions in the Global Burden of Disease Study, 2021. **Figure S4:** Percentage change in age−standardized point prevalence of breast cancer by sex from 1990 to 2021. **Figure S5:** Percentage Change in Age−Standardized Point Death Rates for Breast Cancer by Sex from 1990 to 2021. **Figure S6:** Percentage Change in Age−Standardized Point DALY Rates for Breast Cancer by Sex, 1990 to 2021. **Figure S7:** Global death of cases and death of breast cancer per 100,000 population in 2021, by age and sex. Lines indicate prevalent cases with 95% uncertainty intervals for males and females (generated from data available at https://ghdx.healthdata.org/gbd-results-tool). **Figure S8:** Global DALYs of cases and DALYs of breast cancer per 100,000 population in 2021, by age and sex. **Figure S9:** Age−standardized DALY rates for breast cancer (by sociodemographic index) for 204 countries and territories in 2021; expected values based on sociodemographic index and incidence rates for all locations are shown as black lines.and disease incidence rates for all sites are shown as black lines. **Figure S10:** Percentage of Disability−Adjusted Life Years (DALYs) attributable to risk factors due to breast cancer among women in 21 GBD areas, 2021. **Figure S11:** Percentage of Disability−Adjusted Life Years (DALYs) attributable to risk factors due to breast cancer among men in 21 GBD areas, 2021. **Figure S12:** Number of Disability−Adjusted Life Years (DALYs) attributable to each risk factor due to breast cancer, 2021. **Table S1:** Search terms used for literature on breast cancer in the Global Burden of Disease 2021 study. **Table S2:** Breast Cancer Prevalence Cases and Age−Standardized Rates (ASRs) per 100,000 Population, 1990 and 2021 Percentage change in age−standardized rates (ASRs) per 100,000 population, by location. **Table S3:** Number of Deaths from Breast Cancer and Percentage Change in Age−Standardized Rates (ASRs) per 100,000 Population, 1990 and 2021 Percentage change in age−standardized rates (ASRs) per 100,000 population, by location. **Table S4:** Percentage Change in Disability−Adjusted Life Years (DALYs) Due to Breast Cancer and Age−Standardized Rates (ASRs) per 100,000 Population, 1990 and 2021 Percentage change in age−standardized rates (ASRs) per 100,000 population, by location.

## Data Availability

The data that support the findings of this study are openly available in GBD at https://www.healthdata.org/research-analysis/gbd.
