# Supplementary material for: The Burden of Breast Cancer and its Attributable Risk Factors in 204 Countries and Territories, 1990–2021: Results From the Global Burden of Disease Study 2021
Source: Cancer Innov. 2026 Apr 10;5(2):e70055. doi: 10.1002/cai2.70055 (PMC13066758; doi:10.1002/cai2.70055)
Supplement: Supplementary file 1 — Figure S1: Age−standardized point prevalence of breast cancer by sex for all regions in the Global Burden of Disease Study, 2021. Figure S2: Age−standardized point mortality rates for breast cancer by sex for all regions in the Global Burden of Disease Study, 2021. Figure S3: Age−standardized point DALY rates for breast cancer by sex for all regions in the Global Burden of Disease Study, 2021. Figure S4: Percentage change in age−standardized point prevalence of breast cancer by sex from 1990 to 2021. Figure S5: Percentage Change in Age−Standardized Point Death Rates for Breast Cancer by Sex from 1990 to 2021. Figure S6: Percentage Change in Age−Standardized Point DALY Rates for Breast Cancer by Sex, 1990 to 2021. Figure S7: Global death of cases and death of breast cancer per 100,000 population in 2021, by age and sex. Lines indicate prevalent cases with 95% uncertainty intervals for males and females (generated from data available at https://ghdx.healthdata.org/gbd-results-tool). Figure S8: Global DALYs of cases and DALYs of breast cancer per 100,000 population in 2021, by age and sex. Figure S9: Age−standardized DALY rates for breast cancer (by sociodemographic index) for 204 countries and territories in 2021; expected values based on sociodemographic index and incidence rates for all locations are shown as black lines.and disease incidence rates for all sites are shown as black lines. Figure S10: Percentage of Disability−Adjusted Life Years (DALYs) attributable to risk factors due to breast cancer among women in 21 GBD areas, 2021. Figure S11: Percentage of Disability−Adjusted Life Years (DALYs) attributable to risk factors due to breast cancer among men in 21 GBD areas, 2021. Figure S12: Number of Disability−Adjusted Life Years (DALYs) attributable to each risk factor due to breast cancer, 2021. Table S1: Search terms used for literature on breast cancer in the Global Burden of Disease 2021 study. Table S2: Breast Cancer Prevalence Cases and Age−Standardized Rat [file CAI2-5-e70055-s001.pdf]

Table S1:Search terms used for literature on breast cancer in the Global Burden of Disease 2021 study
(breast cancer[Title/Abstract] AND prevalence[Title/Abstract] or incidence [Title/Abstract] or mortality [Title/Abstract] or death [Title/Abstract])

Table S2: Breast Cancer Prevalence Cases and Age−Standardized Rates (ASRs) per 100,000 Population, 1990 and 2021<br>Percentage change in age−standardized rates (ASRs) per 100,000 population, by location<br>(Generated from data provided by http://ghdx.healthdata.org/gbd−results−tool)					
	1990		2021		Percentage change in the ASRs per 100,000
	No (95% UI)	ASRs per 100,000 (95% UI)	No (95% UI)	ASRs per 100,000 (95% UI)	
Global	8760184 (8185959,9544334)	218.4 (203,237.8)	20624861 (19544567,21779109)	238.9 (226.2,252.2)	9.3 (0.1,18.2)
High−income North America	2283882 (2134187,2455135)	680.4 (639.5,725.2)	3284777 (3092737,3457261)	543.6 (514.6,570.3)	−20.1 (−24.3,−15.9)
Canada	172586 (158479,187788)	542.1 (500.1,589.4)	285867 (264086,309377)	442 (409.1,477.3)	−18.5 (−27.2,−9)
Greenland	98 (81,118)	260.3 (219.6,305.7)	152 (121,188)	212.1 (170.9,259.8)	−18.5 (−36.8,7)
United States of America	2111145 (1972562,2266286)	695 (653.5,741.5)	2998707 (2813527,3167150)	556 (525.2,584.7)	−20 (−24.3,−15.9)
Australasia	108068 (99095,118887)	471.2 (434.2,516.5)	223865 (205295,241764)	465.3 (427.9,503.6)	−1.3 (−11.6,9.7)
Australia	85692 (78390,94371)	447.6 (411.2,491.1)	185084 (169154,201497)	457.7 (418.6,499.3)	2.3 (−9.3,14.8)
New Zealand	22376 (20444,24793)	590.9 (542.2,652.8)	38781 (35512,41866)	505.2 (464.4,544.6)	−14.5 (−22.7,−5.5)
High−income Asia Pacific	413887 (374258,462151)	201.8 (182.5,225.3)	1151556 (1053179,1232216)	319.4 (296.4,339.6)	58.3 (42.5,73.2)
Brunei Darussalam	231 (181,286)	170.7 (136.5,209.2)	1067 (871,1280)	243.3 (202.1,286.6)	42.6 (8.5,86.6)
Japan	370569 (335564,413957)	220.8 (200.6,245.9)	935843 (849175,1010382)	357.7 (331.7,381.9)	62 (46.8,77.8)
Singapore	6215 (5700,6890)	239.5 (217.3,267.1)	26946 (25095,28797)	311.1 (289.7,332.1)	29.9 (15.8,46.9)
Republic of Korea	36873 (31553,43137)	109.7 (93.2,129.4)	187700 (162182,213887)	218.9 (189.7,250.5)	99.5 (62.1,140.8)
Western Europe	2586999 (2382000,2847455)	482.3 (447.4,526.2)	3817586 (3592248,4000538)	476.5 (453.3,496.5)	−1.2 (−8.9,6.3)
Andorra	245 (189,329)	415.2 (324.9,553.5)	706 (548,892)	476.2 (364,608)	14.7 (−20.5,62.7)
Austria	50251 (45554,55557)	460.4 (421.4,504.8)	60213 (55392,64949)	374.7 (346.9,404)	−18.6 (−27.6,−9.4)
Belgium	79257 (72507,87209)	560.2 (515.6,611.8)	99175 (91360,106051)	482.6 (446.6,517.1)	−13.8 (−22.7,−4.5)
Cyprus	2993 (2535,3540)	369.7 (316,433.4)	10284 (8808,11769)	526.5 (450.1,602.5)	42.4 (15.2,73)
Denmark	37021 (33947,40576)	505.4 (465.7,547.7)	43744 (40693,47087)	414.1 (386.4,445.5)	−18.1 (−25.9,−8.6)
Finland	30422 (27558,33816)	448.1 (407.3,495.2)	50149 (45706,54056)	468.1 (430.1,506.2)	4.5 (−7.8,18.1)
France	364274 (330789,400603)	474.4 (432.9,518.9)	679351 (624022,729152)	582.4 (536.8,626)	22.8 (10.2,37.7)
Germany	545957 (498197,609344)	455 (418.7,502.7)	804808 (743970,855623)	482.1 (448.7,510.9)	6 (−5.8,17.8)
Greece	67702 (61728,75111)	474.1 (436.3,520.5)	94135 (87563,100380)	470.7 (439.6,503.9)	−0.7 (−10.3,10.3)
Iceland	1344 (1212,1483)	496 (448.3,545)	2381 (2166,2595)	450.5 (408.1,492.1)	−9.2 (−20.1,3.2)
Ireland	19141 (17462,21055)	498.7 (456.6,544.2)	35555 (32720,38314)	484 (445,521.1)	−3 (−13.6,8.4)
Israel	19952 (18212,22017)	427.2 (392.3,470)	45848 (42382,49674)	399.7 (370.1,433.8)	−6.4 (−16.7,4.7)
Italy	427745 (390531,472915)	524.5 (481.9,572)	602127 (552333,646221)	497.8 (463.1,532.8)	−5.1 (−12.6,2.7)
Luxembourg	2525 (2294,2794)	484 (441,530)	4157 (3803,4512)	412.1 (377.4,447.4)	−14.9 (−24.4,−4.2)
Malta	1946 (1775,2148)	456.8 (417.9,504.2)	3632 (3325,3996)	440.2 (402.2,484.8)	−3.6 (−14.9,9.4)
Monaco	370 (299,455)	631.6 (511.4,780.3)	672 (551,832)	886.3 (708.7,1127.2)	40.3 (6.2,93.2)
Netherlands	97313 (89259,107637)	512.1 (472.3,563.2)	155771 (144212,166620)	509.7 (475,543.6)	−0.5 (−10.1,10.1)
Norway	23260 (21011,25965)	378.5 (347.8,416.6)	31775 (29425,34271)	353.1 (329.1,379.5)	−6.7 (−14,0.8)
Portugal	49835 (45091,55868)	385.7 (351.6,427.6)	86032 (79471,91799)	432.9 (399.4,462.8)	12.2 (1,24.9)
San Marino	129 (106,154)	394.4 (324,474.4)	207 (157,269)	330.4 (240,444.8)	−16.2 (−42.3,20.2)
Spain	201149 (181671,224623)	399 (364.1,438.5)	319708 (294450,342785)	379.4 (350.5,407.3)	−4.9 (−14.7,5.7)
Sweden	59442 (54028,66647)	447.8 (410,493.2)	72476 (64038,80237)	381.8 (337.2,421.9)	−14.8 (−24.9,−4.5)
Switzerland	43297 (39312,48203)	445.1 (405.6,488.7)	63724 (59040,68304)	385.3 (358.1,410.9)	−13.4 (−22.5,−4.2)
United Kingdom	459303 (424670,500261)	565.7 (528.1,609.1)	547595 (517601,574703)	476.2 (454.1,497)	−15.8 (−20.8,−10.9)
Southern Latin America	111499 (102500,122600)	240.7 (221.6,265.4)	221963 (208149,236120)	264.3 (248.1,280.7)	9.8 (−1.7,21.1)
Uruguay	12223 (11255,13295)	331.7 (307.6,359.6)	19341 (17838,20722)	390.7 (359.8,418.9)	17.8 (5.6,30.7)
Argentina	81959 (74860,90358)	254.8 (233.1,280.6)	152721 (142699,162974)	281.6 (262.1,300.7)	10.5 (−2.6,23.1)
Chile	17312 (15713,19261)	165.8 (150,185.7)	49890 (46171,53709)	202 (186.8,217.2)	21.8 (8.1,36.4)
Eastern Europe	642615 (595818,705375)	235.7 (219.2,258.2)	944483 (861229,1028233)	283.8 (258,310.1)	20.4 (8.2,34.2)
Belarus	29752 (26793,33037)	237.1 (213.1,263.6)	43340 (35838,51834)	286.6 (234.3,346.7)	20.9 (−3,50.3)
Estonia	5593 (5042,6130)	280.9 (254.7,307.8)	7210 (6206,8188)	300.6 (256.6,341.5)	7 (−9.7,25.3)
Latvia	9024 (7952,10098)	259.3 (228,289.1)	10359 (8903,11817)	290.6 (246.1,336.6)	12.1 (−6.8,32.8)
Lithuania	11693 (10723,12830)	266.6 (244.5,291.5)	14943 (13114,16831)	300.4 (261.8,337.8)	12.7 (−2.7,29.5)
Republic of Moldova	10236 (9055,11535)	226.4 (201,254.4)	14786 (12799,17059)	256.5 (221.9,296.9)	13.3 (−5.6,34.6)
Russian Federation	398398 (368424,438269)	224.8 (208.4,246.8)	702840 (636713,768907)	308 (278.4,337.1)	37 (21.8,52.5)
Ukraine	177919 (162414,197726)	260.8 (239.2,289.6)	151006 (112856,201278)	207.8 (152.1,281.9)	−20.3 (−41.8,6.1)
Central Europe	351758 (324390,386795)	238.4 (221.2,261.2)	621333 (581880,661749)	311.9 (291.5,333.1)	30.8 (18.2,44.7)
Albania	2268 (1874,2770)	103.1 (85.3,125.8)	6222 (4882,7792)	159 (124,201.1)	54.3 (12.1,109)
Bosnia and Herzegovina	7107 (6100,8156)	163.2 (141.5,187)	14268 (11805,17006)	247.2 (202.1,297.8)	51.4 (20.1,95.1)
Bulgaria	33421 (29781,37612)	281.2 (250.6,313.5)	47449 (40303,54561)	382 (322.1,446.9)	35.9 (10.2,65.7)
Croatia	19393 (17094,21874)	318.2 (281.1,357.6)	27711 (24345,31017)	353.1 (305.8,399.4)	11 (−5.8,30.2)
Czechia	40149 (35934,44863)	301.2 (270.6,334.4)	60276 (52127,67970)	314.2 (270.1,357.6)	4.3 (−12.4,25.9)
Hungary	41887 (37169,47518)	298.6 (265.4,337)	59724 (52584,67444)	345.2 (300.5,394.8)	15.6 (−0.7,37.2)
Montenegro	2202 (1798,2724)	344.8 (281,424.6)	4083 (3371,4950)	438.9 (361.3,530.9)	27.3 (−2.3,68.4)
North Macedonia	4838 (4077,5603)	244.8 (206.8,282.5)	10485 (8541,12880)	323.1 (263.5,396.8)	32 (6.3,68.9)
Poland	92630 (84658,101857)	215.4 (197.8,235.4)	196831 (176894,215662)	303.8 (272.2,334.5)	41 (23.7,57.3)
Romania	50443 (45621,56604)	183.2 (167,204.5)	89656 (80076,99396)	274.3 (243.4,305.2)	49.7 (25,74.4)
Serbia	29954 (24183,36876)	261.5 (212.2,321)	54861 (44791,67157)	371.3 (300.2,456.8)	42 (5.6,86.7)
Slovakia	14307 (12623,16271)	243.7 (215.2,276.2)	28236 (23216,33384)	311.5 (257.8,368.3)	27.8 (1.5,57.9)
Slovenia	7534 (6785,8410)	311.3 (280.7,347.2)	12487 (10967,14250)	318.6 (276.2,369.5)	2.3 (−14.2,21.4)

**Central Asia**	**86192 (80129,92370)**	**174.4 (161.7,187.7)**	**141123 (128693,154307)**	**156.2 (143.3,169.9)**	**−10.4 (−20.2,−0.1)**
Armenia	8205 (7621,8881)	280.3 (259.4,302.7)	11574 (10419,12883)	270.3 (243.3,301.2)	−3.6 (−15.6,9.3)
Azerbaijan	7958 (6835,8990)	148.9 (128.7,167.2)	18789 (14853,23350)	159.6 (128.1,196)	7.2 (−15.8,32.9)
Georgia	17731 (15829,19475)	288.9 (258.9,315.9)	18053 (16048,20190)	328.5 (291.7,368.8)	13.7 (−3.3,32.8)
Kazakhstan	26608 (23779,29440)	197.9 (177.8,219.2)	33621 (29288,38343)	174.6 (152.8,198.3)	−11.8 (−25.2,5.1)
Kyrgyzstan	4754 (4199,5313)	155.1 (137.5,173.7)	7477 (6290,8790)	134.7 (114.9,157.1)	−13.1 (−27.7,5.5)
Mongolia	510 (418,605)	45.7 (37.8,54.6)	1580 (1311,1886)	55.3 (46.2,65.6)	20.9 (−6.5,52.2)
Tajikistan	3289 (2759,3874)	112.3 (94.2,132.2)	6592 (4457,9359)	88.9 (63.2,122.7)	−20.8 (−46.2,12.1)
Turkmenistan	2433 (2150,2736)	115.3 (101.9,129.7)	6190 (4836,7998)	131.5 (104.7,167)	14 (−11.2,46.1)
Uzbekistan	14703 (13204,16228)	120.5 (107.8,134.2)	37247 (31710,43277)	117.3 (100.9,135.6)	−2.7 (−16.9,15.9)
**Central Latin America**	**123022 (117832,128751)**	**126.4 (120.1,133)**	**653623 (576056,733105)**	**248.9 (219.7,278.9)**	**96.9 (73.6,122.4)**
Colombia	30716 (28280,33223)	149.4 (137.8,161.2)	165299 (139030,197253)	301.1 (253.6,359.6)	101.6 (67,144.3)
Costa Rica	3704 (3421,4038)	191.4 (176.3,208.1)	20958 (18453,23771)	382.2 (336.4,432.3)	99.7 (70.7,129.3)
El Salvador	2611 (2280,2962)	81.4 (71.3,91.7)	13993 (11159,17434)	231.4 (184.3,288.1)	184.4 (116.6,268.8)
Guatemala	1894 (1743,2065)	46.1 (42.2,50.5)	13166 (11210,15449)	108 (92.3,126.5)	134 (97.6,178.9)
Honduras	1578 (1237,1945)	66.9 (51.9,83.1)	9738 (7149,13196)	134.5 (100.1,180.4)	100.9 (43.5,177.8)
Mexico	61202 (58440,64249)	122 (115.9,128.9)	306859 (260152,358986)	226.2 (192.1,263.7)	85.5 (55.8,117.5)
Nicaragua	1298 (1122,1493)	70.7 (61.1,81.4)	8234 (6641,10148)	149.5 (121.7,183.3)	111.4 (69.7,171.1)
Panama	2444 (2237,2655)	149.4 (137.1,162.5)	14069 (11580,16975)	319.6 (263.1,386)	114 (72.3,167.7)
Venezuela (Bolivarian Republic of)	17574 (16398,18876)	152.9 (142.7,163.6)	101306 (78219,128351)	325 (252,410.1)	112.6 (65.1,174.6)
**Andean Latin America**	**18052 (15708,20747)**	**77.9 (67.9,89.2)**	**89175 (71655,109895)**	**143.2 (115.3,176.4)**	**83.8 (48.3,126.9)**
Bolivia (Plurinational State of)	2966 (2051,4113)	80.9 (56.7,111)	13374 (9162,18829)	132.6 (91.7,186.7)	64 (16.4,133.7)
Ecuador	3769 (3489,4082)	62.7 (57.9,68.1)	22519 (17636,27903)	132.7 (104.2,164.3)	111.5 (61.9,168.2)
Peru	11318 (9436,13323)	83.9 (70.4,98.2)	53282 (39567,68618)	151.5 (113.1,195)	80.5 (31.5,147.2)
**Caribbean**	**50340 (47036,53969)**	**186.2 (174.1,199.4)**	**127410 (111435,143695)**	**239.1 (208.8,270.2)**	**28.4 (13.1,45.2)**
Antigua and Barbuda	134 (121,147)	264.1 (237.6,290.3)	426 (395,462)	381 (354.2,411.6)	44.3 (27.6,64)
Barbados	856 (784,931)	331.6 (304,360.1)	2152 (1755,2662)	454.6 (368.3,561.1)	37.1 (9.3,72.1)
Belize	83 (74,91)	85 (76.6,93.6)	467 (412,526)	134.3 (119.2,150.5)	58 (34.7,85.8)
Bermuda	274 (246,304)	425.2 (381.9,471.2)	536 (452,668)	452.4 (377.2,562.9)	6.4 (−12,34.4)
Bahamas	584 (536,639)	328.6 (301.8,358.7)	1937 (1611,2355)	439.9 (367.7,531.7)	33.9 (6.7,64.5)
Cuba	22968 (21296,24736)	222.6 (206.6,239.7)	51107 (44277,59504)	279.6 (243,328)	25.6 (6.9,48.2)
Dominica	131 (114,150)	230.1 (200.6,263.5)	217 (170,266)	262.3 (207.4,321.1)	14 (−12.8,47.3)
Dominican Republic	3814 (3264,4357)	90.6 (77.9,102.9)	14102 (11124,17846)	134.4 (106.9,170)	48.3 (11.9,95.6)
Grenada	142 (128,157)	218.7 (197.3,242.4)	362 (317,412)	308.9 (270.9,350.6)	41.2 (19,65)
Guyana	561 (494,645)	128.8 (113.9,147.3)	1412 (1086,1824)	197.6 (153.4,252.8)	53.5 (13.8,101)
Haiti	3922 (2565,5868)	105.4 (71.4,153.4)	12517 (8069,19196)	137.7 (90.8,208)	30.6 (−10.2,91.3)
Jamaica	3390 (3124,3645)	199.2 (183.5,213.8)	10355 (8069,13281)	337.8 (262.7,433)	69.6 (31,120.1)
Puerto Rico	8826 (8169,9504)	246.7 (228.4,266)	19394 (16552,22566)	337.9 (287.3,395.2)	37 (15.5,65)
Saint Kitts and Nevis	93 (84,102)	275.5 (251,302.2)	210 (173,251)	281.9 (235.7,334.7)	2.3 (−16.6,25.1)
Saint Lucia	208 (193,224)	235.3 (218.5,253.9)	601 (506,718)	251 (211.8,299.6)	6.7 (−11.6,30)
Saint Vincent and the Grenadines	169 (153,184)	238.8 (216.3,261)	393 (345,449)	279.1 (244.4,319.4)	16.9 (0,38)
Suriname	338 (290,389)	121.9 (105.8,139.8)	1029 (816,1285)	158.2 (125.7,197.1)	29.8 (−2.3,67.7)
Trinidad and Tobago	1861 (1713,2018)	209.6 (193.2,227.2)	5449 (4242,6829)	290.2 (225.3,364.5)	38.4 (7.2,75.2)
United States Virgin Islands	282 (236,334)	291.9 (248.6,343)	433 (324,583)	293.3 (215.6,400.7)	0.5 (−28.7,41.9)
**Tropical Latin America**	**128461 (122884,135259)**	**123.9 (117.9,130.8)**	**519302 (490856,546943)**	**197.1 (186.1,207.5)**	**59 (49.9,68.6)**
Brazil	126007 (120295,132775)	124.5 (118.4,131.4)	507741 (480197,534475)	197.4 (186.5,207.9)	58.6 (49.3,68.4)
Paraguay	2454 (2048,2902)	100.3 (84.6,118)	11561 (8865,14888)	184.7 (142.5,236.7)	84.2 (35.3,154.8)
**East Asia**	**870015 (734410,1023589)**	**89.9 (76.9,105)**	**4046069 (3325047,4888813)**	**186.5 (153.1,226.5)**	**107.5 (61.9,170.5)**
China	833018 (699299,986165)	89.5 (76.1,105)	3891707 (3166654,4734814)	185.7 (150.8,227)	107.6 (60.3,172.7)
Democratic People's Republic of Korea	15767 (11690,21370)	87 (65.6,115.1)	40691 (29983,52920)	119.6 (88.8,154.6)	37.4 (−0.4,84.5)
Taiwan (Province of China)	21230 (19543,23186)	119 (109.3,130.5)	113671 (104919,122316)	292.7 (269.2,314.8)	145.9 (119.8,175.1)
**Southeast Asia**	**269389 (233405,316767)**	**90.2 (78.7,105.6)**	**1189332 (1031355,1388436)**	**162.3 (141.4,188.6)**	**80 (56,106.5)**
Cambodia	4059 (2845,5742)	77.3 (56,106.9)	21181 (15994,27735)	148.5 (113.5,191.5)	92.1 (29.4,194.9)
Indonesia	99680 (75751,133267)	84.8 (65.3,111.8)	406121 (293737,552393)	141.8 (104.3,191.3)	67.2 (30.8,110.3)
Lao People's Democratic Republic	1582 (1044,2368)	67.9 (46.4,99.2)	6325 (4717,8259)	111.3 (84.4,144.5)	64 (5.3,146)
Malaysia	15073 (12924,17618)	134.9 (116.4,157.2)	81941 (70186,94992)	261.9 (225.3,302.4)	94.2 (61.5,135)
Maldives	57 (34,89)	51.5 (32.5,78.2)	363 (287,452)	83.1 (68,100.5)	61.3 (−4.7,175.4)
Mauritius	807 (733,884)	99.9 (90.8,109.8)	4390 (4019,4685)	244.8 (223.1,261.5)	144.9 (115.4,174.9)
Myanmar	25651 (18508,34862)	94.1 (70.3,125.4)	79162 (62723,99864)	145.1 (116.4,181.7)	54.2 (6.3,124.5)
Philippines	41556 (37177,45763)	115.1 (102.3,127)	169085 (139010,205206)	176.7 (146.6,211.9)	53.5 (23.9,90)
Seychelles	68 (59,78)	121.7 (105.2,139.7)	264 (231,301)	209.8 (183.8,237.5)	72.4 (42.8,106.4)
Sri Lanka	9746 (8431,11368)	77.9 (67.5,89.7)	41312 (29302,53597)	152.5 (108.2,197.6)	95.9 (32.7,171)
Thailand	40929 (34233,48352)	96.2 (81.1,112.8)	233947 (187675,290696)	229.4 (183.1,285.3)	138.5 (81.8,218.3)
Timor−Leste	180 (127,254)	49.4 (36.8,67.6)	756 (555,980)	81.8 (60.4,105.9)	65.7 (18.6,136.1)
Viet Nam	29612 (24163,36464)	70.8 (58.4,86.6)	142827 (113728,183162)	130 (105,164.8)	83.7 (38.6,144.7)
**Oceania**	**4319 (3572,5247)**	**116.5 (98.5,139.6)**	**12331 (10355,15006)**	**127.9 (109.7,152.2)**	**9.8 (−7.9,32.9)**
American Samoa	49 (41,58)	178.5 (149.7,208.2)	142 (118,171)	278.2 (230.9,333.4)	55.8 (21.5,103.1)
Cook Islands	37 (29,47)	267.3 (212.2,333.8)	96 (76,119)	406.9 (320.9,514.3)	52.2 (8.9,111.3)
Micronesia (Federated States of)	74 (56,98)	137.2 (105.1,179.9)	165 (125,212)	193.7 (149.1,245.4)	41.2 (3.7,100)
Fiji	930 (749,1150)	197.2 (163,239)	1962 (1520,2472)	231.7 (181.9,287.7)	17.5 (−11.7,57.2)
Guam	130 (113,150)	141.6 (123.8,161.7)	315 (277,362)	160.1 (140.4,182.7)	13 (−5.9,33.1)

Kiribati	52 (43,63)	121.9 (101,144.6)	138 (109,180)	160.7 (128.9,204.9)	31.8 (1.5,77.8)
Marshall Islands	23 (18,31)	120.6 (94,157.1)	74 (48,110)	164.7 (112.8,237.8)	36.5 (−1.7,81.6)
Nauru	10 (6,14)	156.3 (106,225)	18 (11,26)	242.8 (165.2,347.6)	55.3 (21.8,100.9)
Niue	4 (3,5)	189.6 (152.6,235.4)	5 (4,7)	257.2 (204.9,323.5)	35.6 (−2,79)
Northern Mariana Islands	53 (39,71)	183.8 (145,232.6)	141 (119,160)	244.5 (209.7,275)	33 (2.1,71.7)
Palau	29 (23,37)	262.3 (213.6,328.4)	64 (51,79)	276.9 (225.1,340.1)	5.6 (−21.4,39.8)
Papua New Guinea	2218 (1646,2985)	94.4 (72.7,124.4)	7411 (5627,9821)	103.4 (81.1,133.6)	9.5 (−22.4,57.7)
Samoa	105 (86,130)	115.7 (95.2,141.2)	231 (179,297)	147.3 (115.1,187.3)	27.3 (−5.1,74)
Solomon Islands	119 (82,160)	72.9 (52.5,95)	527 (390,712)	115 (87.3,151.1)	57.7 (10.5,135.1)
Tokelau	2 (2,3)	166.9 (123,220.4)	3 (3,4)	234.1 (184.8,300.3)	40.2 (6.9,92.3)
Tonga	136 (111,167)	224.9 (185.5,272.9)	233 (180,300)	279.2 (217.5,357.5)	24.2 (−7.7,69.7)
Tuvalu	10 (7,13)	138.2 (102.9,184.6)	19 (15,25)	174.6 (135.4,228.9)	26.3 (−7.8,81.6)
Vanuatu	60 (46,79)	79.1 (61.5,101.7)	233 (178,297)	109 (85.5,136.1)	37.8 (3.4,84.3)
North Africa and Middle East	174924 (155525,198773)	90.8 (80.7,103.2)	1182963 (1073961,1304132)	221.4 (201.5,242.4)	143.7 (111.5,178.7)
Afghanistan	4854 (3156,7279)	67.8 (44.6,100.3)	16271 (8742,28426)	116.3 (70.9,188)	71.4 (24,132)
Algeria	11762 (9554,14305)	86.7 (71,105.4)	56689 (44127,72699)	139.1 (110.2,175.6)	60.3 (22.9,104.9)
Bahrain	543 (466,631)	226.2 (197.5,259.6)	4922 (3965,6133)	416.6 (340.7,511.3)	84.2 (43,134.1)
Egypt	31982 (27828,36945)	97 (84.1,112)	179449 (146094,217184)	235.8 (197.1,281.4)	143.1 (90.3,208.5)
Iran (Islamic Republic of)	33731 (29073,39647)	110.3 (95.7,128.3)	237014 (214964,263602)	257.1 (233.3,284.3)	133.2 (97.8,177.1)
Iraq	11895 (9497,14984)	131 (105.1,165.8)	85949 (63105,113069)	284.6 (212.6,370.6)	117.2 (54.1,201)
Jordan	2796 (2214,3574)	167.3 (136.1,208.9)	27977 (20805,36427)	305.4 (231.8,394.7)	82.5 (22.1,160.9)
Kuwait	1377 (1230,1541)	160.7 (142,179.9)	11715 (10210,13528)	272.5 (238.3,310.2)	69.6 (43.8,97.2)
Lebanon	5181 (3978,6724)	224 (174.7,288.9)	29617 (24582,35324)	501.7 (416,600.6)	123.9 (58,213.7)
Libya	2215 (1828,2706)	105.1 (88,125.9)	14671 (11155,19851)	225.5 (177,292.9)	114.5 (56.5,192.3)
Morocco	9886 (8126,12102)	63.5 (52.7,76.5)	50152 (36003,70500)	132.5 (96.5,183.9)	108.6 (48.6,185.8)
Oman	411 (332,519)	53.6 (43.8,65.5)	2280 (1767,2882)	88 (71.8,107.5)	64.2 (22.4,116.1)
Palestine	1927 (1472,2512)	201.9 (156.1,259.7)	10851 (9055,12982)	356.4 (298.9,421.2)	76.5 (30.2,137.5)
Qatar	370 (296,449)	181.8 (153.2,217.1)	5478 (4021,7333)	387.5 (306.7,488.1)	113.1 (53.7,186.3)
Saudi Arabia	5336 (4188,6807)	75 (61.4,93.3)	55860 (41114,76362)	177.5 (142.1,224.6)	136.7 (77,220.9)
Sudan	5433 (3898,7426)	52.3 (38.9,69.5)	24488 (15462,37576)	93.2 (62.4,138.4)	78.2 (21.9,163.2)
Syrian Arab Republic	6755 (5357,8309)	111.3 (90.4,135.2)	32536 (25252,41939)	221.1 (173.6,282.2)	98.7 (40.1,183.1)
Tunisia	6611 (5585,7906)	122.3 (104,145.1)	33561 (25349,44404)	241.7 (183.3,319.5)	97.7 (42.6,174.6)
Turkey	28107 (23309,32780)	74.2 (61.9,86.5)	274666 (223638,331439)	284.2 (231.9,341.7)	282.9 (199.3,383.2)
United Arab Emirates	1032 (772,1347)	166.6 (133.9,205.2)	13034 (9645,17166)	258.4 (200.4,330.3)	55.1 (15.3,109.2)
Yemen	2623 (1919,3367)	48.2 (36.7,59.9)	14682 (10598,20056)	79.9 (59.8,106.7)	65.8 (19.6,132.7)
South Asia	347822 (312744,390038)	50.3 (45.4,56.3)	1646969 (1463695,1853540)	99.1 (88.4,111.4)	96.9 (71.3,125.7)
Bangladesh	19636 (15444,25942)	33.5 (26.9,43.3)	114382 (89647,141453)	73.7 (58.3,91.1)	119.8 (60.5,209.7)
Bhutan	116 (90,147)	39.1 (30.7,48.6)	424 (311,570)	62.7 (46.9,83.1)	60.5 (12.7,126.5)
India	267001 (234319,302249)	47.7 (42,54.1)	1264158 (1098827,1461187)	95.6 (83.3,110.7)	100.7 (71.9,136)
Nepal	4279 (3363,5304)	38.1 (30.5,46.5)	17070 (12962,22473)	65.9 (50.5,86.2)	72.9 (27.2,142.3)
Pakistan	56790 (47151,67519)	89.4 (74.5,106)	250935 (186627,326034)	160.8 (121.6,204.4)	79.8 (33.6,135)
Southern Sub−Saharan Africa	34008 (29227,38271)	111.8 (95.1,126.3)	115065 (104803,126380)	177.5 (162.9,193.2)	58.8 (38.3,85.6)
Botswana	576 (419,777)	91.2 (68.6,119.1)	2306 (1635,3254)	130.4 (98,174.7)	43 (3.8,112)
Lesotho	707 (540,937)	80.2 (62.3,103.7)	1624 (1101,2264)	137 (96.5,187.9)	70.8 (10.1,171.5)
Namibia	725 (615,853)	101.3 (86.9,117.8)	3627 (2482,5081)	223.8 (158,305)	121 (53.9,201)
South Africa	27631 (23294,31511)	118.5 (98.8,135.5)	92037 (83129,101731)	180.1 (164,198.2)	52 (30.7,80.6)
Eswatini	311 (247,382)	94.3 (76,113.4)	1054 (655,1577)	158.2 (104,231.4)	67.7 (8,161.7)
Zimbabwe	4058 (3303,4989)	90.4 (74.2,110.3)	14417 (10761,19352)	168.4 (130.2,218.3)	86.1 (37,159.2)
Western Sub−Saharan Africa	71334 (60362,82451)	72.9 (62.4,83.8)	312088 (240861,401690)	128.5 (102.9,161.1)	76.3 (42,125)
Benin	1212 (1012,1423)	55.6 (46.4,64.9)	4763 (3515,6215)	75.2 (57.6,96.1)	35.2 (3.7,77.9)
Burkina Faso	4384 (3487,5413)	90.6 (73.3,109.9)	13274 (9706,17334)	116.9 (89.6,148)	29.1 (−2.3,70.9)
Cameroon	3719 (3048,4463)	72.1 (59.9,85.9)	16440 (12124,22458)	102 (77.7,135.5)	41.5 (3.2,94.4)
Cabo Verde	209 (174,249)	99.8 (82.2,118.9)	606 (481,762)	124.3 (100.1,155.4)	24.5 (−7,66.1)
Chad	1379 (1059,1706)	46.1 (36.2,57.1)	4160 (3074,5417)	57.2 (44,73)	23.9 (−9.1,68.4)
CÃ´te d'Ivoire	3674 (2936,4699)	74.1 (60.7,91.3)	17237 (12655,22880)	120.5 (92.9,155.7)	62.7 (20.1,126.5)
Gambia	134 (105,166)	34 (27.2,41.5)	682 (519,888)	57.7 (44.8,73.9)	69.9 (26.8,128.8)
Ghana	6770 (5358,8428)	88.6 (71.6,109)	27805 (21451,36258)	133.1 (105.2,169.3)	50.2 (11.7,103.2)
Guinea	2017 (1583,2466)	57.1 (45.4,69.2)	5720 (4184,7798)	85.3 (64.4,113.5)	49.6 (5.7,110.1)
Guinea−Bissau	319 (235,441)	68.1 (51.9,90.6)	1032 (758,1366)	104.2 (78.5,134.8)	53 (12.1,109)
Liberia	619 (497,753)	49.9 (40.5,60.3)	2555 (1813,3625)	88.1 (65.1,117.8)	76.6 (22,149.8)
Mali	3002 (2515,3587)	65.9 (55.6,77.5)	9579 (7163,12770)	89 (68,116.1)	35.1 (3.5,77.8)
Mauritania	734 (558,949)	69.1 (53.6,88.4)	2923 (2266,3779)	117.5 (92.3,150.2)	70.2 (19.7,146.1)
Niger	1287 (1022,1618)	38.9 (31.7,48)	4767 (3463,6385)	48.1 (35.7,63.2)	23.5 (−7.4,65)
Nigeria	37612 (29434,47627)	78.3 (61.9,97.4)	182511 (125675,259461)	161.9 (117.9,223.3)	106.7 (44.9,193.3)
Sao Tome and Principe	41 (35,49)	63.1 (53.7,74.5)	165 (126,214)	118.7 (92.6,149.2)	88.1 (43.6,149.1)
Senegal	2105 (1703,2557)	58.1 (47.5,70.3)	8997 (6985,11859)	99 (78.9,127.8)	70.3 (28.1,125.1)
Sierra Leone	1072 (825,1360)	48.4 (37.9,60.2)	3682 (2687,4773)	78 (59.2,98.8)	61.3 (23,114)
Togo	1041 (844,1280)	68 (56.2,82.6)	5186 (3758,6905)	106.1 (79.8,138.5)	56.1 (15.6,110.6)
Eastern Sub−Saharan Africa	65603 (55706,77127)	75.2 (64.4,88.2)	250404 (213576,293278)	116.3 (101.5,132.6)	54.6 (26.1,92.1)
Burundi	2028 (1541,2764)	77.6 (59.7,105)	4663 (3527,6150)	74.4 (58.3,96.2)	−4.2 (−35,40.1)
Comoros	194 (146,255)	84.9 (66,108.9)	760 (595,971)	134.3 (106.9,170.4)	58.2 (16.9,118.5)
Djibouti	155 (116,201)	86.8 (68.1,109.4)	1029 (715,1490)	121.2 (88.3,168.4)	39.7 (0.4,106.4)
Eritrea	1250 (959,1658)	86.2 (68.7,111.2)	4690 (3401,6268)	131 (99.6,168.6)	51.9 (15.7,103.7)
Ethiopia	17340 (12048,24639)	74.6 (54.4,102.1)	57363 (47218,69740)	104.1 (88,125)	39.5 (−2.8,105.5)
Kenya	6453 (4943,8385)	67.8 (52.5,86.1)	36236 (26485,48713)	125.2 (93.7,165.7)	84.8 (41.9,143.8)

**Madagascar**	**4572 (3764,5623)**	**77.8 (65.2,93.7)**	**15140 (11294,20228)**	**98.5 (75.1,126.8)**	**26.6 (−4.6,72.1)**
**Malawi**	**2831 (2306,3431)**	**63.9 (52.8,76.4)**	**9989 (7380,12940)**	**107.2 (81.9,137.1)**	**67.7 (24,120.8)**
**Mozambique**	**4559 (3839,5477)**	**66.6 (56.9,78.8)**	**14621 (10600,18998)**	**104.4 (78.5,132.3)**	**56.9 (15.1,107.7)**
**Rwanda**	**3359 (2457,4586)**	**100.4 (75.5,135)**	**10334 (7557,13781)**	**133.6 (100,175.4)**	**33 (−16.7,107.1)**
**Somalia**	**1972 (1436,2613)**	**62.6 (47.6,79.4)**	**5544 (3851,7498)**	**69.7 (51.6,90.3)**	**11.4 (−16.4,46)**
**South Sudan**	**1668 (1272,2227)**	**59.4 (46.3,78)**	**4136 (3055,5752)**	**82.8 (62.5,111.7)**	**39.3 (−1.1,93.5)**
**United Republic of Tanzania**	**10345 (8592,12333)**	**83.7 (70.4,99.2)**	**39225 (29842,50471)**	**123.2 (95.4,155.6)**	**47.3 (10.6,96.8)**
**Uganda**	**6110 (4692,7958)**	**85 (67.2,108.4)**	**29456 (22127,38421)**	**159.2 (123.3,203.2)**	**87.4 (36.4,156.3)**
**Zambia**	**2719 (2043,3613)**	**77.5 (59.8,100.7)**	**17000 (9776,26738)**	**169.4 (106,251.3)**	**118.6 (21.3,277.8)**
**Central Sub−Saharan Africa**	**17996 (13624,23444)**	**68.5 (53.1,86.8)**	**73445 (57161,93143)**	**105.2 (83.2,131.8)**	**53.5 (16.5,98.3)**
**Angola**	**2851 (2134,3822)**	**60 (46.5,77.7)**	**16972 (12127,22329)**	**109.1 (80.4,141.2)**	**81.8 (28.6,148.7)**
**Central African Republic**	**944 (708,1259)**	**71 (54.3,91.4)**	**2350 (1661,3216)**	**83 (62.3,107.1)**	**16.9 (−14.7,62.3)**
**Congo**	**1274 (847,1932)**	**106.2 (72.5,156)**	**5984 (3761,9302)**	**166 (110.8,249.1)**	**56.3 (12.8,123.5)**
**Democratic Republic of the Congo**	**12122 (8988,15856)**	**66.3 (50.9,85.2)**	**44697 (34166,59010)**	**97.4 (75.7,126)**	**47.1 (9,102.2)**
**Equatorial Guinea**	**157 (114,214)**	**72.4 (54.2,95.4)**	**1266 (776,1986)**	**184.1 (119.6,275.2)**	**154.4 (65.7,291.2)**
**Gabon**	**647 (487,842)**	**109.1 (83.2,140.8)**	**2175 (1552,2944)**	**176 (128.9,233.7)**	**61.2 (12,127.6)**

Table S3: Number of Deaths from Breast Cancer and Percentage Change in Age−Standardized Rates (ASRs) per 100,000 Population, 1990 and 2021<br>Percentage change in age−standardized rates (ASRs) per 100,000 population, by location<br>(Generated from data provided by http://ghdx.healthdata.org/gbd−results−tool)					
	1990		2021		Percentage change in the ASRs per 100,000
	No (95% UI)	ASRs per 100,000 (95% UI)	No (95% UI)	ASRs per 100,000 (95% UI)	
Global	355132 (335138,373138)	9.2 (8.6,9.6)	673560 (622742,720164)	7.9 (7.3,8.4)	−13.7 (−18.1,−8.9)
High−income North America	54009 (50555,56022)	15.8 (14.9,16.4)	59585 (53301,63188)	9.3 (8.5,9.8)	−41.2 (−43.8,−39)
Canada	4822 (4433,5138)	15.1 (13.9,16.1)	6110 (5298,6771)	8.6 (7.5,9.4)	−43.2 (−48.6,−37.3)
Greenland	6 (5,7)	16.6 (13.6,20.4)	6 (4,8)	8.4 (6.3,10.8)	−49.4 (−63.5,−29.5)
United States of America	49180 (45887,50991)	15.9 (14.9,16.5)	53469 (47929,56786)	9.4 (8.5,9.9)	−40.9 (−43.7,−38.7)
Australasia	3286 (3079,3478)	14.5 (13.6,15.3)	4368 (3749,4929)	8.3 (7.3,9.3)	−42.5 (−48.2,−36)
Australia	2613 (2436,2779)	13.8 (12.9,14.7)	3606 (3091,4099)	8.1 (7.1,9.1)	−41.3 (−47.3,−33.9)
New Zealand	673 (625,719)	17.8 (16.6,19)	762 (658,841)	9.5 (8.3,10.4)	−46.9 (−51.8,−41.2)
High−income Asia Pacific	7830 (7471,8075)	3.8 (3.7,4)	20225 (16844,22164)	4.9 (4.3,5.2)	27.3 (16.6,34.4)
Brunei Darussalam	11 (8,14)	8.2 (6.2,10.7)	38 (30,47)	9.3 (7.3,11.4)	14.1 (−18.6,58.6)
Japan	6603 (6279,6811)	4 (3.8,4.1)	16824 (13897,18482)	5.4 (4.8,5.8)	37 (27.1,43.2)
Singapore	196 (185,209)	8 (7.5,8.4)	483 (436,523)	5.6 (5,6.1)	−29.6 (−35.7,−22.8)
Republic of Korea	1019 (895,1211)	3 (2.6,3.5)	2879 (2297,3467)	3.3 (2.6,4)	11.3 (−16.7,36.1)
Western Europe	91960 (86182,95294)	16.6 (15.7,17.2)	93872 (80678,101168)	9.9 (8.8,10.6)	−40.4 (−44,−37.6)
Andorra	7 (5,10)	12 (8.4,17.3)	14 (10,19)	8.9 (6.2,12.1)	−26.1 (−53,15.4)
Austria	1871 (1727,2002)	16.4 (15.2,17.5)	1730 (1483,1925)	9.2 (8,10.2)	−43.9 (−50.1,−38.1)
Belgium	2936 (2667,3125)	20 (18.3,21.2)	2586 (2160,2910)	10.8 (9.4,12)	−45.8 (−50.5,−40.4)
Cyprus	101 (82,122)	13.8 (11.1,16.9)	212 (175,256)	11.4 (9.4,13.6)	−17.6 (−37.1,7.4)
Denmark	1735 (1617,1834)	22.4 (21,23.7)	1325 (1151,1461)	10.9 (9.5,12)	−51.5 (−56.2,−46.8)
Finland	908 (853,965)	13.2 (12.4,14)	1037 (889,1149)	8.3 (7.3,9.1)	−37.2 (−43.7,−30.7)
France	12969 (12005,13778)	16.2 (15.1,17.1)	15357 (12964,17077)	10.6 (9.3,11.7)	−34.5 (−40.2,−28.3)
Germany	20717 (19234,22033)	16.8 (15.7,17.9)	20860 (17921,23045)	10.8 (9.5,11.7)	−36.1 (−41.6,−30.4)
Greece	1998 (1863,2112)	13.9 (12.9,14.6)	2907 (2488,3188)	11.8 (10.5,12.7)	−15.2 (−21.5,−7.8)
Iceland	40 (37,44)	14.4 (13.2,15.6)	52 (44,58)	9 (7.8,10)	−37.8 (−45.5,−30.1)
Ireland	704 (661,750)	18.3 (17.2,19.4)	722 (628,806)	9.3 (8.1,10.3)	−49.4 (−55.2,−43.3)
Israel	768 (713,822)	16.7 (15.5,17.8)	1270 (1073,1412)	10.2 (8.7,11.2)	−39.2 (−46.3,−32)
Italy	13038 (12145,13599)	15.6 (14.5,16.2)	14640 (12141,16198)	9.9 (8.6,10.8)	−36.4 (−41,−32.7)
Luxembourg	100 (93,106)	18.9 (17.8,20.2)	104 (91,116)	9.5 (8.4,10.6)	−49.8 (−55.6,−43.5)
Malta	81 (75,88)	19.3 (17.7,21)	99 (84,113)	10.7 (9.3,12.1)	−44.5 (−50.9,−36.5)
Monaco	12 (9,16)	18.7 (13.6,24.6)	17 (13,23)	18.9 (14,25)	1.2 (−28.1,58.3)
Netherlands	3728 (3360,3990)	19.1 (17.4,20.4)	3777 (3305,4197)	11 (9.8,12)	−42.7 (−48.3,−37.5)
Norway	855 (789,898)	13.2 (12.3,13.8)	736 (643,806)	7.2 (6.4,7.9)	−45.5 (−49.7,−41.5)
Portugal	1827 (1706,1947)	14.4 (13.5,15.3)	2103 (1819,2317)	8.8 (7.8,9.5)	−39.2 (−44.8,−33.9)
San Marino	4 (3,5)	11.1 (8.6,13.9)	4 (3,6)	5.9 (3.5,8.5)	−47.2 (−69,−19.2)
Spain	6747 (6182,7232)	13.2 (12.2,14.1)	7213 (6084,8149)	7.3 (6.4,8.2)	−44.9 (−50.2,−39.2)
Sweden	1669 (1535,1789)	12 (11.1,12.8)	1614 (1336,1854)	7.3 (6.1,8.3)	−39 (−48.5,−30.1)
Switzerland	1427 (1313,1522)	14.1 (13.1,15)	1495 (1250,1685)	7.9 (6.8,8.8)	−43.9 (−49.8,−38.4)
United Kingdom	17642 (16627,18137)	20.7 (19.6,21.3)	13914 (12357,14738)	10.6 (9.6,11.2)	−48.7 (−51.1,−47)
Southern Latin America	7163 (6750,7503)	15.8 (14.8,16.6)	10081 (9077,10941)	11.6 (10.5,12.6)	−26.4 (−32.4,−19.6)
Uruguay	755 (708,799)	20.1 (18.9,21.3)	933 (824,1032)	16.9 (15.1,18.4)	−16.3 (−24.4,−7.5)
Argentina	5434 (5083,5717)	17.2 (16.1,18.2)	7371 (6596,8040)	13.3 (11.9,14.4)	−23 (−30.4,−15.6)
Chile	974 (908,1043)	9.8 (9,10.5)	1776 (1581,1954)	7 (6.2,7.7)	−28.3 (−35.6,−20.4)
Eastern Europe	30019 (29053,30864)	11 (10.6,11.3)	36121 (32261,40811)	10.5 (9.4,11.9)	−4.2 (−14.3,7.6)
Belarus	1248 (1136,1361)	9.9 (9,10.8)	1363 (1085,1697)	8.7 (6.9,10.9)	−11.6 (−30.6,12.6)
Estonia	253 (233,273)	12.6 (11.6,13.7)	248 (201,296)	9.1 (7.4,10.8)	−28.1 (−41.1,−13.9)
Latvia	418 (374,469)	12 (10.7,13.4)	422 (350,494)	10.8 (8.9,12.8)	−9.3 (−25.4,9.4)
Lithuania	471 (432,510)	10.7 (9.8,11.5)	567 (470,670)	10.2 (8.5,11.9)	−4.5 (−21.2,13.1)
Republic of Moldova	544 (486,599)	12.3 (11,13.5)	591 (501,693)	10.1 (8.5,11.8)	−18.3 (−32.6,−0.6)
Russian Federation	17350 (16827,17793)	9.8 (9.5,10.1)	25302 (22721,27818)	10.8 (9.7,11.9)	10.1 (−1.8,20.5)
Ukraine	9735 (9062,10409)	14.1 (13.1,15)	7628 (5022,10942)	10.2 (6.6,14.7)	−27.7 (−52.5,5.1)
Central Europe	17645 (16919,18406)	12.2 (11.7,12.8)	25214 (22971,27334)	11.6 (10.6,12.6)	−5.2 (−12.7,3)
Albania	105 (83,132)	5 (3.9,6.4)	229 (161,308)	5.7 (4,7.6)	12.9 (−26.9,70.5)
Bosnia and Herzegovina	319 (273,360)	7.6 (6.6,8.5)	577 (458,710)	9.5 (7.5,11.7)	25.1 (−4,64.1)
Bulgaria	1312 (1152,1496)	11.4 (10,13)	1758 (1440,2080)	13.2 (10.8,15.6)	15.5 (−9.1,44.9)
Croatia	831 (735,938)	14.4 (12.7,16.2)	957 (819,1106)	10.8 (9.2,12.4)	−24.9 (−38.5,−10.6)
Czechia	1948 (1757,2175)	14.5 (13,16.1)	1983 (1647,2330)	9.3 (7.8,10.9)	−35.9 (−47.4,−21)
Hungary	2152 (1916,2415)	15.4 (13.7,17.4)	2223 (1888,2576)	11.6 (9.9,13.5)	−24.6 (−37.4,−10.4)
Montenegro	79 (61,103)	12.6 (9.7,16.4)	136 (106,173)	14.8 (11.5,18.7)	17.3 (−17.8,69)
North Macedonia	235 (193,282)	12.2 (9.9,14.7)	418 (325,524)	13.9 (10.9,17.5)	14.6 (−13.9,52.7)
Poland	5059 (4855,5227)	12 (11.5,12.4)	8615 (7566,9569)	12.1 (10.7,13.4)	1 (−10.1,10.8)
Romania	2656 (2489,2861)	9.8 (9.2,10.5)	4135 (3604,4719)	11.5 (10,13)	17.6 (0.5,35.1)
Serbia	1644 (1244,2105)	15.7 (11.9,20.3)	2342 (1783,3005)	14.6 (11.2,18.8)	−7.1 (−35,33.6)
Slovakia	702 (601,827)	12 (10.3,14.1)	1043 (781,1325)	11.2 (8.5,14.2)	−6.5 (−33.5,26.1)
Slovenia	320 (294,348)	13.1 (12,14.2)	431 (356,514)	9.1 (7.5,10.9)	−30.2 (−42.4,−17.3)
Central Asia	5165 (4865,5435)	10.7 (10.1,11.3)	6643 (5944,7414)	7.8 (7,8.6)	−27.6 (−35.5,−19.1)

Armenia	454 (421,486)	16 (14.8,17.2)	503 (440,583)	11.6 (10.2,13.5)	−27.2 (−37.7,−14.3)
Azerbaijan	507 (424,577)	9.8 (8.3,11.1)	868 (651,1117)	7.8 (6.9,8)	−21.1 (−40.8,1.1)
Georgia	959 (867,1058)	15.6 (14.1,17.2)	948 (818,1088)	16.4 (14.1,18.9)	5 (−12.9,22.4)
Kazakhstan	1637 (1444,1827)	12.6 (11.1,14.1)	1380 (1145,1607)	7.6 (6.3,8.8)	−40 (−50.8,−26.5)
Kyrgyzstan	306 (276,339)	10.2 (9.2,11.3)	335 (274,399)	6.4 (5.3,7.7)	−37 (−47.8,−22.1)
Mongolia	33 (25,41)	2.9 (2.2,3.7)	85 (66,104)	3.3 (2.5,4.1)	12.7 (−20.4,56.6)
Tajikistan	218 (175,265)	7.6 (6.1,9.3)	376 (236,553)	5.4 (3.6,7.7)	−28.6 (−53.7,3.9)
Turkmenistan	157 (137,178)	7.7 (6.7,8.7)	304 (226,406)	6.8 (5.1,8.9)	−12.3 (−34.2,17.4)
Uzbekistan	895 (796,992)	7.5 (6.7,8.4)	1846 (1536,2213)	6.2 (5.1,7.4)	−18.2 (−32.9,0.6)
Central Latin America	5637 (5467,5779)	6.3 (6.1,6.5)	19479 (17023,21928)	7.6 (6.7,8.6)	20.2 (5.6,35.5)
Colombia	1422 (1331,1512)	7.6 (7.1,8.1)	4232 (3539,5038)	7.7 (6.4,9.1)	0.7 (−16.7,22.1)
Costa Rica	118 (109,127)	6.5 (6,7)	493 (428,559)	8.9 (7.8,10.2)	37.5 (18.5,59.6)
El Salvador	133 (116,151)	4.3 (3.7,4.8)	422 (335,522)	6.8 (5.4,8.5)	60.6 (22.8,107.2)
Guatemala	114 (107,121)	3.2 (3,3.4)	548 (460,639)	4.8 (4,5.6)	50.6 (28.1,75)
Honduras	91 (65,119)	4.1 (2.8,5.6)	473 (341,651)	7.1 (5.2,9.7)	73.2 (22.7,158.2)
Mexico	2825 (2741,2903)	6.2 (6,6.4)	9492 (7987,11086)	7.3 (6.2,8.5)	17.5 (−1.1,36.6)
Nicaragua	57 (48,66)	3.3 (2.8,3.9)	249 (194,312)	4.8 (3.8,6)	45.7 (12.7,89.9)
Panama	91 (84,97)	5.9 (5.5,6.3)	332 (264,398)	7.5 (5.9,9)	26.4 (0.5,52.8)
Venezuela (Bolivarian Republic of)	787 (744,834)	7.5 (7,7.9)	3239 (2477,4091)	10.7 (8.2,13.5)	43.1 (9.7,85)
Andean Latin America	1379 (1177,1600)	6.3 (5.4,7.3)	4114 (3253,5191)	6.8 (5.4,8.6)	7.8 (−13.7,33.4)
Bolivia (Plurinational State of)	283 (186,409)	8.4 (5.6,12)	890 (588,1290)	9.5 (6.4,13.7)	13.9 (−19.4,63.1)
Ecuador	267 (251,283)	4.8 (4.5,5)	1107 (863,1397)	6.8 (5.3,8.5)	42.2 (10.6,79.3)
Peru	829 (685,984)	6.5 (5.3,7.7)	2118 (1536,2832)	6.1 (4.4,8.3)	−5.2 (−33.3,33.1)
Caribbean	2635 (2428,2877)	10.2 (9.4,11.1)	5684 (4806,6679)	10.6 (8.9,12.4)	3.8 (−10.1,18.4)
Antigua and Barbuda	8 (7,9)	14.7 (13.2,16.3)	18 (17,19)	16.8 (15.7,18.2)	14.8 (0.9,29.7)
Barbados	49 (45,53)	18.1 (16.6,19.5)	96 (78,119)	19.4 (15.8,23.9)	7.5 (−13.7,32.9)
Belize	5 (4,5)	5 (4.5,5.4)	22 (19,25)	6.9 (6.1,7.7)	37.9 (17.9,59.4)
Bermuda	13 (11,14)	20.9 (18.8,22.8)	15 (12,19)	11.2 (9.2,14)	−46.5 (−56.2,−32.6)
Bahamas	32 (30,35)	19.5 (17.9,21.2)	85 (69,105)	20.7 (16.9,25.2)	6.1 (−15.5,30.6)
Cuba	997 (938,1056)	9.9 (9.3,10.4)	1845 (1584,2152)	9.5 (8.1,11.1)	−4 (−18.1,11)
Dominica	10 (8,11)	16.6 (14.1,19.2)	14 (10,17)	17 (13.1,21.2)	2.5 (−24,39)
Dominican Republic	239 (201,281)	6.3 (5.4,7.4)	755 (579,980)	7.4 (5.7,9.6)	17.3 (−14.9,63.6)
Grenada	10 (9,11)	15 (13.6,16.6)	19 (16,21)	17.3 (15.1,19.4)	15.7 (−2,33.7)
Guyana	44 (39,50)	10.9 (9.6,12.4)	91 (69,118)	13.6 (10.4,17.4)	24.4 (−10.4,62.4)
Haiti	394 (239,610)	11.6 (7.3,17.4)	1078 (666,1672)	13.4 (8.5,20.2)	16 (−21.9,75.5)
Jamaica	190 (175,205)	10.9 (10.1,11.8)	463 (352,585)	14.7 (11.2,18.6)	35.1 (3,73.2)
Puerto Rico	365 (339,389)	10.4 (9.7,11.1)	592 (486,694)	8.8 (7.3,10.4)	−15.5 (−29.5,0.8)
Saint Kitts and Nevis	8 (7,9)	23.5 (21.7,25.6)	11 (9,13)	16.4 (13.6,19.2)	−30.2 (−42.2,−16.9)
Saint Lucia	14 (13,15)	17.1 (15.9,18.4)	30 (25,36)	12.6 (10.5,15)	−26.6 (−39.7,−11.2)
Saint Vincent and the Grenadines	11 (10,12)	16.8 (15.1,18.2)	21 (18,24)	15.4 (13.5,17.5)	−8.6 (−20.5,6.8)
Suriname	24 (20,27)	9 (7.6,10.4)	62 (47,79)	9.7 (7.5,12.4)	8.3 (−19.4,42)
Trinidad and Tobago	119 (111,128)	14.7 (13.7,15.7)	257 (198,325)	13.7 (10.6,17.4)	−6.9 (−28.6,18.4)
United States Virgin Islands	14 (12,17)	16.9 (14.3,20.2)	18 (13,25)	11.7 (8.2,16.1)	−30.8 (−51.4,−2.3)
Tropical Latin America	8524 (8170,8837)	9 (8.5,9.4)	24406 (22588,25912)	9.4 (8.7,10)	4.9 (−0.5,10.5)
Brazil	8371 (8022,8685)	9 (8.5,9.4)	23841 (22077,25282)	9.4 (8.7,10)	4.2 (−1.1,9.8)
Paraguay	153 (124,185)	6.6 (5.3,8)	565 (419,755)	9.5 (7,12.6)	44.4 (1,107.8)
East Asia	42915 (35281,51921)	4.7 (3.9,5.6)	96374 (76528,118967)	4.5 (3.6,5.5)	−5 (−28.9,28.8)
China	41143 (33558,50103)	4.7 (3.9,5.7)	91458 (71716,113682)	4.4 (3.5,5.5)	−6.3 (−31.2,28.6)
Democratic People's Republic of Korea	991 (655,1436)	5.7 (3.8,8.2)	2072 (1449,2764)	6.2 (4.4,8.2)	8.1 (−28,58.4)
Taiwan (Province of China)	781 (738,822)	4.6 (4.4,4.9)	2844 (2564,3101)	7 (6.4,7.7)	52.7 (37.9,67.4)
Southeast Asia	20300 (17014,24597)	7.2 (6.1,8.6)	66219 (55015,80682)	9.6 (8,11.5)	32.7 (13.5,56.5)
Cambodia	381 (243,578)	7.7 (5.1,11.4)	1510 (1086,2005)	11.5 (8.4,15.1)	50.7 (−6.8,148.5)
Indonesia	7986 (5491,11328)	7.2 (4.9,10.1)	25977 (17191,37629)	9.9 (6.5,14.2)	38.8 (2.1,87.8)
Lao People's Democratic Republic	164 (97,264)	7.4 (4.6,11.5)	488 (345,675)	9.4 (6.8,12.9)	27.6 (−23.6,116.5)
Malaysia	1074 (909,1253)	10.3 (8.8,11.9)	3899 (3318,4534)	13.2 (11.3,15.4)	28.8 (4.9,60.8)
Maldives	4 (2,7)	4.1 (2.4,6.6)	14 (11,18)	3.8 (3,4.8)	−6 (−48.3,77.1)
Mauritius	49 (45,52)	6.5 (6,7)	225 (203,239)	12.5 (11.3,13.3)	92.2 (69.6,111.1)
Myanmar	2391 (1648,3392)	9.1 (6.4,12.5)	5307 (4093,7105)	10.2 (7.9,13.4)	12.5 (−26.2,71.7)
Philippines	3210 (2859,3549)	10.1 (9,11.3)	11369 (8968,14160)	12.9 (10.2,15.9)	27 (−1.9,63.5)
Seychelles	5 (4,6)	8.7 (7.5,10)	14 (12,16)	11.5 (9.7,13.4)	32.7 (7.7,61.7)
Sri Lanka	628 (524,747)	5.5 (4.5,6.6)	1778 (1165,2396)	6.7 (4.4,9)	22.1 (−23.6,77.2)
Thailand	2368 (1919,2822)	5.9 (4.7,7.1)	9090 (6810,11494)	8.6 (6.5,10.9)	46.4 (5.2,107.3)
Timor−Leste	16 (10,25)	4.8 (3.2,7.2)	60 (41,82)	6.9 (4.8,9.4)	42.9 (−4.5,123.4)
Viet Nam	1995 (1554,2582)	4.9 (3.8,6.3)	6397 (4839,8454)	6.2 (4.7,8.1)	27 (−8.8,75.6)
Oceania	321 (250,404)	9.7 (7.7,12.1)	954 (781,1177)	10.9 (9.1,13.2)	12.6 (−9.1,42.5)
American Samoa	3 (3,4)	14 (11.7,16.7)	10 (8,12)	20.8 (16.5,25.7)	48.9 (13.6,94.6)
Cook Islands	2 (2,3)	19.6 (15.4,24.3)	5 (4,6)	20.6 (15.7,26.3)	5.3 (−27.3,45.9)
Micronesia (Federated States of)	7 (5,9)	13.7 (9.5,18.7)	13 (10,18)	17.8 (13.1,23.3)	30.4 (−10.3,94)
Fiji	66 (52,84)	16.4 (13,20.7)	156 (114,205)	21.1 (15.8,26.7)	28.6 (−9.5,73.3)
Guam	7 (6,8)	9.7 (8.3,11.2)	14 (12,17)	7 (5.8,8.6)	−27.3 (−40.5,−11.8)
Kiribati	5 (4,6)	13.1 (10,16.8)	14 (10,19)	18.6 (14.1,24.7)	42.1 (5.3,108.9)

Marshall Islands	2 (1,3)	11.3 (8.3,15.4)	6 (4,9)	15.4 (9.6,23)	36.1 (−6.6,84.4)
Nauru	1 (0,1)	13.9 (8.4,21.3)	1 (1,2)	21 (12.7,32.2)	50.7 (11.3,100.9)
Niue	0 (0,0)	15.6 (11.9,20.2)	0 (0,0)	18.8 (14.3,24.2)	20.6 (−16.6,66.6)
Northern Mariana Islands	2 (2,3)	12.2 (9.3,15.8)	7 (6,8)	14.6 (12.2,16.7)	19.2 (−10.5,54.5)
Palau	2 (2,3)	22.4 (17.4,28.8)	4 (3,6)	21.9 (17.3,27.7)	−2.4 (−30.5,32.5)
Papua New Guinea	170 (117,242)	7.5 (5.3,10.6)	581 (420,798)	8.7 (6.4,11.8)	14.7 (−27.6,80.2)
Samoa	8 (6,10)	9.1 (6.9,11.5)	16 (12,21)	10.9 (8.1,14.4)	19.5 (−14.3,73.3)
Solomon Islands	10 (6,14)	6.5 (4.4,9.2)	44 (31,61)	11.1 (7.9,15.1)	69.2 (13.4,156.6)
Tokelau	0 (0,0)	14.5 (9.8,20.4)	0 (0,0)	16.6 (12.2,21.9)	14.4 (−17.7,65.5)
Tonga	10 (8,12)	17.6 (14.1,21.8)	16 (12,21)	20.1 (14.9,26.5)	14.3 (−18.6,61.3)
Tuvalu	1 (1,1)	13.2 (8.9,18.6)	2 (1,2)	15.2 (11.2,20.3)	15.2 (−20,78.5)
Vanuatu	5 (3,6)	6.9 (5.9,9.5)	20 (15,26)	10.9 (8.2,13.7)	57.8 (14.9,122.2)
North Africa and Middle East	6968 (6255,7935)	3.8 (3.4,4.2)	30106 (26510,34229)	6.1 (5.4,6.9)	62.8 (43.3,85.7)
Afghanistan	335 (177,561)	4.7 (2.6,7.7)	939 (488,1677)	7.3 (4.2,11.9)	54.4 (5.9,114.3)
Algeria	452 (346,582)	3.8 (2.9,4.8)	1541 (1173,1982)	4.2 (3.2,5.3)	10.7 (−16.9,45.2)
Bahrain	22 (19,26)	11.5 (9.6,13.7)	99 (77,128)	10.6 (8.3,13.7)	−7.3 (−32.7,26.8)
Egypt	1400 (1219,1669)	4.4 (3.9,5.1)	5989 (4755,7420)	8.8 (7.1,10.7)	101.1 (55.5,155.1)
Iran (Islamic Republic of)	924 (794,1064)	3.2 (2.8,3.7)	4062 (3665,4498)	4.7 (4.3,5.3)	47.2 (25.1,76.1)
Iraq	536 (406,704)	6.1 (4.6,8)	2308 (1629,3100)	8.4 (6,11.1)	38.7 (−7.8,101.8)
Jordan	111 (85,142)	7.1 (5.5,8.9)	622 (442,835)	7.5 (5.4,10.1)	6.2 (−33.3,61.9)
Kuwait	34 (31,37)	4.8 (4.2,5.2)	165 (140,191)	4.4 (3.7,5.1)	−8.4 (−23.5,8)
Lebanon	222 (160,296)	10.3 (7.6,13.6)	675 (541,832)	11.1 (8.9,13.8)	7.9 (−26.9,56.5)
Libya	83 (66,107)	4.1 (3.3,5.2)	397 (294,546)	6.5 (4.9,8.7)	59.2 (8.5,129.6)
Morocco	453 (353,582)	3 (2.4,3.8)	1767 (1189,2540)	4.9 (3.3,6.8)	64 (12,138.9)
Oman	14 (10,19)	1.9 (1.4,2.6)	48 (36,62)	2.2 (1.7,2.7)	11.2 (−22.1,65.2)
Palestine	88 (64,121)	9.8 (7.2,13.4)	300 (242,368)	11.2 (9,13.6)	14.2 (−19.8,64.7)
Qatar	12 (10,15)	8.8 (7.1,10.9)	82 (60,113)	8.4 (6.3,11.2)	−4.4 (−32.9,35.2)
Saudi Arabia	208 (151,282)	2.9 (2.2,3.8)	1223 (867,1703)	4.1 (3.1,5.3)	41 (−3.9,108.6)
Sudan	279 (177,423)	2.7 (1.8,4)	943 (581,1505)	3.9 (2.6,6)	42.6 (−10.6,124.6)
Syrian Arab Republic	255 (194,318)	4.3 (3.3,5.4)	771 (560,1038)	5.5 (4,7.3)	26.9 (−13.5,92.7)
Tunisia	245 (200,295)	4.9 (4,5.9)	782 (563,1075)	5.8 (4.2,8)	20.4 (−17.5,72.8)
Turkey	1116 (888,1381)	3 (2.4,3.6)	6367 (4985,7922)	6.8 (5.3,8.5)	130.1 (67.7,209.4)
United Arab Emirates	44 (32,59)	8.3 (6.4,10.6)	335 (242,449)	8.9 (6.5,11.7)	7 (−26.5,50.1)
Yemen	131 (82,184)	2.4 (1.6,3.4)	664 (460,944)	3.9 (2.8,5.5)	61.5 (9.1,146.1)
South Asia	30085 (26652,34000)	4.6 (4,5.2)	107760 (94134,123109)	6.9 (6.1,7.9)	50.5 (28.1,76.4)
Bangladesh	1618 (1215,2200)	2.8 (2.1,3.7)	5725 (4347,7362)	3.7 (2.9,4.8)	35.6 (−5.4,103.5)
Bhutan	10 (7,14)	3.7 (2.7,4.9)	27 (19,37)	4.3 (3.1,5.7)	14.6 (−22.1,69.4)
India	22457 (19183,26289)	4.2 (3.6,5)	80665 (68247,95562)	6.5 (5.5,7.7)	54 (29.9,84.1)
Nepal	398 (295,519)	3.8 (2.8,4.9)	1164 (832,1612)	4.8 (3.5,6.7)	27.8 (−10.5,88)
Pakistan	5602 (4414,6990)	9.3 (7.3,11.7)	20179 (14587,26824)	14.7 (10.7,19.1)	57.3 (9.8,115.2)
Southern Sub−Saharan Africa	2741 (2278,3216)	9.9 (8.1,11.8)	8550 (7741,9442)	14.9 (13.6,16.3)	50 (26.2,85.9)
Botswana	53 (37,74)	9.7 (6.9,13.1)	184 (132,260)	12.5 (9.5,16.8)	28.6 (−9.6,102.7)
Lesotho	67 (48,93)	8.2 (5.8,11.3)	169 (107,247)	16.1 (10.4,23)	97.7 (10.5,244.6)
Namibia	68 (56,82)	10.3 (8.5,12.5)	267 (175,370)	18.3 (12.3,24.6)	78.3 (17.4,144.6)
South Africa	2165 (1732,2608)	10.1 (8,12.3)	6554 (5931,7259)	14.4 (13,15.9)	41.8 (16,80.9)
Eswatini	29 (22,37)	10 (7.6,12.7)	90 (53,141)	16.1 (9.9,24.2)	60.7 (−3.4,161.4)
Zimbabwe	359 (277,462)	9 (7,11.5)	1285 (928,1718)	17 (12.7,22.4)	89.3 (33.3,176.1)
Western Sub−Saharan Africa	7569 (6211,8945)	8.4 (7,9.9)	26212 (20480,33422)	12.4 (10.1,15.5)	46.9 (15.4,89.6)
Benin	125 (102,150)	6.1 (5,7.2)	405 (298,537)	7.4 (5.6,9.6)	21.9 (−9.8,64.1)
Burkina Faso	458 (351,579)	10.6 (8.5,13.3)	1162 (841,1543)	12.1 (9.1,15.6)	13.4 (−15.5,52.9)
Cameroon	371 (297,461)	7.9 (6.3,9.7)	1330 (954,1821)	9.8 (7.1,13.3)	24.1 (−11.2,75.5)
Cabo Verde	18 (14,22)	8.3 (6.7,10.1)	38 (30,48)	8.4 (6.5,10.7)	2 (−27.2,45.2)
Chad	142 (103,186)	5 (3.6,6.5)	394 (282,522)	6.1 (4.5,8)	23.9 (−13.9,79.2)
CÃ´te d'Ivoire	367 (284,471)	8.5 (6.8,10.6)	1373 (1002,1846)	11.2 (8.4,14.8)	32.3 (−3.2,85.6)
Gambia	12 (9,16)	3.2 (2.5,4.1)	56 (40,74)	5.3 (3.8,6.9)	65.6 (15.9,139)
Ghana	624 (481,793)	9 (7,11.5)	2032 (1520,2705)	11.3 (8.5,14.6)	24.7 (−11.6,74.4)
Guinea	219 (164,275)	6.4 (4.8,8)	529 (377,737)	8.6 (6.2,11.6)	34.1 (−8.6,99.7)
Guinea−Bissau	35 (24,51)	8 (5.7,11.3)	94 (65,128)	11.1 (7.7,14.9)	38.5 (−3.8,101.5)
Liberia	66 (52,82)	5.7 (4.5,7)	196 (135,284)	8 (5.6,11.1)	40 (−5.7,106)
Mali	318 (259,384)	7.4 (6,8.9)	827 (593,1137)	8.4 (6.2,11.4)	13.9 (−15.7,57.5)
Mauritania	79 (57,106)	7.8 (5.7,10.5)	210 (158,278)	9.3 (7.2,12.3)	18.7 (−20.3,76)
Niger	131 (98,176)	4.4 (3.2,5.9)	442 (305,609)	5.1 (3.5,6.9)	16.8 (−16.1,64.4)
Nigeria	4194 (3174,5411)	9.5 (7.3,12.1)	15655 (10775,22235)	15.9 (11.4,22)	67.5 (15,142.2)
Sao Tome and Principe	4 (3,5)	6 (4.9,7.3)	11 (8,14)	9 (6.9,11.6)	50.2 (9.6,107.3)
Senegal	201 (159,253)	6 (4.8,7.4)	729 (551,968)	9 (6.9,11.8)	50.6 (9,108.7)
Sierra Leone	108 (78,143)	5.2 (3.8,6.7)	315 (224,422)	7.6 (5.6,10.1)	47.4 (6.2,106.8)
Togo	98 (79,123)	7 (5.8,8.7)	413 (296,568)	10 (7.3,13.5)	42.6 (3.2,98.9)
Eastern Sub−Saharan Africa	7100 (5970,8590)	9.1 (7.8,11)	21242 (18196,24900)	11.9 (10.3,13.8)	29.9 (5.6,62.9)
Burundi	237 (169,333)	9.9 (7.2,13.7)	437 (319,598)	8.4 (6.3,11.5)	−14.8 (−45.5,33.8)
Comoros	20 (14,26)	9.5 (7,12.6)	67 (50,89)	13.2 (9.9,17.7)	39.3 (−2.3,105.1)
Djibouti	15 (11,20)	9.9 (7.5,12.9)	82 (55,120)	12.2 (8.7,16.8)	23.1 (−15.3,81.4)
Eritrea	138 (100,188)	10.9 (8.2,14.2)	436 (306,599)	15 (10.7,20)	37.8 (2.5,89.2)
Ethiopia	2065 (1376,3036)	10.3 (7.2,14.5)	4846 (3967,5940)	10.5 (8.7,12.8)	2.8 (−29.4,52.6)
Kenya	575 (416,780)	6.6 (4.8,8.9)	2784 (1966,3827)	11.5 (8.3,15.6)	74.7 (28.8,140.5)
Madagascar	472 (380,584)	8.7 (7.2,10.6)	1260 (889,1689)	9.9 (7.2,12.8)	13 (−20.3,60.7)

**Malawi**	**299 (237,368)**	**7.3 (5.8,9)**	**898 (649,1185)**	**11.5 (8.6,14.9)**	**57.1 (14.8,110.2)**
**Mozambique**	**489 (404,595)**	**8.4 (6.9,10.1)**	**1435 (1009,1913)**	**12.9 (9.2,17.1)**	**54.9 (9.8,115.9)**
**Rwanda**	**399 (283,559)**	**13.3 (9.6,18.3)**	**886 (623,1216)**	**13.8 (10,18.8)**	**3.6 (−38.9,68.8)**
**Somalia**	**216 (147,299)**	**7.8 (5.5,10.5)**	**575 (389,798)**	**8.6 (5.9,11.6)**	**10.5 (−20.8,52.8)**
**South Sudan**	**180 (130,256)**	**7 (5.1,9.9)**	**370 (260,530)**	**8.7 (6.3,12)**	**24.3 (−15.6,74.1)**
**United Republic of Tanzania**	**1045 (842,1278)**	**9.7 (7.9,11.8)**	**3281 (2426,4359)**	**12.2 (9.3,16.1)**	**26.1 (−9.4,74.2)**
**Uganda**	**660 (481,878)**	**10.1 (7.5,13.3)**	**2481 (1830,3315)**	**15.5 (11.8,20.9)**	**53.2 (5.4,120.7)**
**Zambia**	**285 (207,393)**	**9.2 (6.9,12.3)**	**1385 (808,2155)**	**17 (11,24.8)**	**83.7 (3.7,217.3)**
**Central Sub−Saharan Africa**	**1880 (1348,2517)**	**7.8 (5.8,10)**	**6351 (4726,8350)**	**10.6 (8.1,13.8)**	**36.8 (−0.1,85.3)**
**Angola**	**301 (215,417)**	**6.8 (5.1,9)**	**1399 (950,1897)**	**10.5 (7.4,13.9)**	**53.6 (2.2,122.3)**
**Central African Republic**	**109 (78,148)**	**8.9 (6.7,11.3)**	**254 (176,351)**	**10.3 (7.5,13.5)**	**16.5 (−19.8,66.2)**
**Congo**	**138 (82,220)**	**12.5 (7.8,19.1)**	**470 (283,740)**	**15.2 (9.9,22.7)**	**21.4 (−14.2,81.2)**
**Democratic Republic of the Congo**	**1250 (875,1712)**	**7.4 (5.4,9.8)**	**3989 (2927,5503)**	**10.1 (7.5,13.8)**	**37.2 (−4.5,96.1)**
**Equatorial Guinea**	**17 (11,24)**	**8.3 (5.8,11.3)**	**82 (48,130)**	**14.2 (8.9,21.5)**	**72.3 (8,176.6)**
**Gabon**	**66 (47,88)**	**11.8 (8.6,15.4)**	**158 (108,217)**	**14.7 (10.3,19.7)**	**25 (−15.9,79.1)**

Table S4: Percentage Change in Disability−Adjusted Life Years (DALYs) Due to Breast Cancer and Age−Standardized Rates (ASRs) per 100,000 Population, 1990 and 2021<br>Percentage change in age−standardized rates (ASRs) per 100,000 population, by location<br>(Generated from data provided by http://ghdx.healthdata.org/gbd−results−tool)					
	1990		2021		Percentage change in the ASRs per 100,000
	No (95% UI)	ASRs per 100,000 (95% UI)	No (95% UI)	ASRs per 100,000 (95% UI)	
Global	11155717 (10557213,11791161)	265 (250.3,279.7)	20587794 (19311747,21942329)	239 (224.2,254.9)	−9.8 (−15.5,−3.5)
High−income North America	1558573 (1484000,1630441)	479.6 (457.6,500.7)	1573163 (1465407,1677968)	273.5 (256.2,290)	−43 (−45.2,−40.9)
Canada	135721 (126206,144763)	431 (401,459.3)	149647 (133555,165878)	241.1 (217.6,266.9)	−44.1 (−49.5,−38.2)
Greenland	198 (155,250)	466.5 (374.4,581.2)	187 (136,249)	254.2 (187.6,333.5)	−45.5 (−61.9,−22.2)
United States of America	1422618 (1354616,1488367)	485.1 (462.9,507)	1423304 (1324514,1521177)	277.4 (260.1,294.8)	−42.8 (−45.2,−40.7)
Australasia	95091 (89914,100225)	422.8 (399.8,445.1)	113391 (101186,126328)	244.6 (220.6,271.2)	−42.1 (−47.2,−36.5)
Australia	75287 (71166,79796)	400.7 (378.4,424.7)	92238 (81476,102663)	235.6 (210.6,262.3)	−41.2 (−46.8,−34.6)
New Zealand	19804 (18504,21282)	535.3 (500.6,574.9)	21153 (19078,23158)	290.5 (265,317.3)	−45.7 (−50.3,−40.1)
High−income Asia Pacific	284243 (272459,297458)	137.5 (131.6,143.9)	542013 (483094,587974)	163.1 (149.6,175.5)	18.6 (10.5,25.1)
Brunei Darussalam	426 (313,560)	274.3 (202.8,364.6)	1432 (1106,1759)	305 (236.8,373.6)	11.2 (−21.7,57)
Japan	236171 (225680,245700)	142.5 (136.2,148.3)	427325 (378827,464672)	180.4 (166.4,192.1)	26.6 (20.4,31.7)
Singapore	7165 (6706,7654)	258.1 (241.6,275.2)	15115 (13899,16516)	174.4 (160.1,190.4)	−32.4 (−38,−25.8)
Republic of Korea	40481 (35639,47946)	103.6 (91.7,122.6)	98140 (79567,116750)	118.5 (96.4,140.4)	14.3 (−13.2,40.1)
Western Europe	2436430 (2335457,2524094)	472 (454,488.7)	2147410 (1938239,2311982)	274.3 (252.5,293.5)	−41.9 (−44.5,−39.4)
Andorra	210 (147,305)	354.6 (250.2,512.7)	387 (268,537)	262.2 (179.8,365.3)	−26.1 (−55.1,18.7)
Austria	47745 (44586,50829)	452 (422.4,480.7)	38255 (34043,42264)	239.2 (216.3,262.8)	−47.1 (−52.4,−41.3)
Belgium	76827 (71763,81361)	560.5 (525.5,595.8)	58638 (51945,64466)	292 (263.3,318.8)	−47.9 (−52.4,−42.8)
Cyprus	2918 (2430,3509)	369.9 (308.6,445.6)	5672 (4658,6893)	297.4 (244,358.4)	−19.6 (−37.9,2.2)
Denmark	44737 (42265,47076)	631.4 (598.2,665.7)	28191 (25304,31143)	269.3 (244.1,297)	−57.3 (−61.3,−53.1)
Finland	25310 (23809,26957)	382.2 (358.9,407.6)	23908 (21095,26689)	233.1 (209.2,257.6)	−39 (−45.4,−31.9)
France	331485 (312245,351488)	448.5 (424.6,474.6)	348100 (305716,384214)	301.6 (269.4,332)	−32.7 (−38.2,−26.4)
Germany	541474 (510763,575157)	471.1 (443.3,499.3)	472838 (424407,515279)	294.2 (269,319.4)	−37.6 (−42.3,−32.7)
Greece	56428 (52996,59606)	406.3 (382.6,429.6)	63042 (56950,68722)	318.2 (292.9,344.8)	−21.7 (−27.2,−15.4)
Iceland	1122 (1030,1208)	422.2 (387.7,454.6)	1305 (1138,1468)	253.1 (223.1,283.8)	−40.1 (−47.3,−32.5)
Ireland	19646 (18548,20958)	525.3 (496.5,558.8)	19239 (17120,21402)	266.3 (239.1,296.3)	−49.3 (−54.6,−43.5)
Israel	22231 (20816,23741)	486.2 (456.2,517.3)	30644 (26915,33998)	270 (240.6,297.7)	−44.5 (−49.9,−38.5)
Italy	363124 (345048,377429)	459.8 (438.5,477.9)	329258 (291428,357189)	275.5 (251.1,296.3)	−40.1 (−43.3,−36.9)
Luxembourg	2656 (2494,2827)	520.7 (487.5,553.8)	2484 (2225,2761)	247.2 (221.2,274)	−52.5 (−57.9,−46.5)
Malta	2285 (2121,2468)	537 (498.6,581.5)	2391 (2119,2699)	304.6 (271.3,340.6)	−43.3 (−49.6,−35.6)
Monaco	296 (219,392)	537.3 (402.2,709.1)	412 (309,545)	557.2 (408.8,749.5)	3.7 (−27.7,60.6)
Netherlands	99079 (92685,105670)	532.4 (499.5,566.8)	89464 (80916,98654)	299.3 (274.6,327.2)	−43.8 (−48.4,−38.8)
Norway	21383 (20253,22411)	372.6 (355.6,390.1)	17174 (15541,18583)	194.6 (178.4,210)	−47.8 (−51,−44.1)
Portugal	52478 (49444,56001)	421.4 (396.3,449.6)	49829 (44446,54115)	255.8 (231.9,278.3)	−39.3 (−44.6,−33.6)
San Marino	96 (76,121)	302 (238.5,380.6)	107 (64,156)	176.5 (104.3,262.6)	−41.6 (−67,−7.1)
Spain	191296 (178609,204006)	397.6 (373,422.8)	175277 (155218,193139)	213.3 (193.2,234.2)	−46.4 (−50.9,−41.6)
Sweden	43938 (40764,47201)	355.1 (331.2,381.2)	35506 (29840,40845)	194.7 (164.2,224.1)	−45.2 (−53.6,−37.2)
Switzerland	37250 (34673,39682)	396.8 (370.4,422.1)	33550 (29181,37008)	207.7 (184.8,227.8)	−47.7 (−52.2,−42.7)
United Kingdom	450411 (434224,464322)	577.8 (559.4,594.4)	319847 (295808,338426)	286.3 (269.2,301.5)	−50.4 (−52.1,−48.9)
Southern Latin America	204808 (194621,214130)	441.1 (418.2,461.2)	263439 (242474,283127)	317.7 (292.7,341.5)	−28 (−33.7,−22)
Uruguay	20845 (19652,22000)	579.1 (547.4,610.4)	22126 (20082,23965)	456.3 (417.5,492.3)	−21.2 (−28.8,−13.1)
Argentina	154536 (145347,162524)	480.9 (452.9,505.9)	193734 (177260,209187)	361.8 (331.2,391.2)	−24.8 (−31.2,−17.2)
Chile	29417 (27527,31343)	274.7 (257.5,292.8)	47565 (43018,52085)	194.6 (176.4,213.1)	−29.1 (−36.2,−21.1)
Eastern Europe	944244 (912789,974547)	347.4 (336.3,358.6)	990240 (884095,1122979)	304.4 (270.9,346.4)	−12.4 (−22.4,−1.2)
Belarus	39730 (35956,43870)	319.9 (289.1,353.7)	38580 (30129,48618)	259.9 (201.2,330.2)	−18.8 (−38.3,7)
Estonia	7362 (6756,8003)	373.7 (342.9,407.1)	5605 (4582,6638)	239.9 (197.2,283.6)	−35.8 (−47.4,−22.7)
Latvia	12585 (11132,14280)	369.2 (326.6,419.2)	9988 (8152,11845)	295.1 (239.2,351)	−20.1 (−35.1,−2.1)
Lithuania	14470 (13405,15588)	333.3 (310.6,358.5)	13804 (11518,16140)	286 (239.7,333.1)	−14.2 (−28.9,1.4)
Republic of Moldova	17891 (15880,19889)	390.8 (347,434.4)	16988 (14385,20249)	298.8 (252.7,356)	−23.5 (−37.8,−6.4)
Russian Federation	551463 (535942,567038)	310.7 (302,319.5)	691641 (615062,765742)	309.6 (274.2,342.8)	−0.4 (−11.5,9.7)
Ukraine	300743 (279768,323637)	445.9 (415.4,477.9)	213634 (136445,314989)	302.5 (190.2,453)	−32.2 (−56.9,−0.3)
Central Europe	520818 (499021,544125)	355.7 (340.8,371.3)	609675 (562395,659598)	308.6 (284,335.3)	−13.2 (−20.4,−5.3)
Albania	3414 (2676,4291)	144.8 (113.7,181.8)	6214 (4457,8276)	162.9 (116.7,216.6)	12.4 (−26.7,68.9)
Bosnia and Herzegovina	10575 (9049,12004)	232.4 (200.4,261.7)	14666 (11357,18271)	257.5 (197.2,322.9)	10.8 (−18.4,45)
Bulgaria	41670 (37127,47288)	361.3 (322.5,408.4)	45209 (37039,54029)	376.4 (308.6,450.5)	4.2 (−18.5,31.6)
Croatia	23090 (20296,25881)	381.6 (336.7,426.7)	21816 (18662,25156)	279.1 (234.9,319)	−26.8 (−40.3,−12.7)
Czechia	51224 (46131,56974)	388.6 (350.4,432)	45747 (38345,53800)	239.8 (200.5,282.1)	−38.3 (−49.9,−23.7)
Hungary	60399 (53737,67867)	439.9 (390.1,494.8)	53972 (46084,62972)	315.7 (267.7,370.5)	−28.2 (−41.2,−13.9)
Montenegro	2457 (1906,3175)	380.9 (295.6,492)	3626 (2849,4571)	397.3 (311.6,498.4)	4.3 (−25.4,47.6)
North Macedonia	7820 (6413,9376)	386.1 (316.3,463.2)	11545 (8924,14620)	361.4 (279.1,459.1)	−6.4 (−29.9,25.8)
Poland	148064 (143026,153090)	346.6 (334.7,358.6)	199597 (174995,222959)	307.9 (270.1,344.2)	−11.1 (−21.6,−1.8)
Romania	84795 (79089,91590)	309.9 (289.8,332.8)	104209 (90634,117851)	320.9 (279.3,361.9)	3.5 (−11.3,20)
Serbia	49478 (37467,62651)	437.1 (331.4,554.5)	58771 (44545,75040)	398.7 (302.4,508.7)	−8.8 (−36.5,28.4)
Slovakia	20662 (18029,24015)	356.1 (310.7,413.4)	26469 (20436,33169)	297.9 (232.7,369.8)	−16.3 (−38.2,8.7)
Slovenia	8840 (8110,9658)	366 (334.9,400.4)	8960 (7389,10768)	221.8 (182.5,267.4)	−39.4 (−50.6,−26.4)

Central Asia	172555 (163073,181562)	340.6 (321.5,358.4)	219023 (194658,246202)	236.1 (210.6,264)	−30.7 (−38.9,−21.9)
Armenia	15440 (14404,16421)	515 (480.2,548.8)	13332 (11709,15273)	315.3 (277.3,360.9)	−38.8 (−47.5,−28.9)
Azerbaijan	17506 (14528,20087)	316.3 (261.4,361.2)	29591 (21892,38105)	245.6 (183.1,313.2)	−22.4 (−43,−0.9)
Georgia	30962 (27691,34403)	504.5 (450.9,559.2)	25278 (21661,29320)	467.7 (400.2,543.7)	−7.3 (−23.6,9)
Kazakhstan	53695 (47814,59879)	389.4 (345.6,434.5)	44248 (37008,51719)	225.4 (188.8,263)	−42.1 (−52.5,−29.7)
Kyrgyzstan	10060 (8913,11325)	321.7 (286.1,361.2)	11520 (9343,14009)	200.7 (164.1,242.4)	−37.6 (−49.5,−21.4)
Mongolia	1140 (886,1422)	94.6 (73.9,118.8)	3030 (2379,3712)	101.9 (79.1,125)	7.6 (−23.9,48.1)
Tajikistan	7570 (6064,9158)	248.5 (199.2,300.5)	13974 (8437,21127)	180 (113.2,265.7)	−27.6 (−55.4,9.7)
Turkmenistan	5506 (4800,6258)	248.7 (217.6,282.3)	10997 (8153,14820)	227.2 (169.1,304.6)	−8.6 (−32.7,24.3)
Uzbekistan	30676 (27693,33622)	243.6 (218.6,268.2)	67052 (55460,80385)	205 (170.5,246)	−15.9 (−30.6,2.8)
Central Latin America	195065 (189666,200866)	195.6 (190,201.6)	635311 (550185,716850)	241.7 (209.7,272.7)	23.5 (7.5,39.7)
Colombia	48394 (45195,51814)	231.3 (216.1,247.5)	134373 (111717,162045)	244.7 (203.6,295.3)	5.8 (−13.1,28.7)
Costa Rica	3918 (3623,4231)	199.8 (184.8,215)	15199 (13327,17320)	276.9 (243,315.5)	38.6 (19.3,60.1)
El Salvador	4523 (3900,5196)	137.7 (119.6,157.5)	13524 (10715,17242)	223.1 (176.8,284.4)	62 (22.6,114.1)
Guatemala	4069 (3797,4330)	94.7 (88.6,100.6)	18598 (15485,21744)	151.8 (126.5,177.4)	60.3 (34,88.1)
Honduras	3239 (2421,4102)	131.7 (95.7,169.4)	15609 (10943,21635)	214.9 (153,296.6)	63.1 (9.7,137.6)
Mexico	98082 (95049,100753)	190.7 (185,196)	314497 (261211,372369)	231.7 (193.1,273.7)	21.5 (0.7,43.1)
Nicaragua	2066 (1763,2391)	106.6 (90.9,123.8)	8525 (6601,10723)	153.2 (119,192)	43.7 (12.4,87.4)
Panama	2977 (2753,3212)	179.4 (166.1,193.1)	10677 (8380,12962)	242 (190.1,293.7)	34.9 (5.1,65.1)
Venezuela (Bolivarian Republic of)	27797 (26164,29480)	235.8 (222.2,250.2)	104309 (78943,134467)	335.8 (254.6,432.7)	42.4 (7.1,88.3)
Andean Latin America	46690 (39607,54552)	195.2 (166.8,227.9)	129278 (102416,162983)	206.6 (163.9,259.5)	5.8 (−15.1,31.7)
Bolivia (Plurinational State of)	9605 (6232,13922)	254.2 (166.6,367.3)	28407 (18770,40725)	280.5 (185.7,402.7)	10.4 (−25.9,64.7)
Ecuador	9010 (8430,9593)	144.5 (135.1,153.6)	33958 (25850,43346)	200.3 (152.9,255.2)	38.6 (4.6,77.1)
Peru	28075 (22920,33271)	201.8 (165.9,240)	66912 (48493,89219)	189 (137.2,250.8)	−6.4 (−34.4,31.6)
Caribbean	83121 (75705,92068)	304.9 (278.3,337.1)	166471 (139014,197384)	314.1 (261.8,372.9)	3 (−11.5,18.9)
Antigua and Barbuda	212 (192,236)	420.2 (378.2,466.4)	515 (479,556)	462.5 (431.4,498.4)	10.1 (−2.8,24.6)
Barbados	1323 (1212,1428)	522.8 (478.2,564.6)	2502 (2001,3117)	538.3 (427.7,675.7)	3 (−19.3,30.4)
Belize	147 (133,161)	148.1 (134.5,161.8)	753 (660,857)	212.2 (186.3,241.1)	43.3 (22.1,68.1)
Bermuda	354 (316,395)	551.1 (494.1,614.7)	357 (296,449)	304 (250.4,380.3)	−44.8 (−55.6,−30)
Bahamas	1110 (1015,1216)	606.6 (552.7,664.8)	2771 (2218,3510)	626.9 (503.8,788.6)	3.4 (−19.5,31.2)
Cuba	30435 (28378,32450)	296.1 (275.8,315.5)	47783 (40463,56792)	262.4 (222.2,310.6)	−11.4 (−25.5,5.8)
Dominica	251 (215,288)	445.5 (382.8,511.7)	369 (279,466)	451.2 (341.3,566.3)	1.3 (−24.1,34.2)
Dominican Republic	8218 (6912,9657)	187.7 (158.3,219.7)	23309 (17733,30651)	220.3 (167.8,289)	17.4 (−14.8,63.3)
Grenada	289 (260,321)	452.4 (405.8,504.5)	548 (472,629)	471.9 (407.9,537.5)	4.3 (−12.2,23.8)
Guyana	1491 (1286,1724)	328.9 (286.6,377.5)	3036 (2248,4005)	422.2 (314.8,554.3)	28.4 (−9.5,70.9)
Haiti	13861 (8235,21806)	356.1 (216.1,551.8)	38422 (23583,60282)	411 (254.2,638)	15.4 (−25.2,83.5)
Jamaica	5326 (4943,5765)	315.5 (292.8,341.6)	13802 (10370,17670)	448.2 (337,574.3)	42 (6.3,86.1)
Puerto Rico	11327 (10519,12066)	319.6 (296.7,340.4)	14787 (12299,17399)	264.4 (218.4,312.6)	−17.3 (−31.2,−0.8)
Saint Kitts and Nevis	226 (206,246)	681.2 (621.3,748)	311 (253,377)	420.5 (343.3,506)	−38.3 (−49.9,−24.7)
Saint Lucia	427 (395,463)	488.7 (450.9,529.2)	861 (713,1049)	362.3 (300.3,441.6)	−25.9 (−40.6,−8.7)
Saint Vincent and the Grenadines	338 (302,369)	481.5 (431.6,527.4)	633 (546,731)	456.8 (393.8,527.8)	−5.1 (−18.7,13.5)
Suriname	758 (638,889)	267.6 (225.9,312.8)	1888 (1441,2414)	290.2 (221.7,370)	8.5 (−20.2,45.8)
Trinidad and Tobago	3746 (3461,4066)	422.1 (390.6,457.2)	7698 (5847,9883)	414.5 (315,534.5)	−1.8 (−25.8,27.4)
United States Virgin Islands	468 (391,556)	480.6 (403.9,575.6)	490 (340,692)	350.6 (243.4,494.9)	−27 (−50.7,7.4)
Tropical Latin America	284539 (274497,294271)	268.5 (258.3,278.3)	758445 (712292,797212)	287.8 (269.9,302.6)	7.2 (1.6,13.2)
Brazil	279531 (269434,289147)	270.2 (260,279.9)	740892 (697324,781531)	288.1 (271,303.9)	6.6 (1.3,12.5)
Paraguay	5008 (4025,6042)	199.1 (160,240.8)	17553 (13106,23341)	278.1 (208.5,370.6)	39.7 (−2.5,101.4)
East Asia	1554706 (1267291,1886528)	152.2 (125.2,184)	3185602 (2514948,3998849)	148.7 (117.2,186.7)	−2.3 (−28.3,34.4)
China	1490135 (1203531,1822017)	151.5 (123.5,184.4)	3027381 (2358954,3841593)	146.3 (113.8,185.5)	−3.4 (−29.8,35.1)
Democratic People's Republic of Korea	35127 (22958,51821)	183.7 (121.4,269.7)	69073 (46529,93982)	201.1 (137.3,273.3)	9.5 (−27.8,63.6)
Taiwan (Province of China)	29444 (27618,31181)	160.2 (150.6,169.5)	89147 (81415,96605)	232.8 (212.9,251.6)	45.3 (30.9,59.2)
Southeast Asia	736853 (613881,893841)	234 (195.3,283.4)	2251593 (1862150,2759997)	302.6 (250.7,369.4)	29.3 (9.2,53.6)
Cambodia	13656 (8564,20781)	244.7 (154.8,371.1)	51324 (36633,69407)	354.4 (253.9,474.9)	44.8 (−12.6,147.2)
Indonesia	293087 (203336,414026)	233.2 (161.4,329.2)	907646 (609120,1309603)	309.8 (208.6,445.4)	32.8 (−2.1,82.7)
Lao People's Democratic Republic	5840 (3337,9531)	237.3 (138.7,384.1)	17376 (12231,23897)	297.1 (209.7,409.1)	25.2 (−27.2,120.4)
Malaysia	38286 (32230,45157)	331.3 (278.6,387.6)	131263 (110556,155080)	417.9 (352.9,491.6)	26.2 (2,55.1)
Maldives	154 (77,271)	133.3 (70.2,227.3)	490 (365,638)	108.5 (82.4,138.4)	−18.6 (−58.9,74.2)
Mauritius	1620 (1508,1752)	194.3 (181.2,209.9)	6804 (6136,7299)	381.7 (343.1,410.7)	96.4 (71.8,117.1)
Myanmar	92088 (61697,133105)	321 (218.6,455.3)	185884 (138615,250133)	336.3 (252.9,451.8)	4.7 (−33.6,67.2)
Philippines	114009 (101355,125285)	305.4 (271.1,337.3)	393046 (305590,494761)	403.7 (316.4,504.6)	32.2 (1,69.2)
Seychelles	151 (129,175)	269.5 (230.1,311.3)	429 (363,501)	338.3 (287.1,396.3)	25.5 (1.2,56.4)
Sri Lanka	21827 (18245,26109)	168.4 (140.6,200.7)	53663 (34689,73470)	199.4 (129.1,271.8)	18.4 (−27.2,70.5)
Thailand	87261 (70569,104353)	194.5 (158.1,230.9)	286815 (214480,365927)	283.7 (213.7,363.4)	45.8 (3.5,105.8)
Timor−Leste	597 (371,919)	148 (94,225.6)	1991 (1358,2719)	214.2 (145.8,292.1)	44.7 (−6.3,136.9)
Viet Nam	67213 (51288,87682)	157 (120.3,205.2)	211722 (159070,286371)	191.1 (144,255.2)	21.7 (−14.9,74.4)
Oceania	12216 (9415,15460)	310.1 (244.3,391)	35955 (28930,45870)	352.8 (289.4,436.8)	13.8 (−12.1,48.4)
American Samoa	110 (90,135)	393.8 (324.6,472.7)	313 (244,388)	609.9 (478.4,757.1)	54.9 (13,105.8)
Cook Islands	77 (58,99)	553.5 (428.5,712.6)	135 (102,175)	579.8 (432.7,753.3)	4.7 (−30.1,50.3)
Micronesia (Federated States of)	228 (152,318)	410.6 (277.5,565.6)	459 (326,620)	526.6 (378.9,702.3)	28.3 (−12.8,95.7)
Fiji	2493 (1954,3161)	507.7 (403,641.8)	5083 (3707,6778)	595.2 (440.4,786.1)	17.2 (−18.3,62.9)
Guam	218 (186,255)	243.6 (209.2,283.4)	473 (396,565)	244.8 (204.7,289.9)	0.5 (−19.5,23)

Kiribati	179 (139,228)	393.7 (303.8,502.4)	477 (353,652)	540 (405.4,727.2)	37.2 (−0.7,102.8)
Marshall Islands	69 (48,98)	332 (233,470.4)	223 (132,354)	480.3 (289.8,744.8)	44.7 (−2.2,102.8)
Nauru	29 (17,46)	452 (270.5,700.9)	48 (28,75)	640.8 (381.9,994.5)	41.8 (2.2,94.4)
Niue	9 (7,12)	433.8 (326.5,564.7)	11 (8,14)	534.5 (402.2,705.8)	23.2 (−17.4,72.7)
Northern Mariana Islands	99 (69,135)	338 (249.5,446.1)	234 (191,275)	404.3 (330.5,470.2)	19.6 (−13.1,61.1)
Palau	68 (52,90)	615.3 (470.1,802.4)	129 (98,168)	564.7 (433.8,730.3)	−8.2 (−35.7,28.2)
Papua New Guinea	6722 (4539,9607)	264.7 (181.9,375.7)	23293 (16583,32190)	305.2 (221,416.4)	15.3 (−29.4,84.2)
Samoa	248 (184,322)	265.2 (198.1,342)	520 (377,697)	324.9 (236,432.8)	22.5 (−16,80.2)
Solomon Islands	354 (203,514)	203.7 (125.8,291.9)	1670 (1190,2325)	352.3 (250.1,480.7)	73 (9.2,192.4)
Tokelau	6 (4,8)	445.9 (288.2,645)	7 (5,10)	513.9 (383.9,681.3)	15.3 (−17.7,72.4)
Tonga	330 (262,421)	539.3 (429.8,681.9)	495 (357,661)	588 (427.5,782)	9 (−25,57.1)
Tuvalu	29 (19,43)	400.7 (262.4,584.6)	49 (35,67)	444.6 (320.2,611.1)	10.9 (−25.5,80.3)
Vanuatu	169 (113,243)	207.5 (142.5,294.3)	722 (515,954)	329.8 (240.1,429.7)	58.9 (9.6,134.9)
North Africa and Middle East	257146 (227823,297322)	124.1 (110.8,142.3)	1081482 (942159,1242362)	194.9 (170.8,223.5)	57 (36.1,81.5)
Afghanistan	11867 (5876,20760)	163 (81.2,284.4)	37374 (18198,69508)	244.7 (129.6,424.6)	50.1 (−0.1,115.8)
Algeria	15870 (11964,20691)	114.9 (87.1,149.6)	53573 (40114,70769)	128.9 (97.5,167.9)	12.2 (−18.4,52.9)
Bahrain	822 (692,966)	321.7 (270.3,378.5)	3646 (2840,4724)	287.4 (225.4,369.1)	−10.7 (−35.5,22)
Egypt	54595 (46655,65465)	148.9 (128.7,177.7)	212091 (166018,265208)	268.9 (214.1,332.6)	80.7 (36.3,136)
Iran (Islamic Republic of)	36260 (31133,41910)	111 (95.4,128.1)	154194 (138241,172179)	164.5 (147.6,183.6)	48.2 (23.2,79.3)
Iraq	19678 (14903,25836)	205.8 (154.2,271.5)	86847 (61317,117329)	275.7 (195.3,367.2)	33.9 (−11.4,96.1)
Jordan	4146 (3133,5433)	230.7 (176.3,296.6)	22542 (15911,30485)	233.8 (167,315.5)	1.4 (−37.8,58.2)
Kuwait	1309 (1168,1456)	140.8 (125.5,155.8)	6741 (5652,7946)	141.2 (117.1,163.5)	0.3 (−17.4,19.5)
Lebanon	7053 (5022,9514)	299.1 (214.8,402.4)	19763 (15842,24140)	334.8 (268.3,409.5)	11.9 (−26,67.9)
Libya	2889 (2259,3785)	128.7 (101.4,166.7)	14619 (10650,20600)	209.3 (155.4,288.6)	62.7 (6.5,145.3)
Morocco	16289 (12307,21712)	98.7 (76,129.2)	62991 (41639,93221)	163.9 (109.6,239.9)	66 (12.9,145.1)
Oman	491 (349,678)	56.2 (40.9,75.7)	1752 (1318,2318)	61.6 (47.3,78.2)	9.7 (−23.8,65.6)
Palestine	2981 (2108,4169)	304.7 (217.7,423.9)	10568 (8574,12953)	331.6 (267.8,405.5)	8.8 (−24.8,61.5)
Qatar	507 (395,629)	232.1 (185.4,282.6)	3392 (2396,4703)	216.9 (160.3,290.7)	−6.5 (−36.7,33.9)
Saudi Arabia	8141 (5838,11227)	92.4 (67.4,124.9)	53419 (37337,74869)	139.8 (103.4,189.2)	51.4 (−0.1,132.6)
Sudan	10355 (6277,16371)	91.7 (57.2,141.5)	37310 (21748,60294)	134.4 (82.3,213.9)	46.4 (−11.8,137)
Syrian Arab Republic	9689 (7141,12376)	147.1 (110.1,186)	27819 (20140,37642)	185.7 (136.8,249.2)	26.3 (−16.4,101.2)
Tunisia	8367 (6848,10117)	150.2 (123.1,181.5)	26080 (18412,35802)	186.9 (132.4,255.5)	24.4 (−15,79.2)
Turkey	39253 (31386,48305)	96.2 (77.2,117.8)	207351 (163090,257325)	214.1 (168.3,265.3)	122.6 (62.2,207.9)
United Arab Emirates	1689 (1197,2312)	235.5 (178.7,304.5)	13207 (9601,17670)	220.4 (161.4,288.6)	−6.4 (−37.3,31.9)
Yemen	4753 (2846,6809)	77 (48.2,108.3)	25193 (16939,36602)	128.9 (89.7,184.2)	67.4 (10.1,161.5)
South Asia	1117449 (992472,1259997)	152.4 (135.3,172.2)	3756986 (3282346,4303559)	223.5 (195.2,255.9)	46.6 (25.4,72.4)
Bangladesh	67940 (50384,92843)	108.2 (81,147.2)	232549 (173726,300457)	148.1 (110.9,190.5)	36.8 (−5.9,109.4)
Bhutan	387 (261,514)	119.8 (83.4,157.9)	915 (627,1266)	133 (91.8,182.7)	11 (−27.7,68.9)
India	839332 (720251,979732)	140.6 (119.8,164.3)	2747451 (2327631,3246429)	206.2 (175.1,243.9)	46.6 (23.2,75.4)
Nepal	14654 (10836,19350)	122.8 (90.9,160.4)	40206 (28720,55615)	154 (110.4,212.4)	25.4 (−14.5,91.2)
Pakistan	195136 (154890,240959)	297.7 (235.9,368.7)	735864 (524353,988805)	457.5 (328.9,608.3)	53.7 (6.8,115.8)
Southern Sub−Saharan Africa	91179 (77304,105536)	286.6 (239.9,333.7)	271340 (242374,302795)	412.6 (370.9,457.5)	44 (20.3,75.9)
Botswana	1814 (1220,2631)	275.5 (189.4,390.1)	6041 (4028,9026)	334.2 (236.4,479)	21.3 (−18.8,97.1)
Lesotho	2042 (1404,2885)	223.6 (154.9,314.9)	5404 (3371,7938)	443.9 (280.7,646.6)	98.5 (7.8,263.6)
Namibia	2274 (1868,2807)	302.8 (250.2,373.2)	8930 (5675,12865)	535.9 (346.9,752.2)	77 (12.6,152.1)
South Africa	72512 (59555,85951)	297.1 (240.5,354.4)	202754 (181212,226881)	393.5 (354.8,436.6)	32.5 (8.6,67.2)
Eswatini	958 (719,1229)	272.8 (207.3,348.1)	3042 (1703,4827)	442.2 (254.8,689.6)	62.1 (−6.5,171.6)
Zimbabwe	11579 (8926,15208)	246.3 (190.4,319.2)	45169 (32021,61948)	505 (363.6,681.5)	105 (41.2,207)
Western Sub−Saharan Africa	246939 (200577,294707)	241.4 (197.7,286.1)	889176 (667297,1156567)	356.6 (276.5,455.5)	47.7 (13.4,97.1)
Benin	4125 (3317,5003)	181.9 (147.2,217.6)	13741 (9767,18595)	209.7 (153.4,278.8)	15.3 (−17.5,62.1)
Burkina Faso	15697 (11973,20105)	315.4 (241.5,399.8)	40009 (27609,54884)	342.6 (246.6,457.4)	8.6 (−23.9,51)
Cameroon	12696 (10092,15709)	234.4 (187,290.7)	46916 (33166,65623)	281.7 (201.4,387.9)	20.2 (−16.8,74.6)
Cabo Verde	536 (430,661)	260.9 (206.2,324.2)	1090 (837,1390)	220.8 (170.5,281.3)	−15.4 (−40.4,19.1)
Chad	4663 (3327,6158)	150.2 (108.5,197.8)	14056 (9757,18996)	184.8 (132,245.6)	23.1 (−16.8,83.3)
CÃ´te d'Ivoire	12962 (9826,16891)	243.3 (188.8,309.8)	48062 (34144,66364)	320.9 (233.6,431.1)	31.9 (−7.7,90.9)
Gambia	396 (288,536)	91.6 (67.8,122)	1892 (1354,2580)	155.2 (112.4,208.6)	69.4 (16.6,151)
Ghana	22689 (17354,29088)	280.3 (217.2,355.3)	71300 (52507,96485)	330.7 (246.7,440)	18 (−18.8,74.9)
Guinea	7177 (5386,9074)	197.2 (148.2,247.9)	18343 (12557,26129)	263.5 (184.5,368.8)	33.6 (−11.3,101.1)
Guinea−Bissau	1285 (884,1878)	256 (177,374.9)	3553 (2464,4922)	338.5 (233.9,463.2)	32.2 (−10.7,98.9)
Liberia	2159 (1646,2731)	168.1 (130.8,209.4)	7144 (4826,10624)	235.4 (163.5,335.4)	40.1 (−11.3,115)
Mali	11032 (8992,13375)	229.8 (187.3,277.6)	28944 (20296,40448)	258.4 (183.4,354.2)	12.5 (−19.4,56.7)
Mauritania	2462 (1745,3336)	224.7 (162.3,305.3)	6857 (5151,9204)	272.3 (206,364.5)	21.2 (−20.6,89.8)
Niger	4699 (3445,6242)	129.5 (97.3,174)	15199 (10340,21292)	147.9 (101.8,205.1)	14.2 (−19.2,60.5)
Nigeria	130329 (96177,171578)	261.2 (195.5,342.6)	521767 (346315,761196)	453 (310.1,647.3)	73.4 (17.1,164.8)
Sao Tome and Principe	116 (94,144)	174.4 (140.7,213.8)	376 (274,498)	263.5 (197.4,345)	51.1 (7.4,113.7)
Senegal	6843 (5298,8618)	180 (140.8,225.4)	24281 (18085,33109)	261.3 (196.6,351)	45.1 (3.7,102.9)
Sierra Leone	3521 (2416,4753)	152.7 (106.9,205.8)	11000 (7556,14938)	226 (160.3,303.1)	48 (2.4,114.1)
Togo	3542 (2811,4501)	215.5 (175.2,271.2)	14635 (10164,20077)	289.2 (207,396.5)	34.2 (−5.8,91.5)
Eastern Sub−Saharan Africa	246741 (204152,301215)	268.2 (224.6,324.8)	742606 (622572,889228)	335.9 (286.4,392.2)	25.2 (−2.8,63)
Burundi	8010 (5612,11513)	294.1 (208.3,418.3)	15616 (11144,21694)	238.2 (173.8,327.5)	−19 (−50.2,31.3)
Comoros	683 (483,931)	285.2 (204,387.6)	2191 (1627,2964)	379.9 (283.1,507.2)	33.2 (−8.6,102.9)
Djibouti	545 (387,739)	283.2 (209.9,376.8)	2944 (1914,4409)	335.5 (226.5,486.4)	18.5 (−20.5,80)
Eritrea	5147 (3726,7129)	322.7 (238.3,434.6)	15641 (10793,21740)	417.2 (294,569.8)	29.3 (−8.6,86.3)
Ethiopia	73547 (47516,110564)	297.7 (199,437.6)	165035 (132736,203405)	292.5 (238.4,360.3)	−1.8 (−36.6,57.8)
Kenya	19603 (13884,26798)	195.5 (140.5,265.4)	96615 (65988,134055)	328.6 (230.6,451.2)	68.1 (22.4,130.9)

**Madagascar**	**16590 (12909,21239)**	**269 (213.5,336.3)**	**47515 (33476,65004)**	**295.9 (211.2,393.6)**	**10 (−23.9,57.6)**
**Malawi**	**10300 (7919,12823)**	**219 (170.9,270.1)**	**31192 (22251,41635)**	**325.1 (235.2,432.3)**	**48.5 (4.9,104.2)**
**Mozambique**	**16539 (13405,20594)**	**230.1 (189.5,281.9)**	**49946 (34199,67734)**	**347.4 (244.2,464.1)**	**51 (2.6,115.4)**
**Rwanda**	**14205 (9790,20102)**	**405.9 (283.1,575)**	**30050 (20627,41575)**	**381.4 (267.1,522.6)**	**−6 (−46.5,58.4)**
**Somalia**	**8073 (5415,11322)**	**230.8 (158.2,316.5)**	**21094 (13855,29910)**	**244.6 (166.4,336.4)**	**6 (−26.8,51.4)**
**South Sudan**	**5908 (4116,8352)**	**202.4 (143.4,287.9)**	**13144 (9104,19104)**	**253 (177.7,361.4)**	**25 (−19.5,85.7)**
**United Republic of Tanzania**	**35547 (28282,43701)**	**278.1 (224,338.7)**	**112054 (81827,149230)**	**345.1 (255,459.7)**	**24.1 (−11.5,78.8)**
**Uganda**	**21447 (15396,29300)**	**286.3 (207.5,385.3)**	**86025 (61097,116421)**	**448.6 (330.1,600.8)**	**56.7 (6.5,134.5)**
**Zambia**	**10421 (7362,14515)**	**281 (202.8,387.8)**	**52898 (28925,85854)**	**514 (301.9,792.1)**	**82.9 (−8.8,238.2)**
**Central Sub−Saharan Africa**	**66310 (47209,89807)**	**235.7 (170.7,313.5)**	**225194 (167049,297292)**	**309.5 (231.8,406.9)**	**31.3 (−6.2,82.7)**
**Angola**	**11005 (7787,15574)**	**210 (151.2,289.2)**	**51061 (34881,69463)**	**314.5 (216.3,423.5)**	**49.8 (−3.5,123.7)**
**Central African Republic**	**3885 (2717,5377)**	**268.2 (192.8,361.4)**	**9289 (6244,13138)**	**304.5 (213.6,416.9)**	**13.5 (−23,68.5)**
**Congo**	**4745 (2744,7701)**	**377.6 (224,604.8)**	**17154 (10121,27402)**	**458.6 (280.4,717.5)**	**21.5 (−17.7,91.2)**
**Democratic Republic of the Congo**	**44005 (30649,60281)**	**226.1 (159.9,306.1)**	**139446 (103060,192192)**	**292.1 (215.3,402.6)**	**29.2 (−12.2,91.7)**
**Equatorial Guinea**	**592 (389,868)**	**252.8 (170,361.3)**	**2964 (1693,4795)**	**415.7 (247,652.1)**	**64.4 (−1.5,174.5)**
**Gabon**	**2078 (1473,2810)**	**342.9 (243.9,460.3)**	**5282 (3534,7453)**	**418 (285.4,576.5)**	**21.9 (−22.1,87.3)**

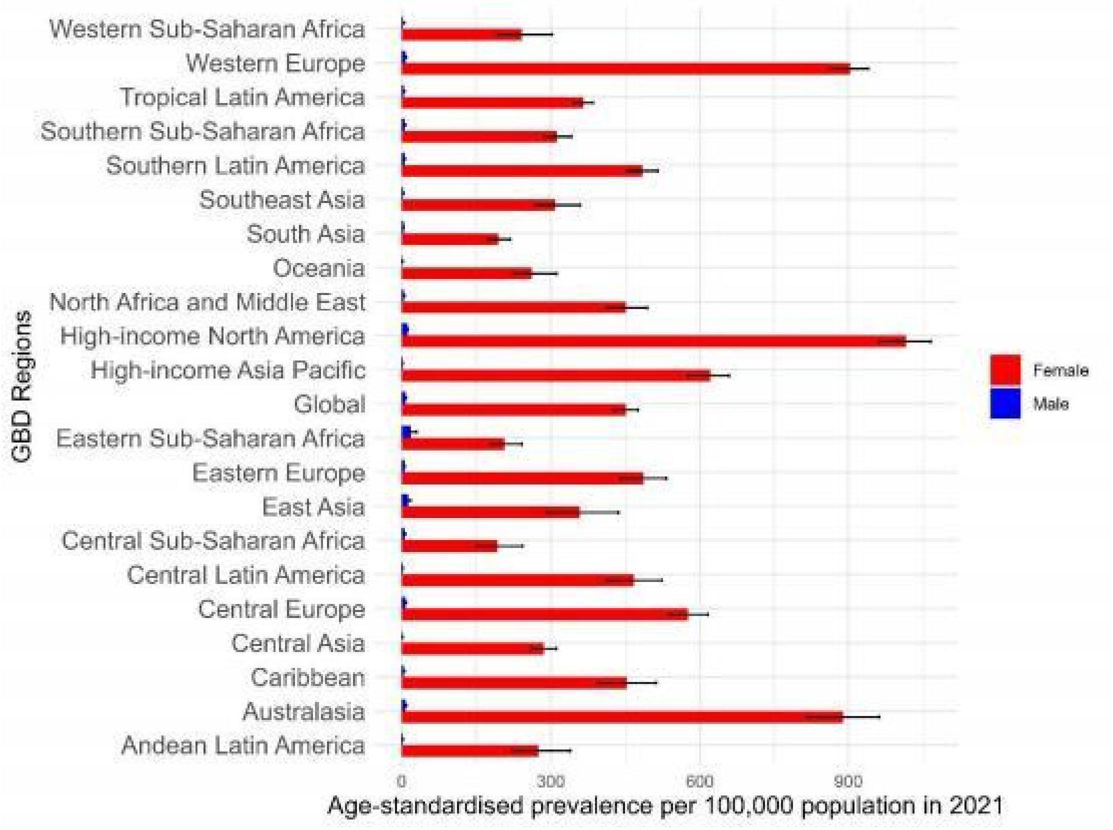

Figure S1: Age−standardized point prevalence of breast cancer by sex for all regions in the Global Burden of Disease Study, 2021

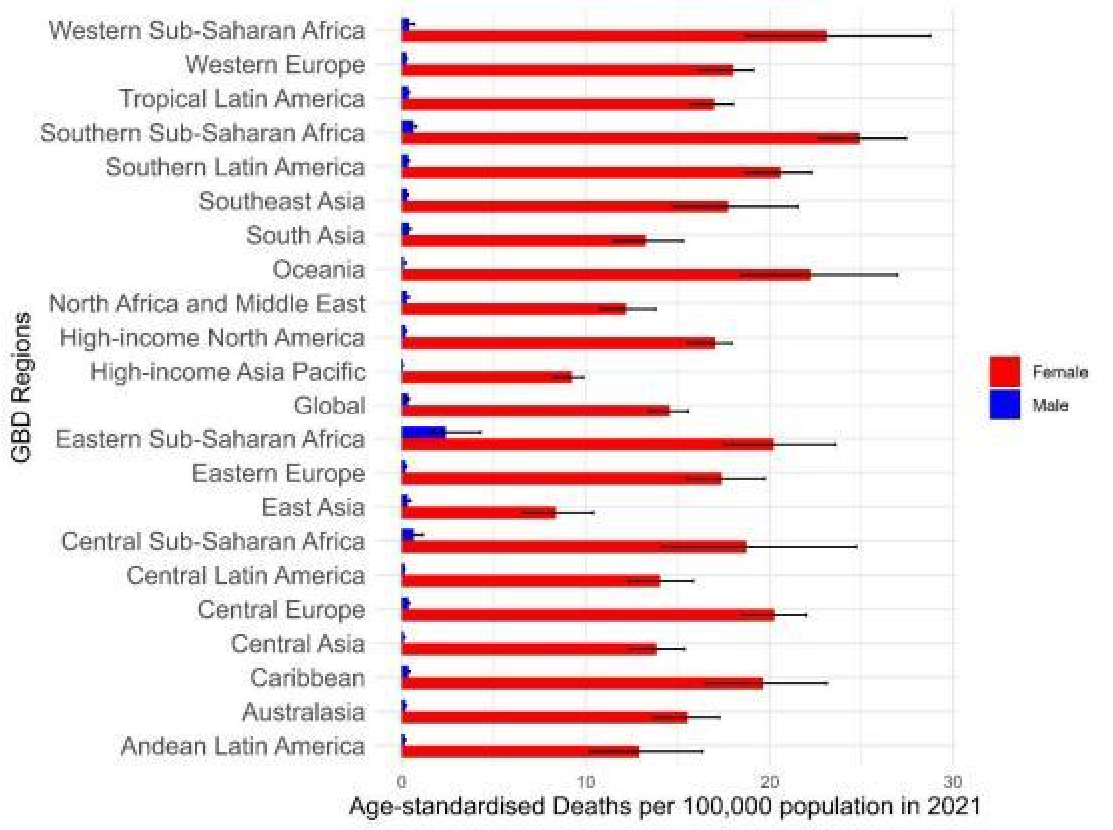

Figure S2: Age−standardized point mortality rates for breast cancer by sex for all regions in the Global Burden of Disease Study, 2021

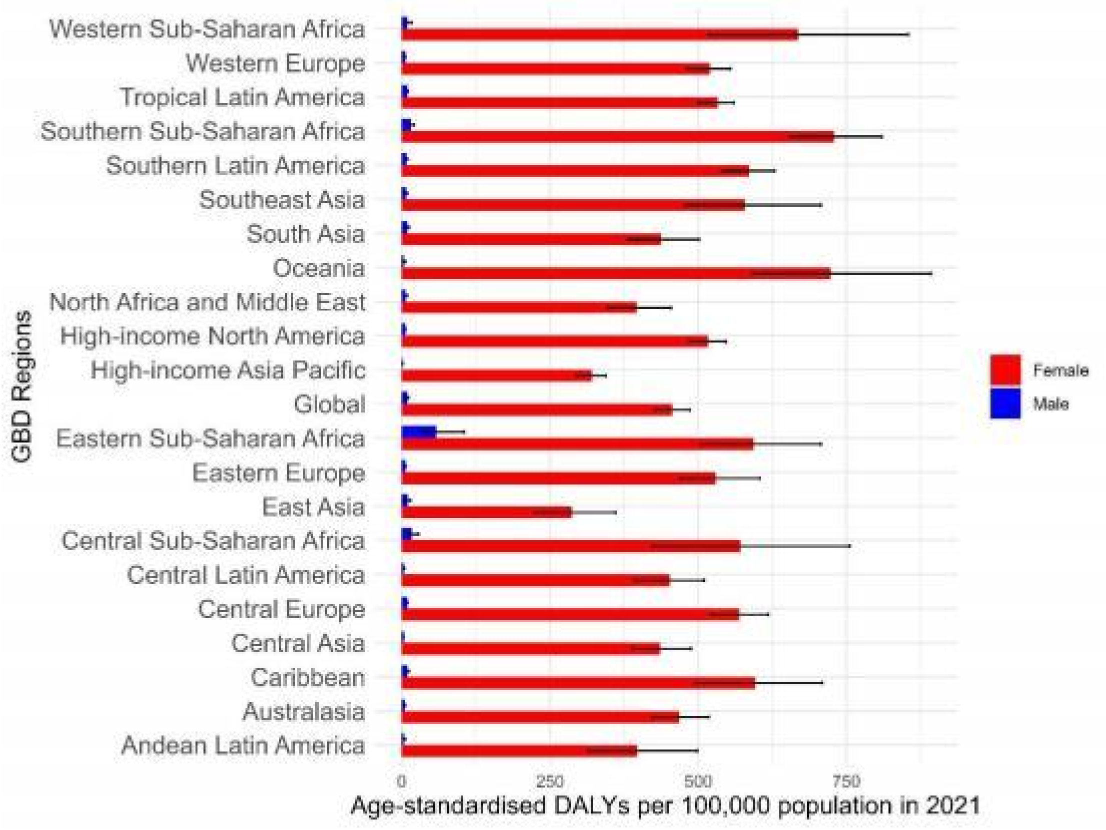

Figure S3: Age−standardized point DALY rates for breast cancer by sex for all regions in the Global Burden of Disease Study, 2021

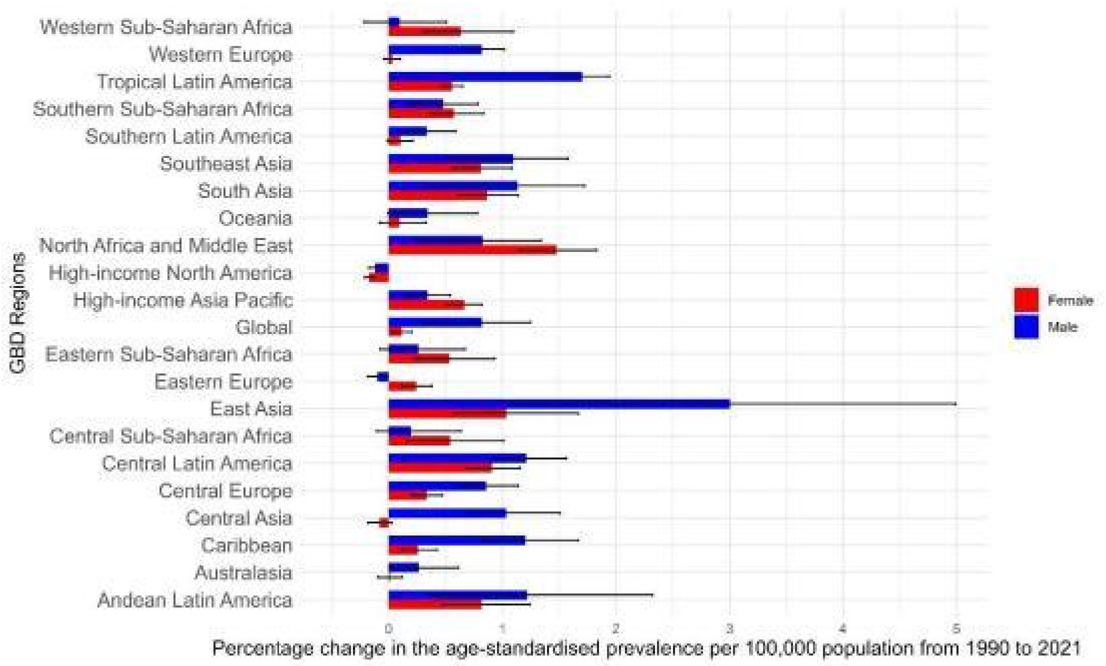

Figure S4: Percentage change in age−standardized point prevalence of breast cancer by sex from 1990 to 2021

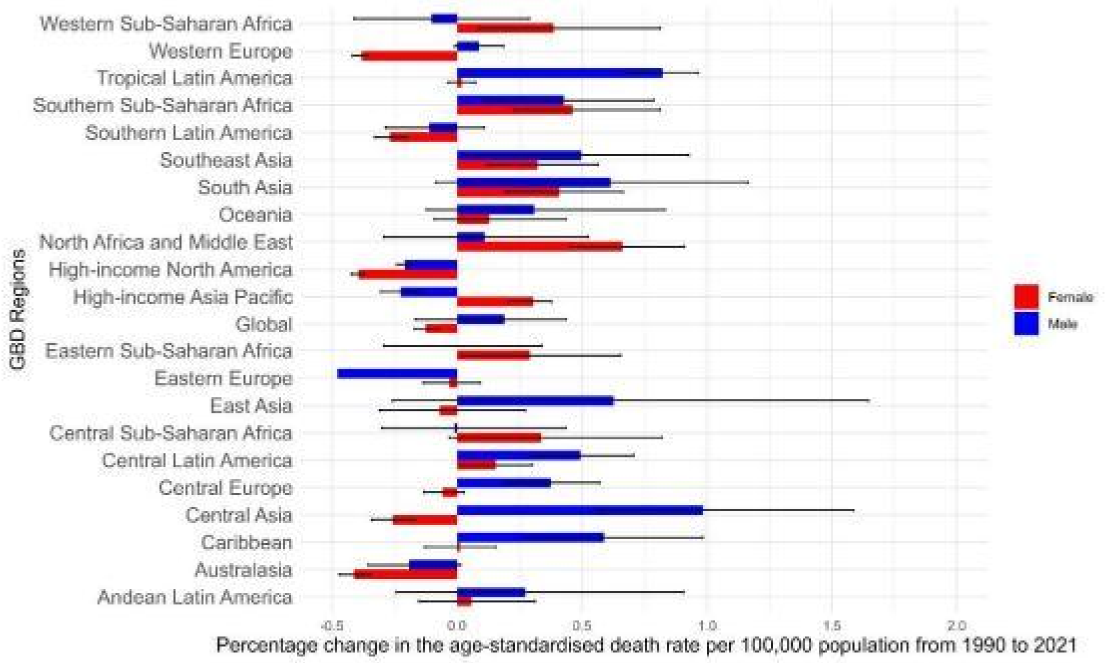

Figure S5: Percentage Change in Age−Standardized Point Death Rates for Breast Cancer by Sex from 1990 to 2021

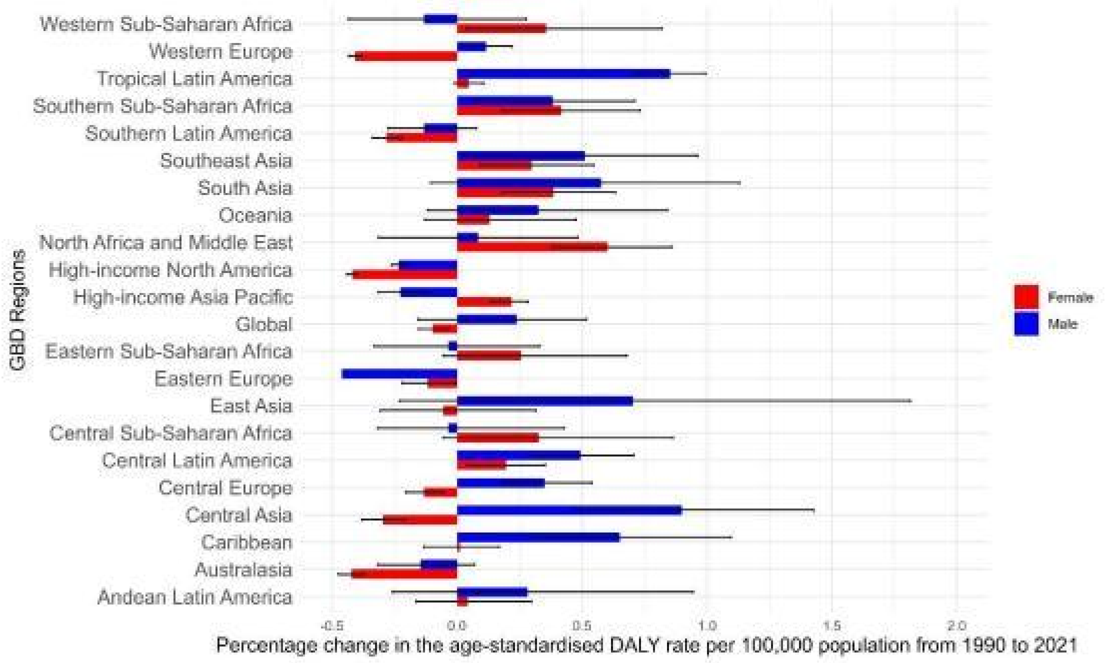

Figure S6: Percentage Change in Age−Standardized Point DALY Rates for Breast Cancer by Sex, 1990 to 2021

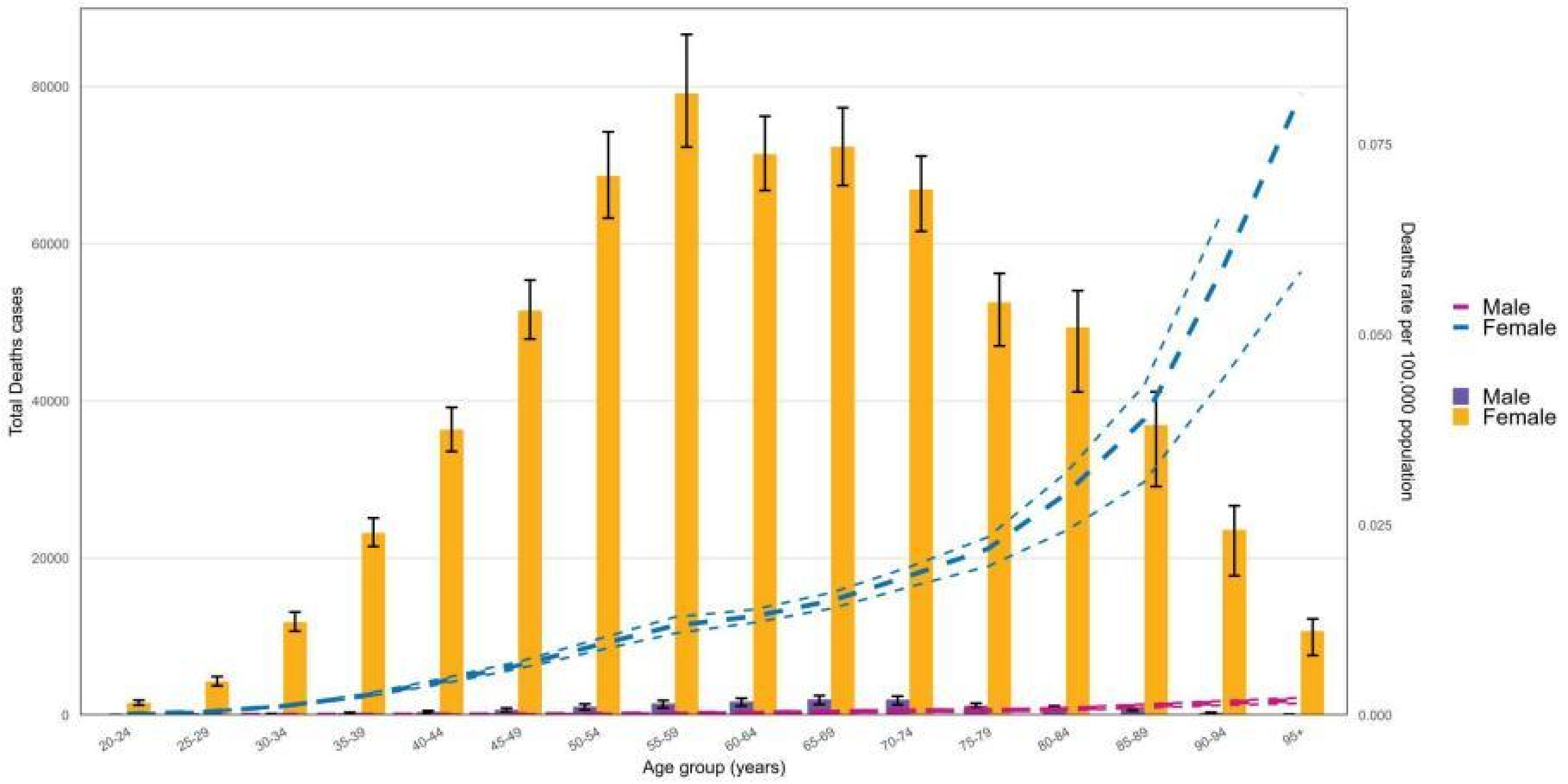

Figure S7: Global death of cases and death of breast cancer per 100,000 population in 2021, by age and sex. Lines indicate prevalent cases with 95% uncertainty intervals for males and females (generated from data available at [https://ghdx.healthdata.org/gbd−results−tool)](https://ghdx.healthdata.org/gbd-results-tool)

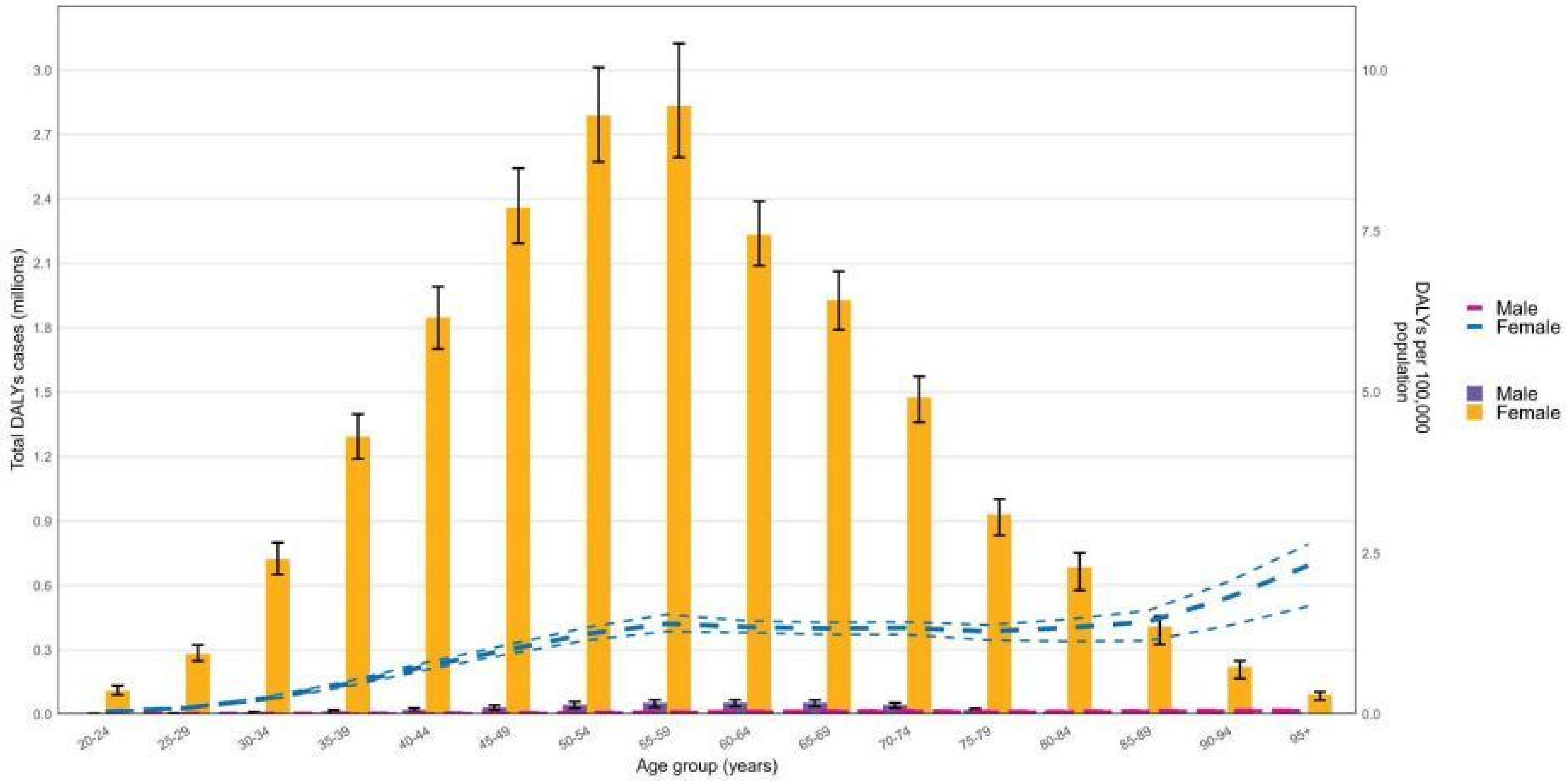

Figure S8: Global DALYs of cases and DALYs of breast cancer per 100,000 population in 2021, by age and sex. Lines indicate prevalent cases with 95% uncertainty intervals for males and females (generated from data available at https://ghdx.healthdata.org/gbd−results−tool)

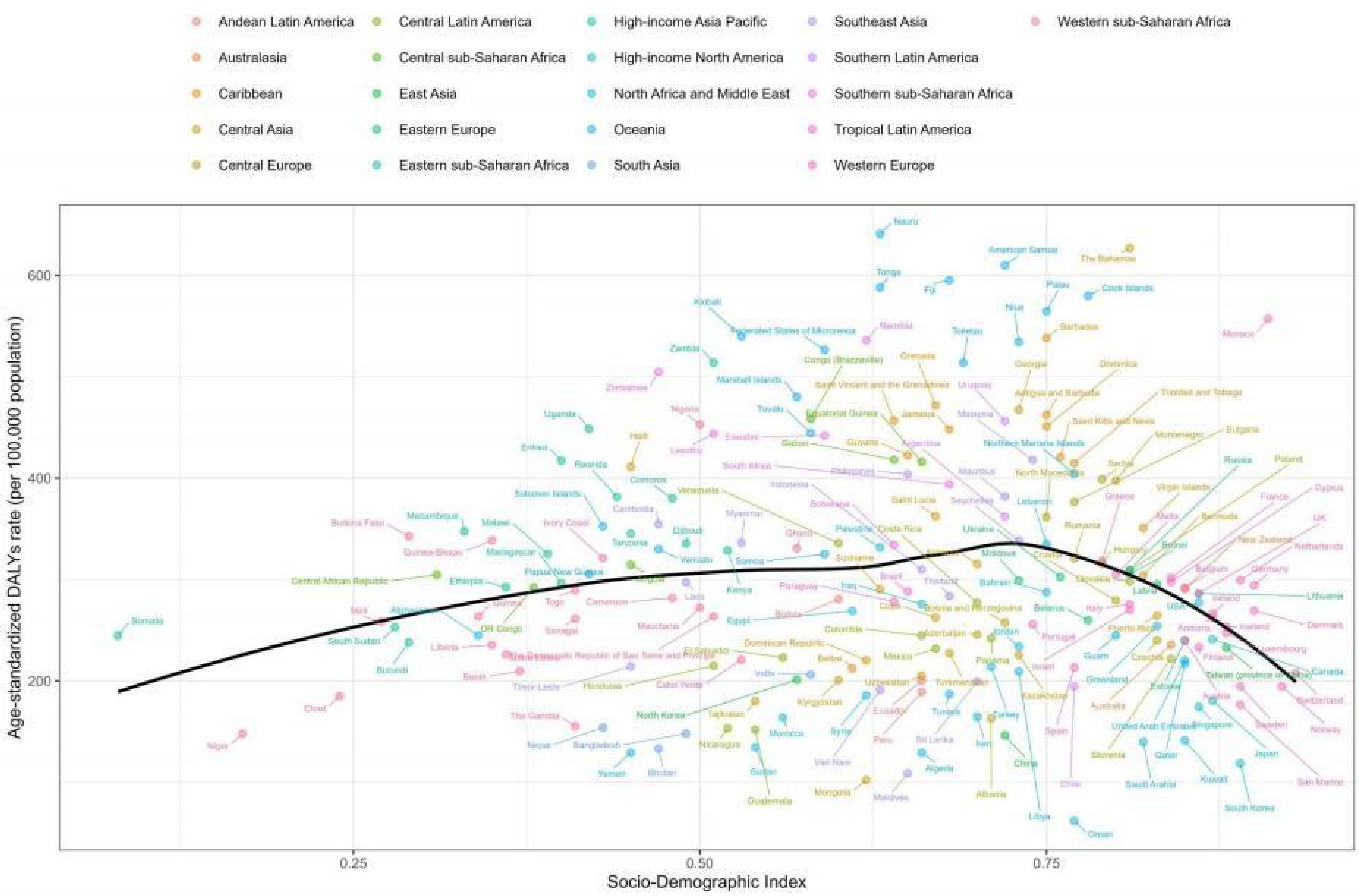

Figure S9: Age−standardized DALY rates for breast cancer (by sociodemographic index) for 204 countries and territories in 2021; expected values based on sociodemographic index and incidence rates for all locations are shown as black lines.and disease incidence rates for all sites are shown as black lines. Each dot shows the observed age Each dot shows the observed age−standardized disability−adjusted life year (DALY) rate for each country in 2021. DALY=disability−adjusted life year. SDI=socio−demographic index (based on results−tools). Demographic Index (generated from data in http://ghdx.healthdata.org/gbd results−tool). SDI=Social Demographic Index (generated from data in results−tool).

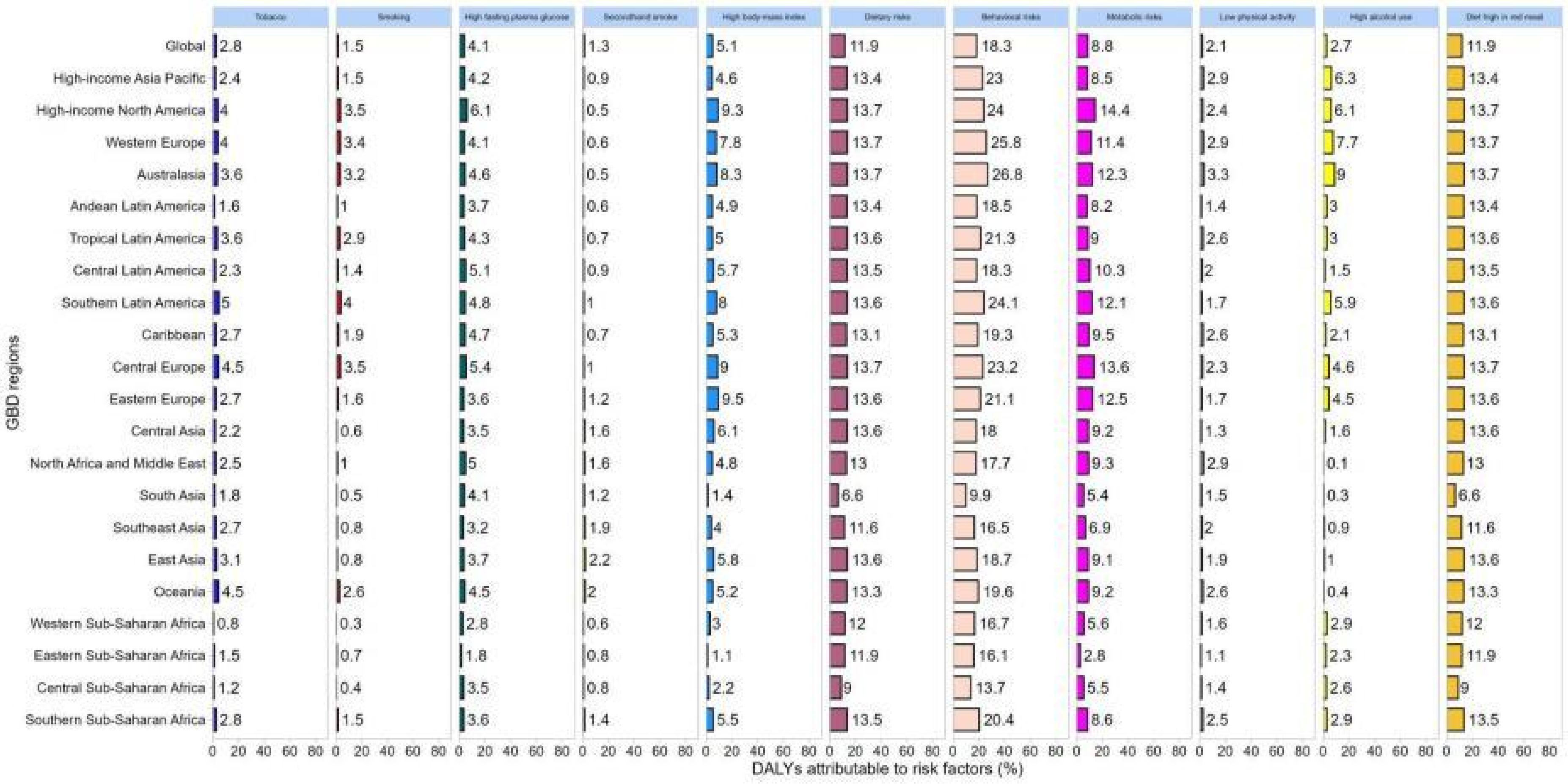

Figure S10: Percentage of Disability−Adjusted Life Years (DALYs) attributable to risk factors due to breast cancer among women in 21 GBD areas, 2021.DALY=Disability−Adjusted Life Year (generated from data provided by http://ghdx.healthdata.org/gbd−results−tool).

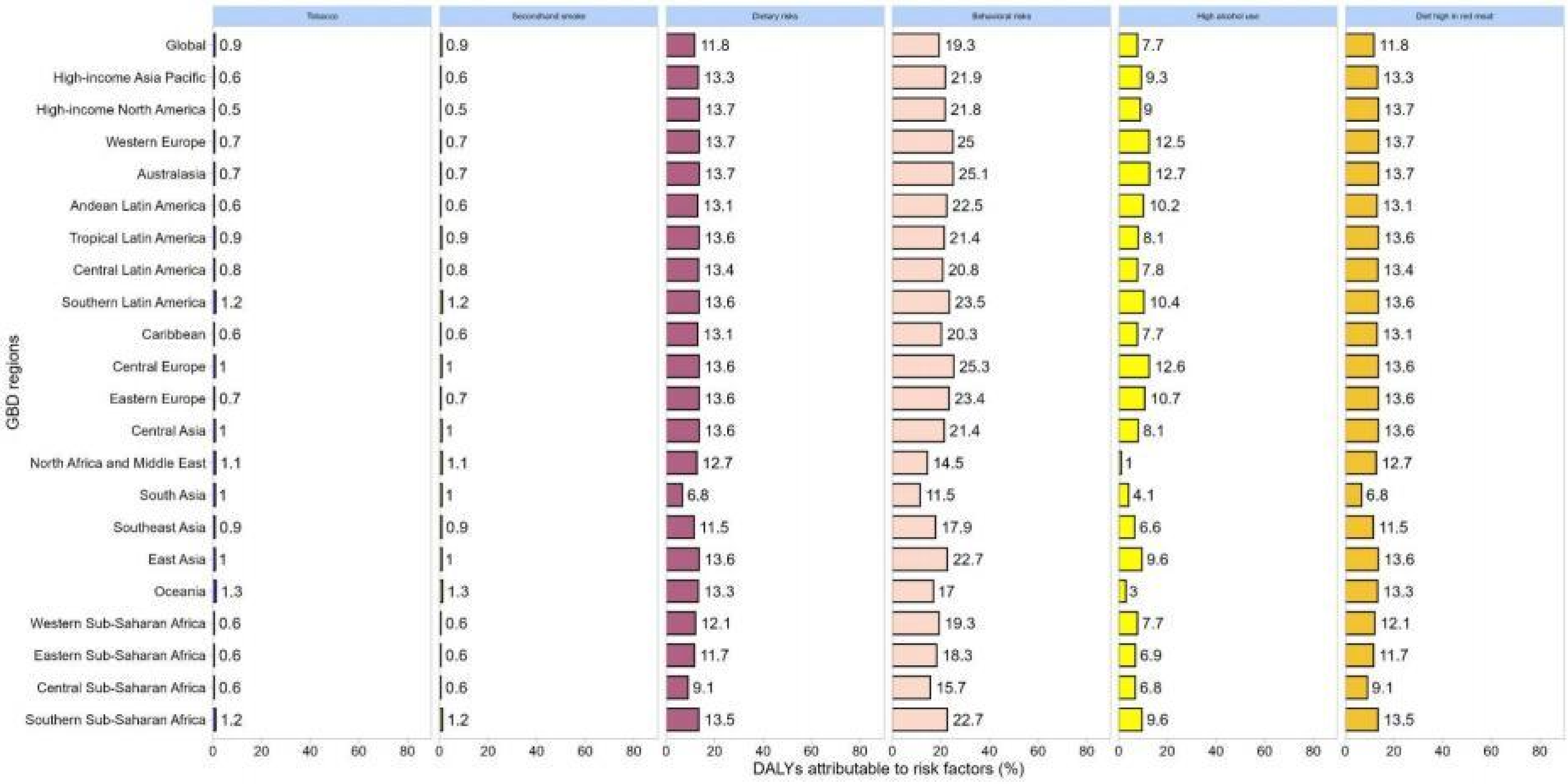

Figure S11: Percentage of Disability−Adjusted Life Years (DALYs) attributable to risk factors due to breast cancer among men in 21 GBD areas, 2021.DALY=Disability−Adjusted Life Year (generated from data provided by http://ghdx.healthdata.org/gbd−results−tool).

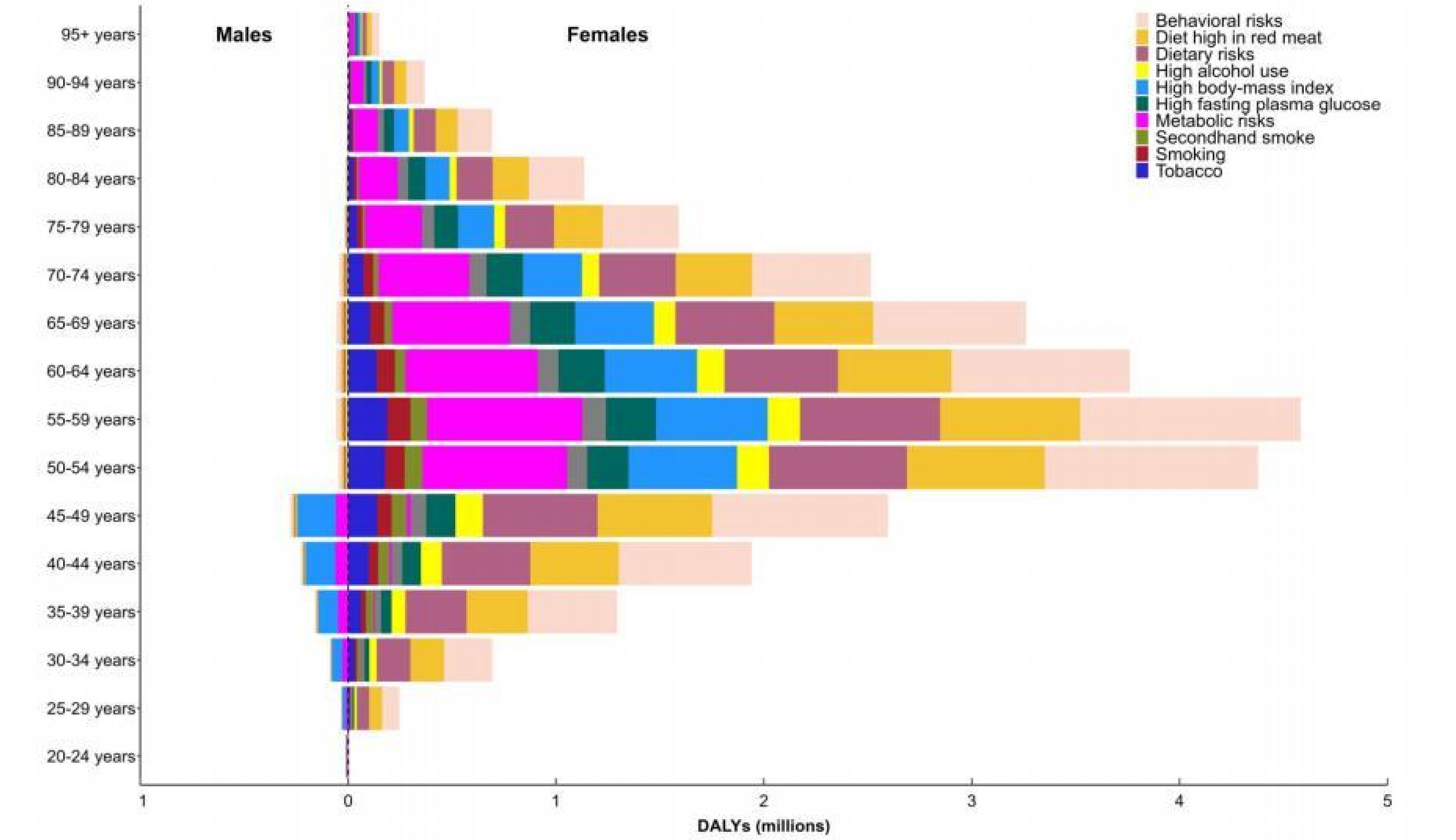

Figure S12: Number of Disability−Adjusted Life Years (DALYs) attributable to each risk factor due to breast cancer, 2021.DALY = Disability−Adjusted Life Year (DALY) (generated based on data in the (generated from data provided by http://ghdx.healthdata.org/gbd−results−tool).
